# Impacts of acidification on brown trout *Salmo trutta* populations and the contribution of stocking to population recovery and genetic diversity

**DOI:** 10.1111/jfb.14054

**Published:** 2019-06-24

**Authors:** Paulo A. Prodöhl, Andrew Ferguson, Caroline R. Bradley, Robin Ade, Colin Roberts, E. J. Keay, Artur R. Costa, Rosaleen Hynes

**Affiliations:** ^1^ Institute for Global Food Security, School of Biological Sciences Queen's University Belfast Belfast UK; ^2^ Dalry, Dumfries & Galloway Scotland UK; ^3^ Dalmellington, Ayrshire UK; ^4^ Marine Scotland, Freshwater Laboratory, Faskally Pitlochry UK

**Keywords:** acid tolerance, adaptation, introgression, microsatellites, population bottlenecks, sympatric populations

## Abstract

Anthropogenic acidification in SW‐Scotland, from the early 19th Century onwards, led to the extinction of several loch (lake) brown trout (*Salmo trutta*) populations and substantial reductions in numbers in many others. Higher altitude populations with no stocking influence, which are isolated above natural and artificial barriers and subjected to the greatest effect of acidification, exhibited the least intrapopulation genetic diversity (34% of the allelic richness of the populations accessible to anadromous *S. trutta*). These, however, were characterised by the greatest interpopulation divergence (highest pairwise *D*_EST_ 0.61 and *F*_ST_ 0.53 in contemporary samples) based on 16 microsatellite loci and are among the most differentiated *S. trutta* populations in NW‐Europe. Five lochs above impassable waterfalls, where *S. trutta* were thought to be extinct, are documented as having been stocked in the late 1980s or 1990s. All five lochs now support self‐sustaining *S. trutta* populations; three as a direct result of restoration stocking and two adjoining lochs largely arising from a small remnant wild population in one, but with some stocking input. The genetically unique Loch Grannoch *S. trutta*, which has been shown to have a heritable increased tolerance to acid conditions, was successfully used as a donor stock to restore populations in two acidic lochs. Loch Fleet *S. trutta*, which were re‐established from four separate donor sources in the late 1980s, showed differential contribution from these ancestors and a higher genetic diversity than all 17 natural loch populations examined in the area. Genetically distinct inlet and outlet spawning *S. trutta* populations were found in this loch. Three genetically distinct sympatric populations of *S. trutta* were identified in Loch Grannoch, most likely representing recruitment from the three main spawning rivers. A distinct genetic signature of Loch Leven *S. trutta*, the progenitor of many Scottish farm strains, facilitated detection of stocking with these strains. One artificially created loch was shown to have a population genetically very similar to Loch Leven *S. trutta*. In spite of recorded historical supplemental stocking with Loch Leven derived farm strains, much of the indigenous *S. trutta* genetic diversity in the area remains intact, aside from the effects of acidification induced bottlenecks. Overall genetic diversity and extant populations have been increased by allochthonous stocking.

## 1 INTRODUCTION

Worldwide, many salmonid populations have become extirpated (Hendry *et al.,* 2003), largely as a result of anthropogenic causes. There is now a particular interest in how best to restore these populations, especially in situations where natural recolonisation cannot occur. Only about a quarter of reintroductions have resulted in self‐sustaining populations (Houde *et al.,* 2015). Restoration stocking failures can occur because the original factors that led to the extinction still exist, or due to random demographic fluctuations (Moritz, 1999). The intrinsic potential for local adaptation in salmonids (Fraser *et al.,* 2011; Garcia de Leaniz *et al.,* 2007) makes restoration particularly challenging with attempts potentially failing due to inadequate adaptive matching of introduced fish (Allendorf & Waples, 1996).

Several approaches have been proposed to overcome restoration failure involving “matching or mixing” (Lesica & Allendorf, 1999). These include using a donor population genetically similar to the extinct one; *i.e*., genetic or ancestry matching (Houde *et al.,* 2015), which assumes that genetically similar fish are likely to be best adapted to the environmental conditions in which the previous population existed. Use of within‐catchment local sources probably gives increased fitness from local adaptation and decreased risks from straying (Garcia de Leaniz *et al.,* 2007). Also, local salmonid sources are likely to share greater genetic similarity with the historic population as a result of common ancestry, although postglacial colonisation by multiple lineages (McKeown *et al.,* 2010) means that this is not necessarily the case for brown trout *Salmo trutta* L. 1758. Another approach involves the selection of a source population from a similar environment; *i.e*., environmental matching (Houde *et al.,* 2015). Such populations may possess genes that are adaptive for the environment of the extirpated population, which may be especially appropriate when the environment has changed substantially in the intervening period. A further stocking option is to use fish from a population with a high level of genetic variation, hence increasing the potential for local adaptation to evolve. High levels of genetic variation can also be produced by mixing fish from multiple genetically dissimilar populations (Houde *et al.,* 2015; Huff *et al.,* 2011). Mixing can involve genetically distinct populations with common ancestry, or from similar environments, so matching and mixing approaches are not mutually exclusive. Mixing may also be appropriate where a single source population cannot sustain the removal of sufficient fish for reintroduction.

In situations where a wild *S. trutta* population is present in reduced numbers, supplemental stocking of fertile farm strain has been used frequently in an attempt to boost the angling catch. The efficacy of stocking farm‐reared *S. trutta*, however, is generally considered to be low (Ferguson, 2007; Pinter *et al.,* 2017). In Britain and Ireland, the farm strains used are often derived solely, or partly, from the first *S. trutta* farms established in Scotland at Solway (1880; 54°58′46″N, 03°39′27″W) and Howietoun (1881; 56°04′20″N, 03°57′10″W), which involved broodstock of Loch Leven (56°12′N, 03°23′W) origin (Armistead, 1895; Maitland, 1887). As stocking with these domesticated strains has been widespread over the past 130 years, the extent to which native gene pools of *S. trutta* have been lost or modified has been the subject of much debate. There is now strong evidence indicating that such genetic changes can affect the fitness, life‐history characteristics and other genetically based aspects of the populations resulting in stocking being counterproductive relative to the aim of increasing *S. trutta* numbers (Ferguson, 2007). Thus, genetic assessment of *S. trutta* populations is important in establishing the effectiveness of stocking in different circumstances. It is also required to determine the extent of introgression by hatchery‐reared *S. trutta* and identify pure indigenous populations of high conservation value.

Effective salmonid conservation and management requires an understanding of the roles of natural and anthropogenic influences on population genetic structure (Small *et al.,* 2007). *Salmo trutta* exhibits complex genetic structuring, with high levels of genetic differentiation often occurring at small geographic scales, both allopatrically and sympatrically (Andersson *et al.,* 2017a, 2017b; Ferguson, 1989; Verspoor *et al.,* 2019). Genetic differences can arise as a result of spawning in different localities and the accurate natal homing typical of salmonids. These spawning groups may diverge genetically over generations as a consequence of genetic drift and natural selection. The varying balances between reproductive isolation produced by homing to natal breeding areas and gene flow among populations caused by successful reproduction of straying individuals (effective straying) results in different levels of genetic population structuring, which may or may not be related to geographic distance among populations (Bond *et al.,* 2014). Compared with other salmonids, relatively little is known of the conditions and timescales required for detectable allopatric and sympatric differentiation to evolve in *S. trutta* (Jorde *et al.,* 2018). While intra and interpopulation genetic variation is a major component of biodiversity, it has received relatively little attention from organisations responsible for the management and conservation of non‐endangered, but ecologically important, species (Mimura *et al.,* 2016).

**Figure 1:**
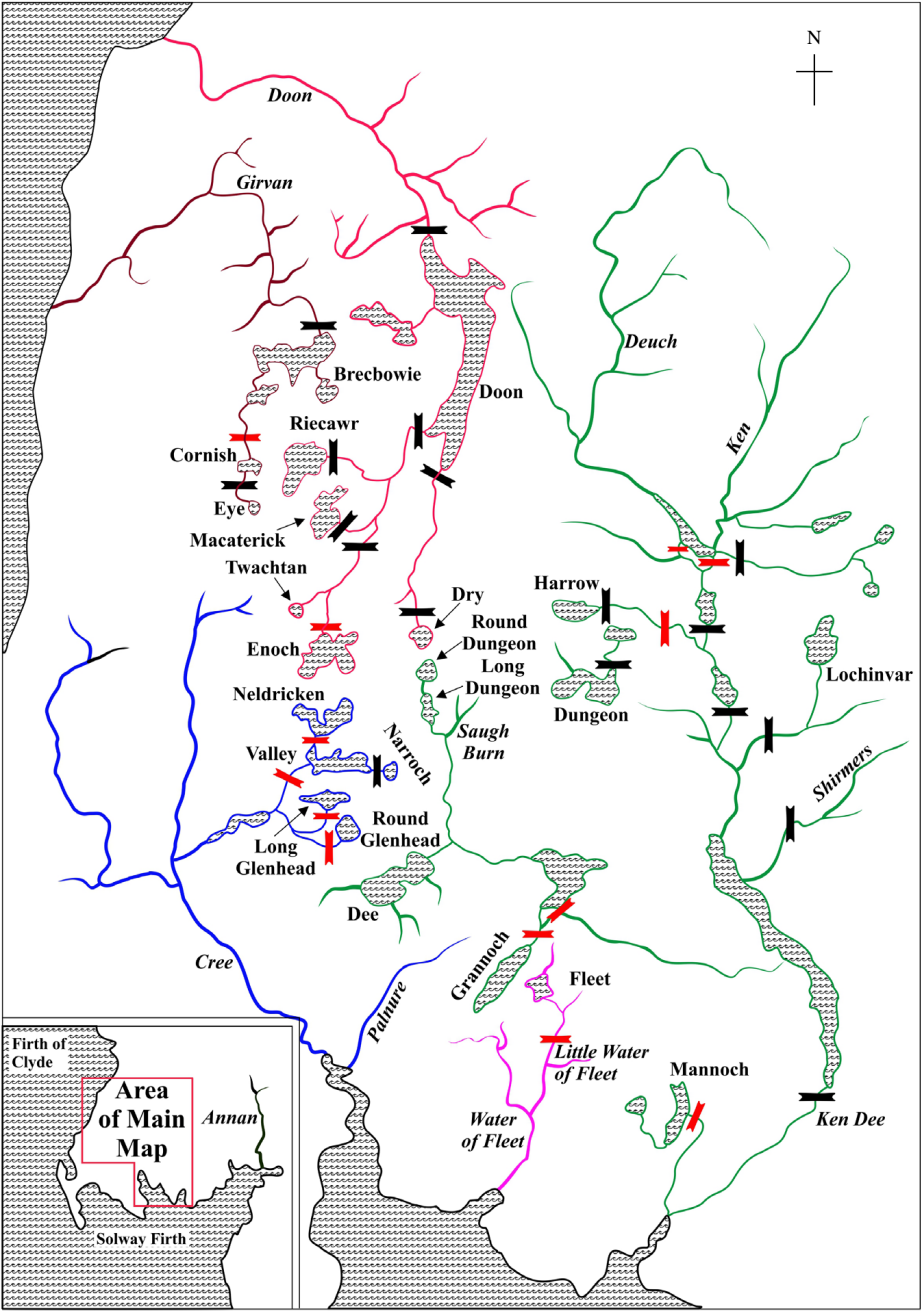
Diagrammatic map (not to scale) of south‐west Scotland showing the relative positions of rivers (in italics) and lochs (in roman font) from which *Salmo trutta* were sampled or are referred to in the text. Additional details are given in Table 1. 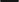, Natural and artificial barriers that are likely to be passable to upstream migrating *S. trutta*, at least for certain sizes of fish and under some water flow conditions; 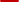, barriers considered impassable to upstream migrants

Many freshwater lochs (lakes) occur in the upland area (200+ m asl) in south‐west Scotland. These range in size from <1 ha to Loch Doon at 820 ha (Figure 1) and angling records indicate that most currently contain *S. trutta*. The lochs are drained by several river systems (Figure 1). In addition to many natural waterfalls, some of the rivers have hydroelectric dams, constructed mainly in the 1930s. These barriers are partially or completely impassable, resulting in many lochs being reproductively and genetically isolated from upstream movement of *S. trutta*. Both river‐resident and anadromous (sea trout) *S. trutta* occur in the lower reaches of these rivers, although artificial barriers have reduced the incidence of the anadromous forms, as elsewhere in Europe (Ferguson *et al.,* 2019).

The area consists largely of granitic rocks, often overlain by peat and poorly‐drained, acidic soils. This base‐poor topography and associated low buffering capacity, together with high rainfall, geographical position and prevailing winds, resulted in the area being the worst affected in Scotland by industrially driven acidification in the latter part of the 20th century (Harriman *et al.,* 1987). Diatom studies of loch substrates indicate that acidification started in the early part of the 19th century (Battarbee *et al.,* 1985), coincident with the early stages of the industrial revolution. The increase in acidity reached its peak in the years after 1950 with the pH falling in several lakes to below 4.5, which is often regarded as the lower tolerance limit for species such as *S. trutta* (Gjedrem & Rosseland, 2012; Jellyman & Harding, 2014). However, no simple pH threshold can be set, as many other factors are often involved. These include heritable tolerance of acidic conditions (Gjedrem & Rosseland, 2012), level of calcium, which reduces the toxic effects of low pH and labile aluminium, the toxicity of which is reduced by dissolved organic carbon (McCartney *et al.,* 2003; Serrano *et al.,* 2008). Extensive coniferous afforestation in south‐west Scotland from 1950s onwards probably exacerbated the acidification due to interception of acid deposition by the forest canopy, this being particularly important in relation to some spawning streams (Harriman *et al.,* 2003). In the early 1980s, UK and international action to reduce the emissions of sulphur and nitrogen from power stations (Kernan *et al.,* 2010) resulted in a *c*. 80% reduction in UK SO_2_ emissions (Helliwell *et al.,* 2011). Improvements in pH and labile aluminium levels took place in the lochs of south‐west Scotland, especially during the second half of the 1980s (Ferrier *et al.,* 2001).

Acidification results in changes to freshwater ecosystems including invertebrate and fish population reductions and extinctions (Flower *et al.,* 1987; Mant *et al.,* 2013). Netting surveys in 1978–79 and 1984–86 (Harriman *et al.,* 1987; Maitland *et al.,* 1987; Turnpenny *et al.,* 1988) failed to detect *S. trutta* in five lochs (Lochs Enoch, Fleet, Narroch, Neldricken and Valley), all of which were known previously to contain *S. trutta* (Harper, 1896; Maxwell, 1878, 1922). Although acidification in Loch Enoch started as early as 1840 (Flower *et al.,* 1987), diatom records indicate acidification in Loch Fleet from *c*. 1960, increasing to an acute level by 1975 (Battarbee *et al.,* 1992). Survival studies in Loch Fleet in 1984 using *S. trutta* eggs and fry showed that these stages could not survive in the loch water as a result of low pH (mean 4.4), low calcium (1 mg l^−1^) and high labile aluminium concentration (200 μg l^−1^; Turnpenny *et al.,* 1988). In all five lochs, waterfalls, impassable to upstream movement of *S. trutta*, prevented upstream recolonisation after environmental conditions improved.

In the 1978–79 and 1984 surveys, low numbers of *S. trutta* were found in many other lochs relative to earlier records. For example, in Loch Grannoch, one of the most acidified lochs, the annual *S. trutta* catch was *c*. 1000 fish in 1940 but this declined steadily to <100 fish in the early 1970s, even with greatly increased fishing effort (Harriman *et al.,* 1987). Loch Riecawr, for which angling catch records exist since the early 20th century, showed a tenfold decline in numbers of *S. trutta* caught per year by anglers from 1925 up to the 1970s, subsequently followed by an increase (Harriman *et al.,* 2001). Catch records (Harriman *et al.,* 2001; McCartney *et al.,* 2003) indicated a rapid natural recovery in *S. trutta* numbers in the lochs during the 1990s in spite of the fact that many of these, Loch Grannoch for example, still remained chronically acidified (Kernan *et al.,* 2010). Continuous pH recording in early 2017 showed pH values in Loch Grannoch from 4.6 to 4.9 (Galloway Fisheries Trust, 2017).

The diverse landscape ecology, water chemistry and anthropogenic influences, including stocking, of south‐west Scotland make it an important and ideal area for studying the effect of these factors on the population genetics of *S. trutta*, which is a UK Biodiversity Action Plan priority species (JNCC, 2010). The main inter‐linked objectives of this study were to determine: (a) the effect of acidification on the contemporary intra and interpopulation genetic diversity and population structure; (b) if restoration stocking has resulted in self‐sustaining populations in lochs where *S. trutta* were considered extinct and the relative success of different strategies for reintroduction; (c) to what extent has stocking with Loch Leven based farm strains resulted in introgression into natural populations; (d) if sympatric sub‐structuring occurs within any of the loch *S. trutta* stocks and how this has evolved; (e) key populations in south‐west Scotland of high conservation or scientific value.

## 2 MATERIALS AND METHODS

### 2.1 Restoration and supplemental stocking history

With the exception of the fish farms and Loch Leven, the locations referred to below are shown on Figure 1. The land surrounding Loch Fleet, a small (17 ha) oligotrophic upland loch, was limed using calcium carbonate powder in 1986 and 1987 producing an almost immediate improvement in water conditions, with a pH close to 7.0 and elevated calcium and reduced aluminium levels (Turnpenny, 1992). Following successful egg survival trials, restoration stocking of *S. trutta* was undertaken in May 1987. This involved 300 fish with *c*. equal numbers of: Little Water of Fleet, the outflowing river below the impassable waterfall (age 1+ years wild *S. trutta*); Loch Dee, a large loch on the geographically adjacent Ken‐Dee catchment (first generation hatchery reared offspring; age 1+, 2+ and 3+ years in the ratio 4:2:1); Solway Fish Farm (age composition similar to Loch Dee stock). The *S. trutta* were batch marked by fin clipping to allow identification of the three stocks. In July 1988, a second batch of 220 *S. trutta*, involving the same three stock types and essentially the same age distribution, was introduced. These were marked with individual tags (Turnpenny, 1992). Egg survival experiments were carried out in the years 1988–89 to 1993–94 to check on possible re‐acidification. Eggs from *S. trutta* trapped in the inlet spawning river were used, with the exception of the 1993–94 season when the eggs used were from *S. trutta* captured in a Loch Grannoch tributary (Turnpenny *et al.,* 1995). This potentially introduced Loch Grannoch stock in addition to the three others above, although <1000 eggs were planted in each of the inlet and the outlet rivers.

Improvements in water chemistry in the late 1980s and early 1990s indicated that conditions might be suitable for *S. trutta* reintroduction in the other four lochs in which *S. trutta* appeared to be extinct earlier. In October 1994, 3000 hatchery‐reared age 1+ *S. trutta* produced from Loch Grannoch broodstock were released in Loch Enoch and survived successfully until at least November 1998 (Collen *et al.,* 2000). At the same time, offspring from this source were also stocked into Loch Neldricken and Loch Valley (I. Murray, former Forestry Commission hatchery manager, personal communication). Approximately 1200 hatchery reared age 1+ *S. trutta* of Loch Enoch parentage were stocked into Loch Narroch in 1999 (E.J.K. unpublished data).

Some stocking with farm strain *S. trutta* is known to have been carried out in other lochs and rivers in the area. According to Sandison (1983), Loch Mannoch was previously stocked with a Loch Leven strain of *S. trutta*. Published and anecdotal accounts indicate that farm‐strain stocking had been undertaken in Lochs Dee, Harrow and Riecawr (Harriman *et al.,* 1987). The most recent farm‐strain *S. trutta* stocking has been only in the rivers, which are the main target for anglers, with River Girvan having been stocked until recently (R.A. unpublished data: information obtained from local angling clubs). Stocking with offspring of Riecawr *S. trutta* also took place in the River Girvan catchment (G. Shaw, Forestry Commission, personal communication).

### 2.2 Sampling

Most specimens (age 1+ year and older) were obtained by angling, which was carried out with appropriate permissions and according to angling regulations. Juveniles were provided by authorised fishery professionals from rivers being electro‐fished as part of their ongoing survey activities. Juvenile *S. trutta* specimens were taken from several geographically separated sites within each river to provide an overall river profile. In the case of Rivers Annan, Girvan and Loch Doon, specimens were taken from below and above natural barriers. Adult *S. trutta* were sacrificed by cranial blow as per standard angling practice while juveniles were killed with an overdose of MS‐222 anaesthetic. An adipose‐fin clip or piece of skeletal muscle was taken, either immediately or at the end of the fishing day and stored in 98% molecular‐grade ethanol. Lethal sampling was not considered to be a threat to the populations since most lochs and rivers appeared to have high densities of *S. trutta*, or angling was carried out within normal bag limits.

In total 2420 *S. trutta* specimens were obtained, mainly in 2010, 2011 and 2012 (denoted as contemporary samples). Frozen *S. trutta* specimens from historical loch samples (1982–2002) were available at the Marine Scotland, Freshwater Laboratory Pitlochry. Samples were taken from 23 lochs, the main focus of the study and seven rivers in south‐west Scotland. In addition, samples were obtained from Loch Leven and from Howietoun farm, as the local Solway farm, previously used in the area for stocking including Loch Fleet, was no longer in operation.

Sample location details and associated three‐letter abbreviations, together with sampling year (*e.g*., GRA_82_) where temporal samples were available, are given in Table 1 and locations in south‐west Scotland are shown on Figure 1. Abbreviations used without year subscript refer to the overall combined sample (*e.g*., GRA). For the Loch Fleet analyses only, 20 fry specimens were obtained from the only inlet river (Altiwhat River) and seven specimens from the outlet river (Little Water of Fleet) immediately below the loch (Figure 1). Details of angling fishing effort, number of *S. trutta* caught and background information were recorded for each loch (Supporting Information Table S1). The sector of capture within the loch was recorded for FLE_12_ individuals taken in September. The net position within the loch was available for each individual in the GRA_12_ sample. Chi‐square analysis was used to test for heterogeneity in position of capture for sub‐groups.

**Table 1:** Summary of *Salmo trutta* sampling locations and basic genetic statistics

Catchment	Location	Code^a^	Sampling year	Latitude^b^N	Longitude^b^W	*n*	*N*_A_	*N*_PAR_	*N*_AR_	%ACC *N*AR	*H*_O_	*H*_E_	Full sib families^c^	LDH‐C1*100^d^
Ken‐Dee	Loch Grannoch^§^	GRA_82_	1982	55°00′11″	04°17′03″	23	85	0	5.31	62	0.66	0.61	1×6, 1×3, 4×2	na
Ken‐Dee	Loch Grannoch^§^	GRA_94_	1994	55°00′11″	04°17′03″	22	94	0	5.43	63	0.62	0.61	1×3, 1×2 1×2	na
Ken‐Dee	Loch Grannoch^§^	GRA_02_	2002	55°00′11″	04°17′03″	45								
Ken‐Dee	Loch Grannoch^§^	GRA_10‐12_	2010, 2011, 2012	55°00′11″	04°17′03″	266	111	0.01	5.9	67	0.61	0.63	1×3, 23×2	0.34
Ken‐Dee	River Deuch^§^	DEU	2010	55°14′44″	04°16′56″	77	130	0.14	6.85	80	0.60	0.61	4×2	0.33
Ken‐Dee	River Ken^§^	KEN	2010, 2011	55°14′22″	04°07′29″	61	117	0.2	6.48	75	0.52	0.58	5×2	0.16
Ken‐Dee	Loch Dungeon^§^	DUN	2010, 2011	55°07′41″	04°19′21″	79	83	0.06	4.76	55	0.45	0.44	7×2	0
Ken‐Dee	Loch Harrow^§^	HAR_94_	1994	55°08′47″	04°18′29″	35	113	0.09	5.79	67	0.56	0.57	0	0.03
Ken‐Dee	Loch Harrow^§^	HAR_11_	2011	55°08′47″	04°18′29″	56							3×2	
Ken‐Dee	Lochinvar^+^	INV	2012	55°08′28″	04°06′13″	38	69	0	4.28	69	0.59	0.57	0	0.32
Ken‐Dee	Loch Dee & tributaries^§^	DEE	2010, 2011	55°04′54″	04°23′54″	68	147	0.01	7.13	83	0.62	0.66	0	0.12
Ken‐Dee	Loch Round Dungeon^§^	RDU	2011	54°47′26″	04°06′04″	41							1×2	
Ken‐Dee	Loch Long Dungeon^§^	LDU_94_	1994	54°44′13″	04°04′58″	49							1×6, 1×2	
Ken‐Dee	Loch Long Dungeon^§^	LDU_11_	2011	54°44′13″	04°04′58″	44							1×2	
Ken‐Dee	River Shirmers^+^	SHI	2011, 2012	55°02′32″	04°05′55″	68	135	0.26	7.41	86	0.64	0.67	0	0.14
Ken‐Dee	Loch Mannoch^§^	MAN	2010	54°54′59″	04°05′31″	31	126	0.01	7.85	91	0.62	0.64	1×2	na
Fleet	Loch Fleet^§^	FLE_11_	2011	55°00′13″	04°15′10″	167	137	0.02	7.06	82	0.64	0.66	1×5, 1×4, 4×3, 12×2	na
Fleet	Loch Fleet^§^	FLE_12_	2012	55°00′13″	04°15′10″	72	128	0.04	7.07	82	0.65	0.66	2×3, 4×2	na
Fleet	River Water of Fleet^‡^	WOF	2011, 2012	54°29′53″	03°50′20″	105	164	0.11	8.59	99	0.69	0.72	1×4, 4×3, 6×2	0.1
Palnure	River Palnure^‡^	PAL	2011, 2012	54°56′47″	04°25′17″	51	150	0.14	8.66	100	0.68	0.69	2×2	0.1
Cree	Loch Valley^§^	VAL	2011	55°05′55″	04°26′47″	51	70	0	4.15	48	0.46	0.47	1×2	0.82
Cree	Loch Narroch^§^	NAR_00‐02_	2000, 2001, 2002	55°05′56″	04°25′50″	52	66	0	4	46	0.65	0.58	1×13, 1×9, 1×6, 1×5, 1×3, 1×2	na
Cree	Loch Narroch^§^	NAR_12_	2012	55°05′56″	04°25′50″	13	63	0	3.94	46	0.53	0.56	0	na
Cree	Loch Neldricken^§^	NEL_01_	2001	55°07′00″	04°26′51″	23	26	0	1.63	19	0.12	0.12	4×2	na
Cree	Loch Neldricken^§^	NEL_11_	2011	55°07′00″	04°26′51″	53	77	0	4.3	50	0.39	0.43	1×3, 2×2	0.9
Cree	Loch Round Glenhead^§^	RGL	2011	55°05′24″	04°25′48″	74	87	0.03	4.85	56	0.48	0.48	1×2	0.63
Cree	Loch Long Glenhead^§^	LGL	2011	55°05′24″	04°25′48″	53	52	0.07	2.98	35	0.34	0.34	1×2	0.5
Girvan	River Girvan^‡^	GIR	2010	55°16′07″	04°29′18″	44	151	0.36	8.98	104	0.67	0.71	1×6, 2×2	0.06
Girvan	Loch Eye^§^	EYE	2011	55°11′47″	04°29′58″	48	64	0	3.74	43	0.41	0.41	1×3, 1×2	0.83
Girvan	Loch Cornish^§^	COR	2011	55°12′52″	04°30′02″	51	76	0	4.46	52	0.49	0.50	0	0.4
Girvan	Loch Brecbowie^§^	BRE	2011	55°13′59″	04°28′13″	58	88	0	5.01	58	0.52	0.54	2×2	0
Doon	River Doon^‡^	RDO	2010	55°18′24″	04°22′49″	38	140	0.07	8.39	98	0.60	0.69	1×5, 1×4, 2×2	0.25
Doon	Loch Doon^+^	LDO	2010, 2011	55°15′10″	04°22′37″	78	133	0	6.85	80	0.58	0.60	0	0.03
Doon	Loch Enoch^§^	ENO_96_	1996	55°08′05″	04°26′55″	56	84	0	4.99	58	0.62	0.61	1×6, 1×5, 6×3	na
Doon	Loch Enoch^§^	ENO_11‐12_	2011, 2012	55°08′05″	04°26′55″	16	71	0	4.44	52	0.55	0.56	0	na
Doon	Loch Twachtan^§^	TWA	2011	55°08′35″	04°28′49″	20	68	0.02	4.25	49	0.56	0.52	0	0
Doon	Loch Dry^§^	DRY	2011	55°08′07″	04°25′02″	47	72	0.06	4.16	48	0.52	0.53	5×2	0.13
Doon	Loch Riecawr^§^	RIE	2011	55°12′22″	04°28′07″	20	91	0	5.69	66	0.58	0.56	0	0
Doon	Loch Macaterick^§^	MAC	2011	55°11′18″	04°27′06″	51	106	0	6.08	71	0.57	0.56	0	0.03
Annan	River Annan^‡^	ANN	2008	55°03′33″	03°34′01″	39	138	0.17	8.21	95	0.61	0.68	1×8, 1×7	0.38
Leven	Loch Leven	LEV	2011, 2012	56°11′38″	03°22′16″	62	142	0.02	7.89	92	0.64	0.65	0	0
(Leven)	Howietoun fish farm	HOW	2004	56°04′09″	003°57′40″	48	120	0.08	7.07	82	0.65	0.64	3×2	0.08

*Note. n*: Number of specimens in sample; *N*_A_: total number of alleles across 16 microsatellite loci; *N*_PAR_: private allele richness; *N*_AR_: allele richness; %ACC: % of mean allele richness value of fully accessible populations; *H*_O_: observed heterozygosity; *H*_E_: expected heterozygosity; na: no data available. ^§^definitively impassable; ^+^possibly impassable (condition dependent); ^‡^likely passable (barrier status in respect of anadromous *S. trutta* only: for details of other barriers see Figure 1).

a Population sample code with subscript numbers indicating year(s) for temporal samples.

b Position approximate (±1 km).

c Full sib families, number of families × number of full sibs in family, *e.g.,* 4×2 denotes four families each with two full sibs. Full sibs from the same family were found in both RDU and LDU.

d Frequency of *100 allele at *LDH‐C1,* includes data from Hamilton *et al*. (1989).

### 2.3 Genetic data

Genomic DNA was extracted from adipose‐fin or skeletal‐muscle tissue using the Promega DNeasy 96 kit (http://www.promega.com). Samples were screened for 18 microsatellite marker loci (*Ssa*85, *One*102‐a, *One*102‐b, CA054565, *Ssa*416, *One*103, *Cocl‐Lav*‐4, *One*9uASC, CA048828, CA053293, BG935488, *Ssa*D71, *SaSa*TAP2A, MHCI, *Ssa*410UOS, *ppStr*3, CA060177, *Ssa*197) resolved in two multiplex reactions. These markers were chosen from the 38 loci characterised and optimised by Keenan *et al*. (2013a) for *S. trutta* genetic research. Further information about primers, PCR conditions and genotyping is given in Keenan *et al*. (2013a). LDH‐C1* screening of sub‐samples consisting of 20 specimens each from non‐stocked lochs was carried out following the methodology of McMeel *et al*. (2001) and presented as the frequency of the *100 allele. Mitochondrial (mt)DNA screening and interpretation were carried out as detailed in McKeown *et al*. (2010). MtDNA data were primarily included to assist in FLE ancestry determination although several other loch samples were also included to provide baseline data.

### 2.4 Data analyses

Potential full sibling groups were identified by the maximum‐likelihood method implemented in the program COLONY 2.0.5.4 (Jones & Wang, 2010) with the following variables applied: female and male polygamy with no inbreeding; dioecious and diploid; medium run; full likelihood; no updating of allele frequencies; no sibship prior; typing error rate of 0.001. Three replicate runs were carried out in each case and the majority result used where these differed. In accordance with Hansen and Jensen (2005), but taking account of Waples and Anderson (2017), for analyses other than sibship effective population (*N*_e_) estimates, sibling groups were reduced to a maximum of three individuals with the least amount of missing microsatellite data or in numerical sequence otherwise.

All sample pairs were tested for significant genic differentiation using Exact G tests as implemented in GENEPOP 4.7.0 (Raymond & Rousset, 1995), using 10,000 dememorisations, 100 batches and 5000 iterations per batch. Temporal and geographical samples not showing significant genic differentiation in Exact G tests were pooled for subsequent analysis except where there were triangle inconsistencies; *i.e*., sample A = sample B, sample B = sample C, but sample A ≠ sample C. All subsequent analyses, with the exception of *N*_e_ estimates, were carried out on the combined samples.

Observed (*H*_O_) and expected heterozygosity (*H*_E_) were estimated using diveRsity (Keenan *et al.,* 2013b). Genotypic linkage disequilibria and conformance with Hardy–Weinberg equilibrium (HWE) were determined in GENEPOP, using an exact probability test (Markov chain parameters: 10,000 dememorisations, 100 batches, 1000 iterations per batch), with sequential Bonferroni correction (Rice, 1989). Allelic richness (*N*_AR_) and private allelic richness (*N*_PAR_), the number of alleles or private alleles in a sample were estimated using the rarefaction method in HP‐RARE (Kalinowski, 2005). To avoid analytical bias due to a few samples of *n* < 30, analysis was standardised to a common sample size of 60 genes. Samples from natural populations (*i.e*., excluding ENO, FLE, LEV, MAN and NAR (Table 1)) were divided into those locations known to be fully accessible to anadromous *S. trutta* (hereafter accessible rivers) and those from areas inaccessible to upstream migration as a result of barriers (Table 1 and Figure 1). Note that SHI was excluded from the accessible group as although anadromous *S. trutta* occur occasionally (R.A. unpublished data) it is above three adjacent barriers, which restrict upstream movement. Samples were also divided into two groups on the basis of the underlying geology of the loch area (Supporting Information Table S1). Statistical significance of difference between groups was assessed using the Mann‐Whitney *U*‐test. Spearman's rank correlation was used to determine the degree of correlation between *N*_AR_ with other physical, chemical and biological data. Both the Mann‐Whitney and Spearman's tests were carried out using PAST 3.14 (Hammer *et al.,* 2001).

Differentiation for all sample pairs was measured using Weir & Cockerham's *F*_ST_ (Weir & Cockerham, 1984) and by Jost's *D*_EST_ (Jost, 2008), the latter having the advantage of being independent of the level of gene diversity (Jost, 2008), which often leads to an underestimation of the level of genetic differentiation between samples for multi‐allelic microsatellite markers. *F*_ST_ and *D*_EST_ estimates were calculated using the program diveRsity (Keenan *et al.,* 2013b) and tested for significant deviation from 0 (*i.e*., no significant genetic differentiation) by randomising multi‐locus genotypes between pairs of samples with 1000 bootstrap permutations.

To examine the possible effects of historical stocking on contemporary patterns of population genetic structuring, admixed individuals (identified as described below) were removed from samples of natural populations and corrected *N*_AR_, *N*_PAR_, *H*_E_, *F*_ST_ and *D*_EST_ recalculated; *i.e*., these corrected genetic diversity measures were based on the identified pure clusters rather than the original geographically defined samples.

Three independent approaches, based on different model assumptions and strategies for computation (Jombart *et al.,* 2010; Neophytou, 2014; Neuwald & Templeton, 2013), were employed to describe *S. trutta* population genetic structuring. In the first instance, the Bayesian clustering method implemented in the program STRUCTURE 2.3.4 (Pritchard *et al.,* 2000) was used. STRUCTURE analysis followed the hierarchical approach suggested by Rosenberg *et al*. (2002), which facilitates the identification of major genetic groupings (shared recent ancestry) within the data, eventually refining these down to the population level. Within this hierarchical framework, all major groups identified within a given STRUCTURE run were used separately, as starting points for subsequent runs. In each case, STRUCTURE runs were replicated 20 times for each *K* value (number of genetic clusters being tested), which ranged from 1 to 10 using the following variables: length of burn‐in period = 100,000; number of MCMC reps after burn‐in = 100,000; admixture model, allele frequencies correlated models with and without location priors. The Δ*K ad hoc* approach (Evanno *et al.,* 2005), as implemented in STRUCTURE HARVESTER (Earl & vonHoldt, 2012), was used as a guide to identify the most likely number of clusters. Results of replications were then combined into a single population output using the program CLUMPP 1.1.2 (Jakobsson & Rosenberg, 2007) with the Greedy search method with option 2 for random input orders set to 20,000. CLUMPP output files were used to produce STRUCTURE bar plots illustrating membership of individuals to inferred clusters.

The Bayesian analysis of population structure program (BAPS 5.3; Corander *et al.,* 2003, 2008) was used as the second approach to identify clusters of genetically similar individuals and to assign individuals to clusters based on their multi‐locus genotypes, using BAPS's “clustering of individuals” option. Unlike STRUCTURE, which relies on an *ad hoc* statistic to identify the best number of clusters explaining the data, BAPS infers the optimal number of clusters directly (Corander *et al.,* 2004). The program was initially run with all samples for a maximum *K* = 40 to identify the optimal *K*‐value explaining the data. Subsequent BAPS runs were then sequentially carried out for all samples in fixed *K* + 1 steps from *K* = 2 to *K* = best *K* value (as identified in the previous step), to recover hierarchical relationships among population samples comparable to the STRUCTURE hierarchical analysis.

The discriminant analysis of principal‐components method of Jombart *et al*. (2010), which is implemented in the function dapc of the R adegenet package (Jombart, 2008), was used as the third independent analytical approach. The identification of the best number of clusters (or populations) explaining the data was done using find.cluster (with the Bayesian information criterion; BIC) and with a maximum number of clusters set to 50. To minimise potential analytical biases in the dapc analysis, the number of retained PCA were chosen to optimise the α‐score, as recommended in the dapc manual, using the function optim.a.score.

BAPS was used to identify significantly admixed individuals within inferred populations by identifying the original samples or BAPS clusters from which each individual's alleles originate (Corander *et al.,* 2006, 2008) using the output file from the initial mixture clustering. For this admixture analysis, a minimum cluster size of 20 was used in order to remove small groups of outlier individuals. Following guidelines provided in the BAPS manual, runs involved 100 iterations to estimate individual admixture coefficients, 200 reference individuals for each cluster and 20 iterations to estimate the admixture for the reference individuals (Corander & Marttinen, 2006). The main advantage of BAPS over other algorithms (*e.g*., STRUCTURE) to identify admixed individuals is that the program also generates a *P*‐value for each individual. This provides a test statistic for simulated *q*‐values, which is the likelihood that a particular individual is indeed admixed (*i.e*., resulting from recent introgression between local and individuals from other population sources) rather than a true member of the local population. Thus, individuals having *P*‐values ≤0.05 are considered as having significant evidence of admixture (*i.e*., they carry genes derived from other populations irrespective of their exact *q*‐value). In order to examine, in more detail, the potential effects of historical human mediated gene flow due to stocking, a group comprising the accessible parts of rivers and the four groups of samples representing the main catchments (Rivers Ken‐Dee, Cree, Girvan and Doon) were analysed independently. To check for possible supplemental stocking with farm‐reared *S. trutta*, LEV was included in each of these five sample sub‐sets. Given the known stocking history summarised in Section 2.1, GRA was included in the Cree and Doon Catchment sub‐sets and RIE in the Girvan Catchment analysis. For each sub‐set, *K* was chosen by trial and error to be greater than the optimal number of clusters. Samples from individual lochs were examined separately using BAPS and STRUCTURE to determine if further population structuring was present beyond that seen in the overall and catchment analyses.

The USEPOPINFO model (Hubisz *et al.,* 2009) in STRUCTURE was used to determine the proportional ancestral contributions to the current FLE stock. The four known potential ancestors, GRA, DEE, WOF and LEV representing the Solway farm strain used (see Section 4.3 for rationale for using LEV), were defined as learning samples and FLE_11_ and FLE_12_ individuals as of unknown origin. STRUCTURE running parameters were as above for the main STRUCTURE analysis except that *K* was fixed at 4 (the number of potential ancestors). BAPS was used in a similar way applying the trained clustering approach (Corander *et al.,* 2008).

To examine the genetic relationships among inferred populations (and also as a further check for the hierarchical STRUCTURE/BAPS analyses), neighbour‐joining (NJ) trees based on Nei's *D*_A_ (Nei *et al.,* 1983) were constructed using POPTREE2 (Takezaki *et al.,* 2010). One tree was constructed using all of the original samples while a second tree was constructed using contemporary south‐west Scotland samples from natural populations only, with BAPS determined admixed individuals removed. Confidence for the tree nodes was assessed by bootstrapping (10,000).

Effective population size (*N*_e_) was estimated using: (a) the bias‐corrected version of the linkage disequilibrium (LD) method (Waples & Do, 2008); (b) by the sibship frequency (SF) method (Wang, 2009); (c) where data were available, by the temporal method of Jorde and Ryman (2007). The NeEstimator 2.01 software (Do *et al.,* 2014) was used for both LD and temporal methods. Allele frequency criteria of ≤0.05, 0.02 and 0.01 were used. Jackknifing over loci was used to obtain 95% confidence intervals for the estimates. For the temporal method, a generation time of 3 years was used based on observations of maturity in the samples obtained (authors’ unpublished data). The SF method was carried out using Colony 2.0.5.4 (Jones & Wang, 2010). Correlation between *N*_e_ values obtained using the LD and SF methods was tested using Spearman's correlation coefficient as above. It should be emphasised that the primary aim of the *N*_e_ analyses was not to accurately determine *N*_e_ but rather to identify populations where values are or were low, such that increased genetic drift would be expected.

## 3 RESULTS

While full sibs were observed in 69% of the samples examined, the actual number of full sib families in each case was small, with 73% of these consisting of two sibs only (Table 1). Comparisons of contemporary temporal samples from the same locality taken in 2010, 2011 or 2012 (Table 1), showed only FLE_11_ and FLE_12_ to have significant differentiation in allelic distribution (*G*‐test *P* < 0.01). For the historical samples, GRA_94_ and GRA_02_ did not differ significantly. However, GRA_82_ and GRA_10‐12_ were significantly different from both of these samples. NAR_00‐02_ and NEL_01_ were significantly different from their contemporary equivalents. All geographical sample pairs were significantly different with the exception of NEL_11_ and VAL and those involving samples from RDU, LDU and DEE (including two inflowing tributaries). The latter were pooled as a single DEE population sample for all but *N*_e_ analyses. However, NEL and VAL were not pooled, due to the significant difference between NEL_01_ and NEL_11_.

Significant departures from Hardy‐Weinberg proportions, in more than two samples for an individual locus, were found for MHC1 (14 samples) and CA053293 (10 samples). MHC1 and CA053293 were thus removed and all other analyses conducted using the 16 remaining loci. Even after Bonferroni correction, most samples were found to display one to four loci deviating from HWE. The exceptions were NEL_11_ and DRY in which nine and eight loci deviated from HWE (Supporting Information Table S2). Significant genotypic linkage disequilibrium (LD) was found at 72 locus pairs in individual samples after Bonferroni correction. Samples with more than two pairs of LD were: DRY (seven pairs); ENO_96_ (five pairs); GIR (six pairs); NAR_00‐02_ (22 pairs); NEL_01_ (five pairs).

The number of alleles observed per locus ranged from two (One102‐a) to 42 (CA048828) with a mean of 16. The total number of alleles (*N*_A_) observed at the 16 loci varied from 26 (NEL_01_) to 164 (WOF), with a mean of 102 alleles (Table 1). In the contemporary samples, allelic richness (*N*_AR_) ranged from 2.98 (LGL) to 8.98 (GIR), with a mean of 5.8, while the NEL_01_ sample had a value of 1.63. Calculating genetic diversity based on samples from natural populations with admixed individuals (Table 2) removed, made little or no difference to most of the values. LGL showed 35% of the *N*_AR_ of accessible river population samples. Other contemporary samples from natural populations with *N*_AR_ values ≤50% of the latter were DRY, EYE, NEL_11_, TWA and VAL. FLE_11_ and FLE_12_ showed 82% *N*_AR_ with respect to accessible population samples, while ENO_11‐12_ & NAR_12_ showed 52% and 46% respectively with respect to these and 82% and 73% with respect to GRA_94‐02,_ the sample temporally closest to when broodstock were taken to produce offspring for stocking. Contemporary samples from natural populations above impassable barriers showed significantly (Mann‐Whitney *U*, *P* < 0.001) lower *N*_AR_ (mean 5.3) in comparison to populations in accessible rivers (mean 8.6). No significant difference was found between the two groups of natural loch populations based on granite or sedimentary geology (Supporting Information Table S2). For the natural loch samples, *N*_AR_ was negatively correlated with altitude (Spearman's *ρ* = −0.66, *P* < 0.01) and positively correlated with loch area (Spearman's *ρ* = 0.73, *P* < 0.01) and total catchment area (Spearman's *ρ* = 0.74, *P* < 0.01). No significant correlation was found between *N*_AR_ and the minimum‐recorded pH for a loch or with the 2010–12 angling catch (Supporting Information Table S1). For all samples, *N*_AR_ was positively correlated with *H*_E_ (Spearman's *ρ* = 0.81, *P* < 0.001).

**Table 2:** Bayesian analysis of population structure program (BAPS) identified admixed individuals of *Salmo trutta* in contemporary samples showing admixture with another *S. trutta* population. All other samples (other than FLE_11_ and FLE_12;_ see Table 4) showed no evidence for the presence of admixed individuals. ENO is 100% GRA origin. LEV was included in the analysis to represent the farm strain of *S. trutta* used in stocking

Population sample^a^	*n*	Admixture source^b^
DEU	77	LEV (2.6%), KEN (2.6%)
KEN	61	LEV (12.1%), DEU (3.3%)
DEE	202	LEV (0.9%), DEU (2.4%)
SHI	68	LEV (10.3%)
WOF	105	LEV (5.7%)
PAL	51	LEV (3.9%)
VAL	51	GRA (33.3%)
NAR	13	VAL (15.4%)
NEL	53	GRA (13.2%)
GIR	44	LEV (36.4%)
EYE	48	COR (12.5%)
COR	51	EYE (9.8%)
RDO	38	LEV (13.2%)
LDO	78	ENO (3.8%), LEV (1.3%), TWA (1.3%)
DRY	47	LDO (14.9%)
RIE	20	LEV (5%)
ANN	39	LEV (2.6%)

Note. *n*: sample size.

a BAPS inferred cluster (population) from initial mixture analysis (sample abbreviations are given in Table 1).

b % admixture from other sources.

The lowest *D*_EST_ value overall (Supporting Information Table S3) was NEL_11_ *vs*. VAL (0.003), which was not significant (95% C.I. –0.003 to 0.012). Values between the various river population samples accessible to anadromous *S. trutta* were also within the lower end of the scale and ranged from 0.024 (95% C.I. 0.01–0.043) for PAL *vs*. WOF to 0.063 (95% C.I. 0.028–0.104) for ANN *vs*. GIR. The value (0.021) between the two most distant (*c*. 400 km sea distance) river samples, ANN *vs*. RDO, was not significantly different from zero (95% C.I. –0.005 to 0.055). Conversely, samples GRA and DEE, from lochs separated by a river distance of *c*.15 km without any barrier preventing unidirectional GRA to DEE gene flow, showed a *D*_EST_ value of 0.137 (95% C.I. 0.115–0.162). The highest values in multiple comparisons of contemporary natural populations were those involving COR, EYE, GRA, LGL and RGL, with the highest being comparisons related to LGL (*e.g*., LGL *vs*. EYE 0.608; LGL *vs*. COR 0.575; LGL *vs*. GRA 0.536). Pairwise *D*_EST_ values involving GRA, ENO and NAR, including temporal samples, were either very low or not significant (Supporting Information Table S3). LEV, HOW and MAN also showed low values (LEV *vs*. HOW 0.033; LEV *vs*. MAN 0.047; HOW *vs*. MAN 0.05). *D*_EST_ involving LEV and the accessible rivers (ANN, GIR, PAL, RDO and WOF) ranged from 0.059 (95% C.I 0.034–0.087) for GIR to 0.109 (95% C.I. 0.075–0.156) for ANN. As expected, *F*_ST_ values were of lower magnitude but were highly correlated with *D*_EST_ (Spearman's *ρ* = 0.92, P < 0.001). Some estimates found to be statistically significant for *D*_EST_ were not significant for *F*_ST_ (*e.g*., NAR_00‐02_ *vs*. NAR_12_ and RDO *vs*. GIR) although heterogeneity *G*‐tests for allelic frequency differences concurred with *D*_EST_ *‐*tests (data not shown).

**Figure 2:**
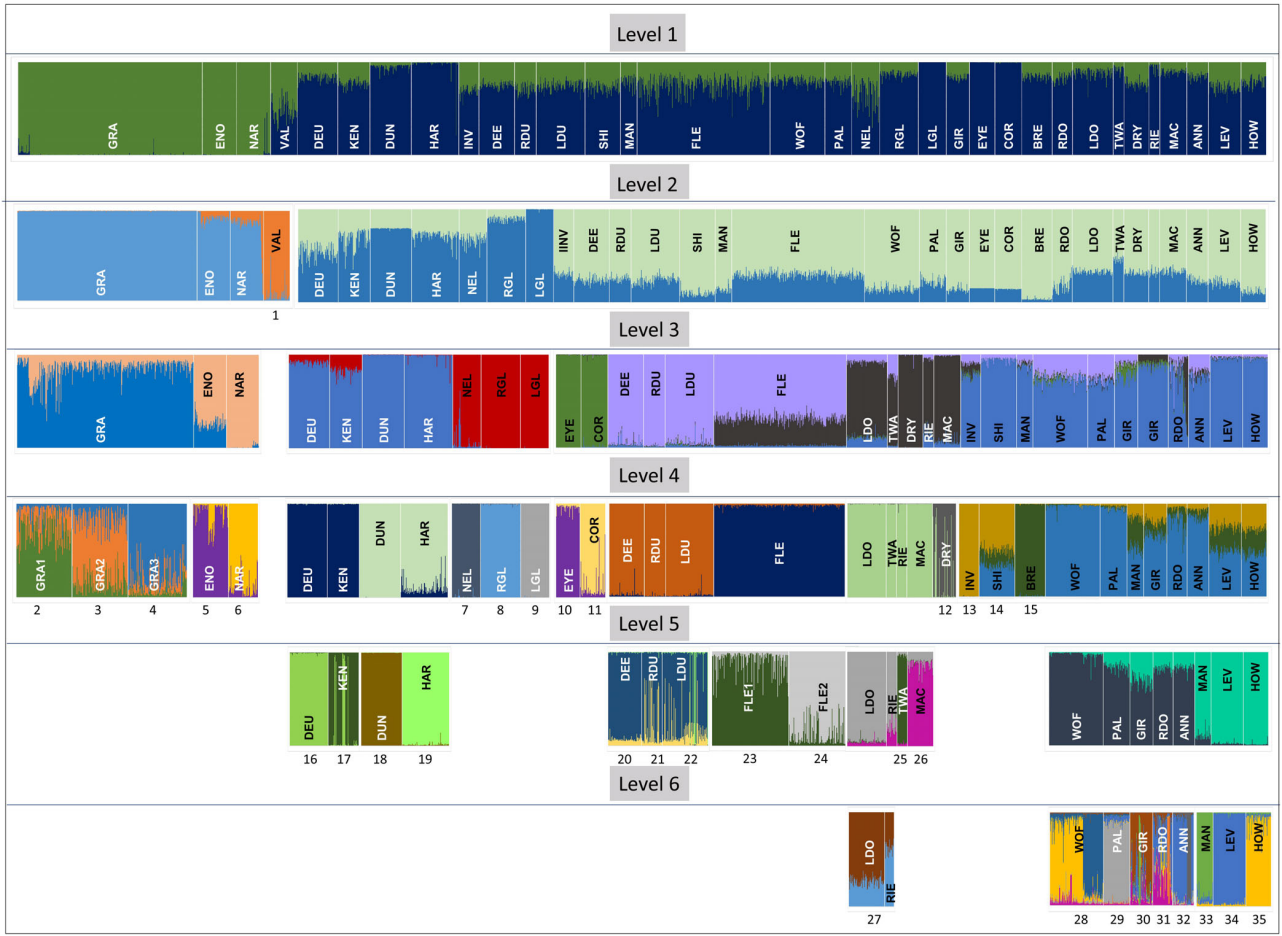
Diagram of hierarchical STRUCTURE analysis of *Salmo trutta* samples examined. Colours represent distinct genetic clusters but note that the colour scheme is random at each hierarchical level. Numbers represent final putative populations identified by the analysis

The three independent methods used to examine *S. trutta* population structuring yielded similar results. Only the results of the STRUCTURE hierarchical analyses are shown (Figure 2). At the most basic level of the STRUCTURE hierarchical analysis, 36 clusters (or inferred populations) were identified (Figure 2 and Supporting Information Figure S1, which gives a diagrammatically simpler representation). This is similar to both BAPS and dapc analyses (33 and 29 clusters). All main clusters were consistent among the three methods with differences relating only to the degree of splitting of genetically similar samples. However, BAPS showed further splitting in some clusters when individual catchments were examined separately for the Admixture analysis (see below).

The first hierarchical level of STRUCTURE clustering indicated two main groups: group 1, GRA, ENO, NAR and VAL; group 2, all other samples. Using a location prior for the analysis, VAL (mean *q* = 0.51) was placed in group 2. Without a location prior, VAL fell into group 1 (mean *q* = 0.52). BAPS analysis assigns both VAL and NEL to the same group even without spatial information. Also, VAL and NEL_11_ were not significantly different in allelic frequencies and *D*_EST_ value (see above). No other STRUCTURE differences were seen with and without location prior information. At the next level, group 2 subdivided into two groups. The first, which comprised samples from the River Cree system and the upper River Ken‐Dee, split into two further clusters representing these two river systems; (DEU, KEN, DUN, HAR) and (NEL, RGL LGL). The second larger group then splits hierarchically into clusters and individual population samples, with the exception of LDO and RIE. Notably DEE and FLE form a single cluster at this level. INV, SHI, MAN, WOF, PAL, GIR, BRE, RDO ANN, LEV and HOW initially form a single group, with INV plus SHI and BRE then splitting off. The remainder split into the accessible rivers (WOF, PAL, GIR, RDO, ANN) and LEV related samples (LEV–MAN–HOW). GRA split into three groups and FLE into two. The unrooted Nei's genetic *D*_A_ NJ tree (Supporting Information Figure S2), based on all samples, largely confirms the groupings and sample hierarchy of both the STRUCTURE and BAPS analyses. The equivalent NJ tree (Supporting Information Figure S3), based on contemporary samples only from natural populations with admixed individuals removed (see below), had similar groupings and with slightly increased bootstrap support in most cases.

**Figure 3:**
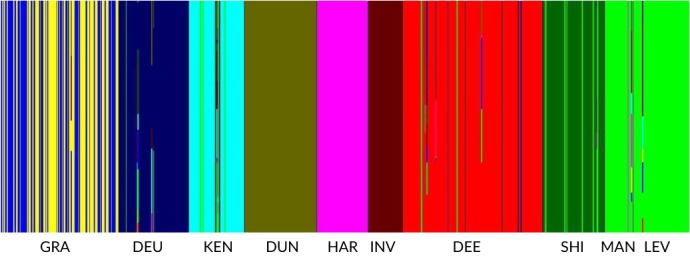
Bayesian analysis of population structure program (BAPS) admixture analysis of Ken‐Dee *Salmo trutta* samples together with Loch Leven (LEV), which represents the farm strain used for stocking. Colours represent distinct clusters. Note LEV (farm) admixture especially in Rivers Ken (KEN) and Shirmers (SHI), and also in River Deuch (DEU) and Loch Dee & tributaries (DEE). GRA, Loch Grannoch; DUN, Loch Dungeon; HAR, Loch Harrow; INV, Lochinvar; MAN, Loch Mannoch

**Figure 4:**
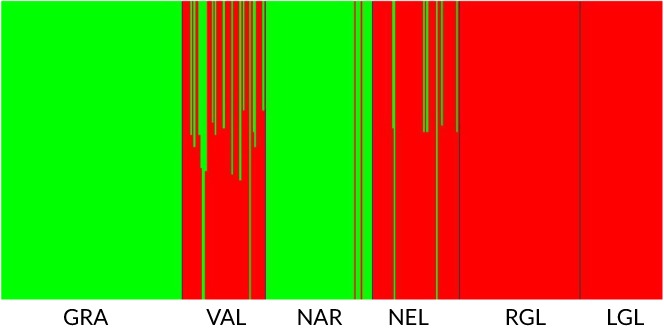
Bayesian analysis of population structure program (BAPS) admixture analysis of Cree *Salmo trutta* samples together with Loch Grannoch (GRA) as known origin of stocked *S. trutta*. Colours represent distinct clusters. Note GRA admixture in Loch Valley (VAL) and Loch Neldricken (NEL) and absence of admixture in Loch Round Glenhead (RGL) and Loch Long Glenhead (LGL). *n.b*. Loch Narroch (NAR) is of GRA ancestry but with two VAL individuals that are probably recent immigrants

BAPS admixture analysis of contemporary samples excluding ENO, NAR and FLE (Table 2) identified admixture in 17 inferred populations. LEV, representing farm strain *S. trutta*, was identified as the likely source for 46% of this admixture (Figure 3). LEV admixed individuals were found mainly in the rivers; *e.g*., GIR (36.4%) but with low frequencies in DEE (0.9%), LDO (1.3%) and RIE (5%). GRA admixture involved VAL (33.3%) and NEL_11_ (13.2%; Figure 4). In the remaining cases, where other admixed individuals were noted, these involved putative sources from the same catchment. The LDH‐C1*100 allele frequency ranged from 0 to 0.9 (Table 1). GRA_10‐12_ had a LDH‐C1*100 allele frequency of 0.34 while in NEL_11_ and VAL these were 0.9 and 0.82 respectively (Table 1). Assuming a native frequency of 1.0 in NEL and VAL, based on allele frequency proportionality (Taggart & Ferguson, 1986) the maximum overall genetic contribution of GRA would be 27% to VAL and 15% to NEL_11_, similar to the BAPS admixture results. The mtDNA haplotype 4.7 was present in GRA_10‐12_ at a frequency of 0.643 and haplotype 3.8 at a frequency of 0.262 (Table 3). However, both NEL_11_ and VAL were fixed for haplotype 3.8, indicating, at most, a limited maternal contribution from GRA.

**Table 3:** Mitochondrial (mt)DNA frequencies in *Salmo trutta* samples from Loch Fleet (FLE) and potential progenitor stocks together with Loch Doon (LDO), Loch Neldricken (NEL) and Loch Valley (VAL). See McKeown *et al*. (2010) for details of haplotypes, except 23.7, which has not been described (R.H., unpubl. data)

Sample^a^	*n*	mtDNA haplotype frequency											
		1.3	2.6	3.7	3.8	4.7	5.9	6.5	7.6	9.3	14.3	22.8	23.7
FLE^b^	242	–	–	0.087	0.360	0.140	0.124	0.145	0.087	–	0.004	0.008	0.045
FLE1	94	–	–	0.096	0.301	0.204	0.011	0.247	0.140	–	–	–	–
FLE2	120	–	–	0.075	0.433	0.083	0.200	0.067	0.033	–	0.008	0.016	0.083
FLE1A	73	–	–	0.110	0.370	0.110	–	0.274	0.137	–	–	–	–
FLE 1B	15	–	–	0.067	0.067	0.400	0.067	0.200	0.200	–	–	–	–
FLE1C	6	–	–	–	–	1.000	–	–	–	–	–	–	–
DEE	53	–	–	0.377	0.340	–	0.019	0.226	0.038	–	–	–	–
GRA_10‐12_	42	0.095	–	–	0.262	0.643	–	–	–	–	–	–	–
WOF	45	0.333	–	0.245	0.200	–	–	0.200	–	–	0.022	–	–
LEV + HOW (farm)^c^	64	0.016	0.063	0.047	0.047	–	–	–	0.811	0.016	–	–	–
LDO	35	–	–	–	0.940	0.060	–	–	–	–	–	–	–
NEL_11_	17	–	–	–	1.000	–	–	–	–	–	–	–	–
VAL	19	–	–	–	1.000	–	–	–	–	–	–	–	–

*Note. n*: number of specimens examined.

a See Table 1 for sample location code.

b Includes specimens from inlet and outlet rivers.

c Includes additional data from McKeown *et al*. (2010).

In independent analyses of samples from individual lochs only FLE and GRA indicated further sub‐structuring. BAPS analyses of FLE_11_ and FLE_12_ samples analysed separately indicated an optimal *K* = 4. With a fixed *K* of 2, as seen in the overall STRUCTURE analysis (Figure 2), three of these groups formed a single group with the remaining group being unchanged. Thus, there are two main groups, with group 1 (FLE1) splitting into three sub‐groups (FLE1A‐C). STRUCTURE analysis of the same data (results not shown) confirms the BAPS results with almost all individuals being similarly assigned. The *D*_EST_ of 0.046 (95% C.I. 0.033–0.06) and *F*_ST_ of 0.028 (95% C.I. 0.038–0.054) estimates between the two main groups were significant. The analyses of FLE_11_ and FLE_12_ as a single sample resulted in similar results for assignment of individuals in the FLE_11_ specimens, but with more differences for the FLE_12_ specimens. This analysis, however, allowed the determination of homologous groups in the two samples. Percentages of individuals belonging to different groups in the FLE_11_ and FLE_12_ samples are shown in Table 4. Assignment of fry and parr from the inlet and outlet rivers (*i.e*., by including them with the FLE_11_ and FLE_12_ loch samples) resulted in all seven juveniles from the outlet river being assigned to FLE1 (outlet group). For the inlet river specimens, all but three were assigned to FLE2 (inlet group) when included with the FLE_11_ sample and all were assigned to FLE2 when analysed in conjunction with the FLE_12_ sample. Of the FLE_12_ group 1 September sub‐sample of *S. trutta*, 28 (90%) were captured in the southern (outlet) sector of the loch while three (10%) were obtained in the northern (inlet) sector of the loch. Conversely for FLE_12_ group 2 fish, eight (29%) were captured in the southern sector and 20 (71%) in the northern sector. Thus, there is a significant departure from random distribution for both groups (*χ* ^2^ *P* < 0.001 and <0.02 respectively).

**Table 4:** Percentage of groups of *Salmo trutta* in Loch Fleet in 2011 (FLE_11_) and 2012 (FLE_12_) samples together with the overall percentage ancestry derived from each of the four known progenitor stocks. Mean values of 20 estimates from USEPOPINFO model in STRUCTURE

	2011 (%)	2012 (%)	Loch Grannoch (%)	Loch Dee (%)	Water of Fleet (%)	Loch Leven^a^ (%)
FLE ALL	100	100	16.3	53.4	12.8	17.5
FLE1	43	22	27.4	58.9	6.1	7.6
FLE1A	34.4	13.6	21.2	63.7	7.1	8
FLE1B	6	4.2	54.9	35.4	4.3	5.4
FLE1C	2.6	4.2	44.6	47.4	2.7	5.3
FLE2	57	88	10.3	50.6	17.1	22

*Note*. FLE ALL, Total of all samples; FLE1, the outlet group, and its sub‐groups; FLE2, the inlet group.

a LEV represented the Solway farm strain used for stocking (as discussed in the text).

The USEPOPINFO model in STRUCTURE indicated that the highest proportion of genes in both FLE1 (58.9%) and FLE2 (50.6%) was from DEE (Table 4). GRA contributed a higher proportion of genes to FLE1 (27.4%) than to FLE2 (10.3%) with the opposite being the case for WOF and LEV (6.1% / 7.6% and 17.1% / 22% to FLE1 and FLE2 respectively). Groups FLE1B (54.9%) and FLE1C (44.6%) showed the highest GRA ancestry, exceeding DEE in both cases. Most individuals were found to be admixed with only four individuals with DEE origin having q values >0.8. Use of trained clustering in BAPS resulted in most FLE1 individuals assigning to DEE with the exception of two individuals from FLE1A that were assigned to WOF and two FLE1B and two FLE1C, which were assigned to GRA. Similar analysis of FLE2 showed all individuals being assigning to DEE. Confirmation that the highest contribution to FLE came from DEE is provided by the *D*_EST_ estimates of FLE with potential ancestors, which were lowest for FLE *vs*. DEE (mean 0.055) for both FLE1 and FLE2 (Supporting Information Table S3) and also by both the STRUCTURE placement (Figure 2) and NJ tree (Supporting Information Figure S2).

The mtDNA haplotype 4.7, present in GRA_10‐12_ at a frequency of 0.643, is the only haplotype that was private to any of the four putative FLE ancestors (Table 3). The frequency of haplotype 4.7 would suggest a GRA contribution of 32% to FLE1 and 13% to FLE2, of similar magnitude to that found for the microsatellite‐based analysis. The estimated contributions of GRA to FLE1A, FLE1B and FLE1C (17%, 62%, 100%) were of similar magnitude to the nuclear estimates with the exception of FLE1C, which may be biased by low sample size. Haplotype 7.6 is present at a frequency of 0.811 in LEV and otherwise only occurs in the putative ancestors at 0.038 in DEE. Its low frequency overall (0.087) and especially in FLE2 (0.033) would suggest a maximum maternal contribution (as DEE could also have contributed this haplotype) of *c*. 11% overall and 4% to FLE2, compared to 18% and 22% respectively based on nuclear markers.

STRUCTURE and BAPS analysis of GRA_10‐12_ specimens revealed three groups. *D*_EST_ & *F*_ST_ values (Supporting Information Table S3) between all pairs of these three groups (GRA1‐2, 1‐3, 2‐3) were significant: *D*_EST_ 0.033 (95% C.I. 0.01–0.056); 0.047 (95% C.I. 0.019–0.053) and 0.023 (95% C.I. 0.014–0.033); *F*_ST_ 0.027 (95% C.I. 0.013–0.045); 0.031 (95% C.I. 0.02–0.045) and 0.005 (95% C.I. 0.001–0.011). Overall, these three groups formed 28%, 37% and 35% of the sample. There was significant heterogeneity in the distribution of the three groups within the loch with GRA_12_ group 1 being present at a greater frequency than either GRA_12_ 2 or GRA_12_ 3 in the northern half of the loch (*χ* ^2^ *P* < 0.02). The latter two groups were not significantly different in their distribution within the loch.

Effective population size (*N*_e_) estimates for the linkage disequilibrium and temporal methods are only given for a minimum allele frequency of 0.01 (Table 5), as this showed the lowest number of ∞ estimates and is also appropriate for sample sizes of the magnitude used ( Waples & Do, 2010). Over all groups there was significant correlation (Spearman's *ρ* = 0.84, *P* = < 0.001 between estimates derived from the two single sample methods (estimates of ∞ excluded). In many cases where sibship estimates are <50, the LD method also indicated a similarly low *N*_e_. With the two methods, *N*_e_ estimates for GRA_82_ were 13 and 19 respectively, the lowest estimates for any of the natural populations. The combined sibship and LD harmonic means over two consecutive samples for GRA_82_, GRA_94_, GRA_02_ and GRA_10‐12_ mirror the temporal estimates involving those years (21, 47 and 102 versus 29, 33 and 95 respectively). FLE_11_ and FLE_12_ combined showed a harmonic mean *N*_e_ estimate with the LD and sibship methods of 101, with the individual years being 146 and 78. FLE1 and FLE2 for FLE_11_ and FLE_12_ combined showed harmonic mean estimates respectively of 19 and 120 with the individual years for FLE1 being 75 and 11 and for FLE2 being 168 and 93. *N*_e_ estimates based on samples from DRY, ENO_96_, EYE, HAR, HOW, LGL, NAR_00‐02_, NAR_12_, NEL_11_ and VAL were low (≤68) with both LD and sibship methods.

**Table 5:** Effective population size (*N*_*e*_) estimates of *Salmo trutta* based on loch samples only and without pooling of temporal samples

		LD		Sib		Temporal method	
Location	Sample	*N*_e_	95%CI	*N*_e_	95%CI	*N*_e_	95%CI
Loch Grannoch	GRA_82_	13	(11–17)	19	(10–39)	–	–
	GRA_94_	31	(21–53)	30	(17–55)	29	(18–72)
	GRA_02_	105	(68–204)	47	(30–75)	33	(21–87)
	GRA_10–12_	276	(220–360)	209	(169–262)	95	(39–∞)
	GRA1	188	(182–771)	93	(68–128)	–	–
	GRA2	207	(117–699)	73	(50–109)	–	–
	GRA3	364	(221–896)	118	(89–160)	–	–
Loch Dungeon	DUN	428	(141–∞)	64	(45–93)	–	–
Loch Harrow	HAR_94_	40	(28–65)	68	(43–115)	–	–
	HAR_11_	195	(112–576)	83	(59–127)	215	(95–∞)
Lochinvar	INV	98	(53–386)	49	(31–75)	–	–
Loch Dee	DEE	1298	(321–∞)	100	(64–190)	–	–
Loch Round Dungeon	RDU	257	(133–1852)	96	(62–168)	–	–
Loch Long Dungeon	LDU_94_	76	(57–109)	61	(39–95)	–	–
	LDU_11_	138	(88–289)	114	(71–219)	568	(206–∞)
Loch Mannoch	MAN	156	(80–1233)	80	(50–167)	–	–
Loch Fleet	FLE_11_	157	(131–192)	137	(105–180)	–	–
	FLE1_11_	77	(60–103)	74	(53–108)	–	–
	FLE2_11_	263	(189–419)	123	(89–167)	–	–
	FLE_12_	104	(85–135)	62	(42–91)	–	–
	FLE1_12_	7	(5–10)	23	(13–46)	–	–
	FLE2_12_	120	(92–167)	76	(52–110)	–	–
Loch Valley	VAL	30	(22–41)	54	(36–82)	–	–
Loch Narroch	NAR_00–02_	6	(4–8)	7	(4–21)	–	–
	NAR_12_	12	(7–22)	28	(14–70)	18	(10–82)
Loch Neldricken	NEL_01_	∞	(14–∞)	21	(11–42)	–	–
	NEL_11_	19	(14–28)	35	(22–56)	6	(4–11)
Loch Round Glenhead	RGL	133	(78–341)	78	(56–111)	–	–
Loch Long Glenhead	LGL	31	(14–94)	44	(28–69)	–	–
Loch Eye	EYE	65	(38–154)	41	(26–66)	–	–
Loch Cornish	COR	241	(101–∞)	53	(34–82)	–	–
Loch Brecbowie	BRE	90	(64–141)	79	(53–123)	–	–
Loch Doon	LDO_11_	1996	(321–∞)	115	(83–162)	–	–
Loch Enoch	ENO	16	(14–19)	15	(8–30)	–	–
	ENO	88	(29–∞)	54	(26–207)	103	(52–23,056)
Loch Twachtan	TWA	113	(41–∞)	34	(19–67)	–	–
Loch Dry	DRY	40	(18–230)	29	(18–51)	–	–
Loch Riecawr	RIE	∞	(232–∞)	105	(55–365)	–	–
Loch Macaterick	MAC	945	(247–∞)	88	(60–134)	–	–
Loch Leven	LEV	644	(289–∞)	134	(93–202)	–	–
Howietoun fish farm	HOW	64	(51–83)	47	(31–72)	–	–

*Note*. GRA1, 2, 3, and FLE 1 and 2 refer to the separate populations identified in those lochs in the GRA_10–12_, the FLE_11_ and the FLE_12_ samples respectively.

LD, *N*_*e*_ based on linkage disequilibrium method with minimum frequency 0.01 as recommended by Waples and Do (2008) for sample sizes of this magnitude; Sib,*N*_*e*_ based on the sibship method (Wang, 2009), assuming non–random mating; Temporal, *N*_*e*_ based on temporal method of Jorde and Ryman (2007), with a minimum allele frequency of 0.01, where the estimate is based on that sample and the preceding temporal one.

## 4 DISCUSSION

### 4.1 Effect of acidification on genetic diversity and population structure

Netting surveys and environmental information suggested that five loch *S. trutta* populations (Lochs Neldricken, Valley, Enoch, Narroch and Fleet) had become extinct as a result of acidification (Harriman *et al.,* 1987; Maitland *et al.,* 1987; Turnpenny *et al.,* 1988). However, the current study shows that a small remnant population of *S. trutta* survived in Loch Neldricken, which expanded when conditions improved and moved downstream to colonise Loch Valley (for details see Section 4.2). However, at least three *S. trutta* populations, which were likely to have been genetically unique, have been lost forever due to acidification. Other populations clearly experienced considerable reductions in numbers. In spite of the many caveats in *N*_e_ estimation, especially in subdivided populations (Ackerman *et al.,* 2016; Ryman *et al.,* 2019; Serbezov *et al.,* 2012; Wang, 2016; Waples *et al.,* 2014), estimates from different methods are consistent and indicate, for example, an overall *S. trutta* N_e_ of <30 in Loch Grannoch in the 1980s and early 1990s. Based on the range of *N*_e_ to census population size (*N*_c_) ratios of 0.06–0.26, determined for an upland Swedish lake (Charlier *et al.,* 2011), the overall number of adult *S. trutta* in Loch Grannoch (115 ha) may have been of the order of 50 to 300 fish in 1982.

As well as this *N*_e_ based estimate of a substantially reduced population in Grannoch, angling records also indicate substantially reduced numbers. Thus, the annual Grannoch *S. trutta* catch was approximately 1000 fish in 1940 but this declined steadily to <100 in the early 1970s, even with greatly increased fishing effort (Harriman *et al.,* 1987). In the lochs where clearly remnant populations survived, netting of Loch Neldricken and Round Glenhead in 1978–79 (Harriman *et al.,* 1987) failed to catch any *S. trutta*, with only one individual being caught in Long Glenhead. Similar netting efforts in other lochs resulted in up to 99 *S. trutta* (Supporting Information Table S1). If the *N*_AR_ mean value in the fully accessible *S. trutta* populations is taken as representing the genetic diversity at the time of colonisation, then above‐barrier populations have lost from 20% to 66% of that diversity. Although the cumulative effect of earlier colonisation and post‐colonisation bottlenecks cannot be discounted, it is likely that this was primarily due to the acidification‐induced bottlenecks in the 1970s. Thus, the samples with the lowest *N*_AR_ (Loch Neldricken (2001) and Long Glenhead) are from the lochs where catches were absent or very low in 1978–79.

The negative correlation of *N*_AR_ with altitude in the natural loch populations probably reflects that the higher altitude populations are all above impassable waterfalls, which prevent upstream gene flow restoring lost gene diversity. In addition, they are in the upper regions of their catchments with no higher populations from which downstream gene flow could occur. Previous studies on salmonids have also shown a decrease in genetic diversity from downstream to upstream (Torterotot *et al.,* 2014).

Loch populations with the lowest *N*_AR_ values had the highest *D*_EST_ and *F*_*ST*_ values in comparisons with other populations. Thus, increased genetic drift in bottlenecked populations, which resulted in a loss of gene diversity_,_ also resulted in greater differentiation between populations. In a compilation of 1112 pairwise population estimates of *F*_*ST*_ in Northern European *S. trutta* populations, Vøllestad (2018) found a mean *F*_*ST*_ of 0.078 with only six pairs exceeding the value of 0.526 found here for the Long Glenhead *vs*. Loch Eye populations. These higher values all involved comparisons of a population isolated above a waterfall in the River Ammerån (Sweden) with populations in other parts of that catchment (Carlsson & Nilsson, 2001). Thus, the non‐accessible south‐west Scotland populations are among the most genetically differentiated *S. trutta* populations in northern Europe, at least as inferred from microsatellites.

In spite of the noticeable effects of genetic drift, many *S. trutta* populations group with others in the same catchment even where gene flow has not been possible for many thousands of years. That is, the native population structure, reflecting independent colonisation of each catchment, has been largely retained. For example, both Long Glenhead and Round Glenhead populations cluster together and with the adjacent Loch Neldricken and Loch Valley native group despite the two being isolated from each other by several natural waterfalls that prevent gene flow in both directions, presumably since the early post‐glacial period, *c*. 13,000 years ago (Gordon & Sutherland, 1993). The isolated upper Ken‐Dee Catchment populations (Rivers Deuch and Ken, Lochs Dungeon and Harrow), although grouping together, cluster with the River Cree ones and not with the lower Ken‐Dee populations. This probably indicates that the upstream areas of these two river systems, the estuaries of which are only 15 km apart, were colonised in the early postglacial period by the same lineage, with the creation of waterfalls by isostatic uplift and erosion preventing further colonisation. Loch Grannoch *S. trutta* are genetically distinct, both in terms of microsatellites and particularly mtDNA, from the other populations in the lower Ken‐Dee system, even from the population in the Loch Dee complex some 5 km distant and with Loch Grannoch to Loch Dee gene flow being possible but not *vice versa* since the 1930s. This could reflect the strong genetic drift that probably occurred in the Loch Grannoch stock as the result of low *N*_*e*_. However, local anecdotal information suggests that Loch Grannoch was originally part of the River Fleet catchment, but it has not been possible to verify this. In addition, the distinctive high frequency mtDNA lineage in Loch Grannoch *S. trutta* would suggest a separate lineage (McKeown *et al.,* 2010).

The lower River Ken‐Dee system populations group with those of the Doon Catchment and with the accessible rivers, which form a tight cluster. This is probably the result of Loch Dee being accessible to anadromous *S. trutta* prior to the construction of hydroelectric dams in the 1930s. Both Loch Dee and Loch Doon populations show high *N*_*e*_ estimates, which could have prevented significant genetic drift since isolation. The lowest levels of interpopulation differentiation and highest levels of gene diversity, were found in the samples from the accessible rivers, all of which have an anadromous component. This probably reflects the higher gene flow that typically occurs among anadromous *S. trutta* populations (Prodöhl *et al.,* 2017; Vøllestad, 2018). Östergren and Nilsson (2012) also found that *N*_AR_ was the best predictor of freshwater *vs*. anadromous life history in *S. trutta*.

### 4.2 Restoration stocking and the re‐establishment of *S. trutta* populations

All of the south‐west Scotland lochs sampled during this study were found to have self‐sustaining *S. trutta* populations as stocking of these lochs has not taken place since1999, at the latest. In addition, there are clear indications from angling records of substantial increases in numbers after the period of chronic acidification (Supporting Information Figures S4 and S5). Contemporary self‐sustaining populations include those of Lochs Neldricken, Valley, Enoch, Narroch and Fleet where *S. trutta* were not caught during netting surveys in the 1970s and 1980s (Harriman *et al.,* 1987; Maitland *et al.,* 1987; Turnpenny *et al.,* 1988). The results indicate that Lochs Neldricken and Valley populations are predominantly the result of natural recovery, with some influence from Loch Grannoch stocking, more so in Loch Valley than in Loch Neldricken. As natural upstream colonisation of Loch Neldricken and Loch Valley could not have occurred due to impassable waterfalls, *S. trutta* must have survived in one or both of these lochs.

The similarity of Lochs Neldricken and Valley *S. trutta* individuals not showing Loch Grannoch admixture to the adjacent Long Glenhead and Round Glenhead populations, which are in an adjacent tributary of the same catchment now isolated in both directions by waterfalls, confirms native origin. Although stocking with juvenile age 0+ year *S. trutta* of Loch Grannoch parentage (as used for stocking Loch Enoch at the same time) was carried out in Lochs Neldricken and Valley, angling in Loch Valley in 1996 resulted in several individual *S. trutta* from 500 to 900 g (C.R., unpublished angling records). Since the largest age 2+ years *S. trutta* captured in Loch Enoch in 1996 was only 207 g (Collen *et al.,* 2000), these Loch Valley *S. trutta* must have been older and thus natural fish.

The high genetic similarity of Lochs Neldricken and Valley *S. trutta* in terms of microsatellite, mtDNA and LDH‐C1* frequencies and microsatellite *N*_AR_ suggests that both lochs share a recent common ancestor. As *S. trutta* movement from Loch Valley to Loch Neldricken is not possible due to waterfalls in the short (400 m) connecting river, this ancestor must have been in Loch Neldricken. This is supported by the fact that *S. trutta* were first caught (1996) in the semi‐enclosed bay of Loch Valley into which the river from Loch Neldricken flows and only later (1999 on) in the main part of Loch Valley (C.R., unpublished angling records). It would appear then that a small population survived in Loch Neldricken, or its afferent rivers and when environmental conditions improved this population expanded rapidly and colonised Valley. The limited influence of GRA *S. trutta* in Lochs Neldricken and Valley contemporary populations is likely due to the number of Grannoch *S. trutta* stocked being small relative to the recovered natural populations. Several salmonid studies have shown that wild prior residents have a competitive advantage over new arrivals especially when the latter are relatively few in number and were hatchery reared (Arismendi *et al.,* 2014; Metcalfe *et al.,* 2003).

Loch Enoch *S. trutta* were shown to be of Loch Grannoch ancestry, thus confirming that *S. trutta* were indeed extinct at the time of the netting surveys in the 1970s and 1980s. There is no evidence of Doon system native fish, the impassable waterfall some 350 m downstream from the loch (Collen *et al.,* 2000) having prevented any natural colonisation. The Loch Narroch 2000–2002 sample, which represents the hatchery reared F_1_ offspring stocked in 1999 and probably some F_2_ offspring, was also completely of Loch Grannoch ancestry. The presence of two *S. trutta* in the Loch Narroch 2012 sample, which were identified as admixed individuals from Loch Valley, may mean that the waterfall between Lochs Narroch and Valley is not totally impassable to upstream migrating fish. Alternatively, as the two lochs are only *c*. 150 m apart, a few *S. trutta* could have been transferred passively by anglers, an occasional practice in south‐west Scotland hill lochs, especially where it is thought that there are no fish in a loch (B. Wilson, local angler, personal communication). Sib and *N*_*e*_ estimates indicated that relatively few parents were used to produce the juveniles for stocking Loch Enoch, with the current population showing reduced genetic diversity compared with its Loch Grannoch *S. trutta* ancestor. Similarly, the Loch Narroch *S. trutta* population showed reduced genetic diversity relative to the Loch Enoch population. Guidelines for setting up hatchery stocks suggest the use of a minimum of 50 males and 50 females to adequately represent the genetic diversity of the original stock (Frankham *et al.,* 2014).

The successful re‐establishment of Loch Enoch *S. trutta*, after an absence of at least 70 years, was in spite of it having borderline water chemistry conditions (*i.e*., mean pH 4.8) (Collen *et al.,* 2000). This successful re‐establishment is therefore likely to be due to a genetically increased tolerance of Loch Grannoch *S. trutta* to acid conditions. Loch Grannoch translocated *S. trutta* fry, together with introduced eggs and subsequent alevins, survived much better than the equivalents from Loch Dee (mean pH 5.2 in 1981; Burns *et al.,* 1984) in common‐garden experiments undertaken in the Loch Enoch outflowing river in 1991 and 1993 (Collen *et al.,* 2000). Loch Dee *S. trutta* have previously been shown to have increased tolerance of low pH compared with *S. trutta* from other waters and a farm strain from higher pH conditions (Battram, 1990; McWilliams, 1982). Acid tolerance is a quantitative trait with large genetic variation among natural populations and with a higher heritability than usually found for fitness traits in fishes (Gjedrem & Rosseland, 2012). *Salmo trutta* survived in Loch Grannoch in spite of a minimum pH of 4.2 and aluminium >300 μg l^−1^ being recorded (Harriman *et al.,* 1987), albeit numbers being much reduced as discussed in Section 4.1.

By 1989, *S. trutta* were well‐established in Loch Fleet and age 0+ and 1+ years fish were found in the inlet and in the outlet downstream of the loch for the entire 7 km above the waterfall, although density was very low after 1 km (Howells *et al.,* 1992). By 1993, the loch *S. trutta* density was some five times that of the stocking density (Turnpenny *et al.,* 1995). *Salmo trutta* from all three deliberately stocked ancestors (Loch Dee, Water of Fleet and Solway farm as represented by Loch Leven), together with Loch Grannoch, have contributed to this successful restoration. Loch Dee was clearly the largest overall contributor to the current Loch Fleet stock even though this loch is in a different catchment. Similar to Loch Fleet, Loch Dee has a river–lake migratory stock with spawning occurring in both the inlet rivers and particularly in a major tributary (Saugh Burn; Figure 1) that flows into the outlet river (I. Murray, personal communication).

As noted above, Loch Dee *S. trutta* have also been shown to have increased tolerance for low pH conditions. In keeping with their lower tolerance of acid conditions, even though a similar number and age range of Solway farm and Loch Dee *S. trutta* were stocked and the majority of the inlet spawning run as captured in the trap in 1987 comprised farm *S. trutta* (Turnpenny, 1992), Solway's overall contribution was low. MtDNA analysis would suggest that female contribution was lower than male contribution for Solway, as has also been reported for farm Atlantic salmon *Salmo salar* L. 1758 breeding in the wild (Fleming *et al.,* 1996). Farm strain *S. trutta* generally show poor survival and reproduction in the wild in both rivers and lakes (Ferguson, 2007; Pinter *et al.,* 2017). In 1988, *S. trutta* of Loch Dee origin were found to be the predominant spawners (Turnpenny, 1992). The contribution of Water of Fleet was also low even though these were from the same river some 10 km downstream below the impassable waterfall and were translocated wild *S. trutta* rather than hatchery reared offspring.

Garcia de Leaniz *et al*. (2007) have argued that, as a result of local adaptation, salmonids from within the same catchment would be genetically similar and thus more likely to be successful for population re‐establishment. However, this region of Water of Fleet is a known anadromous *S. trutta* spawning area (J. Graham, Galloway Fisheries Trust, personal communication) and thus their genetic propensity for anadromy (Ferguson *et al.,* 2019) may have meant that they migrated out of Loch Fleet, the impassable waterfall preventing subsequent return. Burger *et al*. (2000) have shown that life‐history adaptations were critically important for the establishment of river and shore‐spawning populations of sockeye salmon *Oncorhynchus nerka* (Walbaum 1792) in an Alaskan lake. In addition, the pH below the waterfall in the Little Water of Fleet was considerably higher than in Loch Fleet (Harriman *et al.,* 1987) and thus *S. trutta* from this locality may not have been suitable for the lower pH environment.

Loch Grannoch *S. trutta* offspring, in spite of being used only for monitoring hatching success in 1993 and not as part of the original deliberate stocking, contributed to a similar extent overall as Solway farm and Water of Fleet and had a higher contribution than these stocks to the outlet group. Indeed, the Loch Grannoch contribution is surprisingly high given that <1000 eyed eggs were used in each of the inflowing and outflowing rivers in 1993–94 only (Turnpenny *et al.,* 1995). Loch Grannoch *S. trutta* could have been first to mature as age 1+ years in 1995–96 and primarily not until 1996–97 as age 2+ years, at which time the other *S. trutta* would have been well‐established. While stochastic factors may have played a part, the acid tolerance of Loch Grannoch *S. trutta*, as discussed above, is likely to have contributed to their success, especially as by 1994 the pH and calcium concentrations were declining again (Howells & Dalziel, 1995). This was particularly so in the outlet, as only part of the catchment, which included the inflow, was limed (Howells *et al.,* 1992). A minimum pH of 4.6 was recorded in the outlet in 1993 (Turnpenny *et al.,* 1995).

The greater success of *S. trutta* of Loch Grannoch origin in the outlet rather than the inlet may also have been the result of the outlet spawning group being much slower to establish and therefore there was less competition for the Grannoch juveniles. Although fry were detected in the outlet in 1990 and 1991, albeit at much lower densities than in the inlet, none were detected in 1992 and 1993 possibly due to high spring flows washing fry downstream (Turnpenny *et al.,* 1995). However, declining pH is more likely to have been responsible since, in the enclosed egg‐box experiments, poor hatching rates of eggs of Fleet parentage were seen in the outlet in 1991–92 and 1992–93 yet the 1993–94 Loch Grannoch eggs showed high survival to hatching (Turnpenny *et al.,* 1995). The Loch Grannoch populations are inlet spawners (McCartney *et al.,* 2003) so clearly *S. trutta* can quickly change to outlet spawning.

The Loch Fleet *S. trutta* stock showed higher genetic diversity than the 17 other natural loch populations sampled in the area, undoubtedly as a result of four genetically distinct ancestors. Thus the mixing strategy has resulted in higher genetic diversity, which is potentially important in maximising the capacity of a population to adapt to its new environment and to future environmental change (Fraser, 2008). Several authors have argued against a mixing strategy on the grounds that hybridisation between genetically distinct stocks can result in offspring of lowered fitness due to outbreeding depression through loss of local adaptation or the disruption of co‐adapted gene pools in the F_2_ and later generations (Huff *et al.,* 2010). However, it is likely that concerns about outbreeding depression have been overstated as several studies have indicated that, although outbreeding depression can occur in early generations, selection can quickly overcome this and result in hybrid superiority in later generations (Houde *et al.,* 2011; Whiteley *et al.,* 2015; Wells *et al.,* 2019). As demonstrated in Loch Fleet, a mixing approach is likely to be the best option for population re‐establishment, except where there is clear evidence of a donor population with adaptive qualities appropriate to the environmental conditions as is the case with Loch Grannoch. However, a combination of the two strategies can be effectively employed as occurred fortuitously in Fleet.

Self‐sustaining stocks arising solely from restoration stocking are shown to be present in Lochs Enoch, Narroch and Fleet some 12–24 years after the initial re‐establishment; such restoration was the primary objective of the Loch Fleet project (Howells *et al.,* 1992). These successes contrast with the generally reported findings in the literature, which indicate that reintroductions have often failed to yield self‐sustaining naturalized populations (Anderson *et al.,* 2014). However, although stated in general terms, these reports are contrary to the *S. trutta* findings here and the fact that many new self‐sustaining *S. trutta* populations have been established world‐wide (Newton, 2013), suggesting that this species may differ from other salmonids in its ability to establish new populations, possibly as a result of its high genetic diversity and life history plasticity (Ferguson, 1989; Ferguson *et al.,* 2019).

### 4.3 Stocking with farm‐strain *S. trutta*

A population that owes its origin to stocking with a Loch Leven derived farm strain of *S. trutta* is that in Loch Mannoch. This loch was artificially created by construction of a dam in 1919 and is first mentioned for its fishing by Maxwell (1922), the author noting that it is “heavily stocked with *S. trutta*.” In this situation of a newly created loch there would have been few, if any, native *S. trutta* to compete with since an impassable waterfall downstream of the dam would have prevented natural colonisation. Also, at the time of stocking, 100 years ago, the farm strain would have been considerably less domesticated than today (Ferguson, 2007).

The stocking of Loch Mannoch would either have been from Howietoun or, more likely, the Solway farm since it was nearby. As noted above, both Howietoun and Solway were derived from Loch Leven broodstock. Contemporary Loch Leven *S. trutta* show a slightly closer similarity to those of Loch Mannoch than Howietoun. Some selective breeding is known to have taken place in the Howietoun population (Stephen & McAndrew, 1990) which may have resulted in genetic divergence. Small *N*_*e*_ in Howietoun may also have led to increased drift relative to the Lochs Leven and Mannoch populations. Thus, the Loch Leven rather than Howietoun sample was employed in this study as a surrogate for the farm strains used for restoration and supplemental stocking in south‐west Scotland. The unique Leven *S. trutta* genetic signature means that introgression of farm genes in natural populations as a result of stocking can be readily monitored, not just in south‐west Scotland but throughout Britain and Ireland where stocking with fertile Loch Leven derived strains has occurred.

Stocking with Loch Leven based farm strains, both Howietoun and Solway, took place in the period 1975–1995 in a number of south‐west Scottish lochs including Lochs Dee, Doon, Harrow and Riecawr (Harriman *et al.,* 1987; I. Murray, personal communication; D. Ross, Balloch Angling Club, personal communication). However, there is limited indication of contribution as a result of this stocking with only a few individual *S. trutta* in Lochs Dee, Loch Doon and Riecawr showing admixture with Loch Leven. In the Loch Harrow population, where no such admixture was found, several days after stocking with farm *S. trutta* in 1978, 66 dead fish were observed (Harriman *et al.,* 1987). It seems likely that these were farm fish, possibly as a result of being unable to cope with the acidic conditions.

Several studies have shown a decrease in admixture over time in *S. trutta* native populations once stocking has ceased (Harbicht *et al.,* 2014; Valiquette *et al.,* 2014). Stocking with farm strains was more prevalent in lowland regions of rivers in south‐west Scotland, where the pH is higher (Harriman *et al.,* 1987) and which are generally of more interest to anglers in the area than the lochs. Loch Leven *S. trutta* influence was found in all of the rivers examined, in most cases at a low level. The highest influence was found in the River Girvan, which is not surprising as this river is known to have had the most recent stocking, with this continuing up until the early years of this century (local angling clubs, personal communication).

### 4.4 Sympatric populations within lochs

Sub‐structuring (*i.e*., genetically distinct and thus reproductively isolated sympatric *S. trutta* populations) was found only in Lochs Fleet and Grannoch. Two main genetically distinct populations were found in Loch Fleet with an inlet spawning population and an outlet spawning one, the latter comprising three sub‐populations. Reproductive isolation due to inlet (lacustrine – adfluvial) and outlet (allacustrine) spawning occurs in other lakes, *e.g*., Lough Melvin, Ireland (Ferguson, 2004). As only a short stretch of the outlet is available for spawning it is likely that spawning also occurs in the adjacent shores of the loch where suitable gravel is present and where diffuse groundwater flow from surrounding land or wind action can provide sufficient oxygenation for the developing embryos (Whitlock *et al.,* 2014). Thus, the outlet population may comprise individuals from several discrete spawning areas in and around the outlet. Lake shore spawning of *S. trutta* has been demonstrated in several upland Norwegian lakes, especially where groundwater influx occurs and is potentially an important strategy where harsh weather conditions occur; *e.g*., periodic bottom freezing of rivers (Heggenes *et al.,* 2009; Thaulow *et al.,* 2014). These authors found genetic differentiation among juvenile *S. trutta* from separate sites within a lake and between adjacent lake and river juveniles. The heterogeneity of the two main Loch Fleet populations in September with respect to spatial position in the loch relative to the inlet and outlet rivers further emphasises their distinctness. This distribution possibly reflects the movement of *S. trutta* to the areas of the loch adjacent to the spawning streams ready for the spawning migration, which for Loch Fleet has been shown to be in October or early November (Turnpenny, 1992). Alternatively, the two populations may restrict their feeding range within the loch throughout the year.

Founder effects may have contributed to the significant genetic differentiation between the outlet and inlet spawning populations, as well as to the diversity among the three outlet sub‐populations. However, genetic divergence as a result of ancestry (founder effects) cannot be separated from differentiation post establishment, which could have occurred as a result of genetic drift due to low *N*_*e*_, especially in group 1, or selection due to different spawning conditions. Thus, it is possible that the genetic divergence observed could have arisen entirely since colonisation without differential ancestry. Veale and Rusello (2017) found evidence of strong divergent selection between river and shore‐spawning *O. nerka*, with reproductively isolated populations of these two ecotypes having arisen in less than 13 generations. Lucek *et al*. (2014) have shown that hybridisation between lineages can promote adaptive divergence by increasing standing genetic variation.

It is likely that the three Loch Grannoch populations identified in the overall sample represent the three main spawning rivers that contribute recruitment to the loch's *S.trutta* (McCartney *et al.,* 2003). The different spatial distribution of the populations within the loch would support this. However, why should the Loch Grannoch *S. trutta* analysis show genetically distinct spawning populations when the same analysis of other lochs in the area with several spawning rivers does not? Thus, no evidence of sub‐structuring was found in Loch Doon, the largest loch, which has three main spawning rivers together with numerous smaller ones. In addition, there is indication of two distinct gill‐raker groups and associated benthic or pelagic feeding, suggesting trophic segregation in Loch Doon (A.F., unpublished data). Elsewhere such segregation has been shown to result in selection for reproductive isolation with consequent phenotypic and genetic divergence (Bernatchez *et al.,* 2016). Due to its underlying geology, Loch Doon was much less affected by acidification than other lochs and although there is some evidence from anecdotal angler accounts of partial reduction in numbers in the 1980s, the fact that angling continued throughout suggests that the reduction was much smaller than for Loch Grannoch. Continuous *S. trutta* catch records from 1908 onwards are available for the adjacent Lochs Macaterick and Riecawr and while these again show a reduction in catches in the 1970s, moderate catches persisted throughout (Harriman *et al.,* 2001). It is clear from both angling records (see Section 1) and *N*_e_ estimates here from the 1980s and 1990s that *S. trutta* numbers were reduced considerably in Loch Grannoch.

As discussed in Section 4.1, the *N*_e_ of the Loch Grannoch stock was <30 in the 1980s and early 1990s, with the *N*_*e*_ in each Loch Grannoch river clearly being considerably lower still. Where there are two or more spatially distinct spawning areas for *S. trutta* and the *N*_e_ in each area is small then the spawning groups will diverge as a result of genetic drift exceeding gene flow due to straying (Ferguson, 1989). Natal homing would serve to maintain this differentiation and the populations may further diverge as they adapt to local conditions and acquire distinct life histories thereby reducing competition (Hendry *et al.,* 2007). Although most of the larger lochs examined in this study probably have two or more spawning areas, it is only where *N*_e_ is small that sufficient genetic differentiation occurs, resulting in detection with the analyses and number and type of markers used in this study.

Although several studies have reported *S. trutta* genetic structuring in large lake systems (Ferguson, 2004; McKeown *et al.,* 2010; Swatdipong *et al.,* 2010; Verspoor *et al.,* 2019), these appear to be the result of colonisation by multiple allopatrically differentiated lineages or occur in large lakes where gene flow is limited by distance between rivers. In the case of Loch Grannoch *S. trutta*, initial analyses do not indicate any differences in morphology or feeding among the three populations (A.F., unpublished data). Two genetically distinct populations in Lakes Trollsvattnet, Sweden (Palmé *et al.,* 2013), which do not differ in feeding ecology and differ only marginally in morphology (Andersson *et al.,* 2017b), are thought to be reproductively isolated due to respective inlet and outlet spawning, although the results were not fully conclusive and other factors may be involved (Andersson *et al.,* 2017a). Sympatric *S. trutta* populations are likely to be more widespread than hitherto reported as most suitable lakes have not been examined in sufficient detail. Indeed, sympatric populations would be expected in all lakes with multiple spawning locations and in such situations trophic and morphological differentiation would not necessarily be present. However, in the absence of phenotypic differences that allow *a priori* grouping and where the *N*_e_ of each population is large and some gene flow exists, it would require detailed sampling, appropriate molecular markers and rigorous statistical analyses to detect the low‐level genetic differentiation that is likely to be present (Jorde *et al.,* 2018; Verspoor *et al.,* 2019). In such situations, examining differentiation among samples from actual spawning rivers or locations may be more appropriate than a pooled sample from a lake, although the former may be logistically difficult to obtain in some cases, especially where lake spawning is involved.

### 4.5 Conservation and wider scientific importance

From a *S. trutta* conservation standpoint, the most important loch in south‐west Scotland is Loch Grannoch due to its genetic distinctness coupled with its increased tolerance of acidic conditions. Although acidification has been reduced in upland freshwaters in Great Britain (Battarbee *et al.,* 2014), predicted climate change poses a threat to this recovery through an increase in rainfall and the intensity and number of storm events resulting in acidifying sea‐salt deposition, as well as increased nitrate leaching from soils (Kernan *et al.,* 2010). These weather‐related changes could result in the remobilisation of toxic aluminium and other substances present in catchment peats (Battarbee *et al.,* 2014). Increased CO_2_ levels can also result in acidification, an aspect as yet poorly studied in freshwater systems compared with marine ones (Ou *et al.,* 2015). Thus, the Loch Grannoch stock may be an important donor for the restoration, or genetic rescue (*sensu* Frankham, 2015), of further *S. trutta* populations in the future. Loch Grannoch *S. trutta* are of considerable interest for the scientific study of local adaptation and population structuring, given the existence of three genetically distinct populations. A detailed conservation and management plan for Loch Grannoch is urgently required as it was evident during this study that a considerable *S. trutta* harvest occurs through permitted and especially non‐permitted angling due to the loch having a good number of *S. trutta* of larger size than most other lochs in the area (Supporting Information Table S1). Loch Grannoch should be accorded legal protection status (*e.g*., as a Site of Special Scientific Interest; SSSI), which would be supported by other important biological features of the loch as well as the unique *S. trutta* populations.

Populations such as those in Long Glenhead, Round Glenhead, Lochs Neldricken and Valley have demonstrated the ability to survive under severe environmental conditions. They are included in the Merrick Kells SSSI but without any specific reference to *S. trutta* and its management. Although it is often assumed that small populations with low genetic variability have low adaptive potential, Prodöhl *et al*. (1997) reported on genetically monomorphic small *S. trutta* populations from north‐west Scotland that showed no evidence for reduction in fitness. More recently, Fraser *et al*. (2014) found evidence of greater adaptive differentiation in such populations. Mechanisms such as associative overdominance may help to reduce the rate of further decline in genetic variability (Fraser, 2017). The low genetic variability of these River Cree populations makes them valuable for studies on genetic variability and fitness. Round Glenhead has continuous monitoring of water quality and climate, thus enabling integration of environmental and molecular genetic data. Given the admixture with Loch Grannoch *S. trutta*, the Lochs Neldricken and Valley populations are valuable models for the study of the progress of introgression.

Since background data are available on their *S. trutta* reestablishment, Lochs Enoch, Narroch and Fleet are all of scientific interest for studying genetic and ecological change from known starting points and should be protected particularly from further stocking with fertile *S. trutta*. Lochs Enoch and Narroch are included in the Merrick Kells SSSI and so such changes in fisheries management would require permission from Scottish Natural Heritage. Fleet is especially relevant for studying *S. trutta* local adaptation in relation to inlet and outlet river and lake, spawning. These populations are of considerable scientific value in further studies of the pre‐existing adaptation *vs*. adaptive potential strategies for restoration as recommended by Houde *et al*. (2015). Indeed, all of the upland lochs are of scientific interest as they present a series of isolated yet adjacent populations subject to varying physical and chemical conditions and again merit specific *S. trutta* management plans. Their genetic isolation means that they provide independent replicates, ideal for the study of parallel and convergent aspects of local adaptation (Merilä, 2014).

Unlike lakes in many other areas of western Europe, the isolated south‐west Scotland populations also represent native populations with negligible influence from domesticated farm *S. trutta* and are thus of considerably increased conservation importance. They are among the most genetically divergent populations so far described for *S. trutta* in northern Europe with much of the native genetic diversity still intact despite the effects of acidification. Such genetically divergent populations are very important when it comes to conserving overall *S. trutta* diversity (Kelson *et al.,* 2015; Vøllestad, 2018). The existence of the Loch Mannoch population derived exclusively, or nearly so, from a Leven‐based farm strain is of increased conservation importance as both Solway or Howietoun farms are no longer in existence, the latter as of 2017 (J. Taggart, University of Stirling, personal communication), and there are continued threats to the native Loch Leven stock (Winfield *et al.,* 2011).

## AUTHOR CONTRIBUTIONS

P.A.P: Sampling design, data analyses, manuscript preparation; funding. A.F: coordination of sampling, data analyses, background literature & angling record compilation, manuscript preparation, funding. C.R.B: microsatellite and LDH analyses, manuscript preparation. R.A: genesis of study, field sampling, background information & angling records, Figure 1. C.R: field sampling, background information and angling records. E.J.K: preparation of historical samples, manuscript preparation. A.R.C & R.H: mtDNA analyses, manuscript preparation.

## Supporting information


**FIGURE S1.** Simplified STRUCTURE plot of *Salmo trutta* genetic clusters. Each colour represents a distinct genetic cluster but note that the colour scheme is random at each hierarchical level. Numbers represent final putative populations identified by the analysis.

**FIGURE S2.** Neighbour joining unrooted tree based on Nei's (1983) genetic distance (*D*_A_) for *Salmo trutta* population inferred samples. Percentage bootstrap support shown at nodes. Colours refer to catchment colours as per Figure 1 (except for Loch Leven (LEV) and Howietoun (HOW)). For other sampling site locations, see Table 1.

**FIGURE S3.** Neighbour joining unrooted tree based on Nei's *et al*. (1983) genetic distance (*D*_A_) for *Salmo trutta* population inferred contemporary samples. Based on natural south‐west Scotland populations only with admixed individuals removed. Percentage bootstrap support shown at nodes. Colours refer to catchment colours as per Figure 1. For other sampling site locations, see Table 1.

**FIGURE S4.** Number (*n*) of *Salmo trutta* caught per hour by anglers fishing at Round Loch of Glenhead 1989–2011.

**FIGURE S5.** Number (*n*) of *Salmo trutta* caught per hour by anglers fishing at Loch Valley 1996 to 2011.

**TABLE S1.** Background information on lochs from which *Salmo trutta* were sampled.

**TABLE S2.** Summary population sample statistics per locus and overall loci for *Salmo trutta* population samples.

**TABLE S3.** *D*_EST_ and *F*_ST_ pairwise values between STRUCTURE or Bayesian analysis of population structure program (BAPS; GRA and FLE only) inferred populations. In each case, estimates are shown using a colour heat map (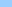
–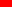, low–high level of between population genetics divergence) below diagonal (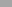). Values in parentheses above diagonal indicate 95% CI for estimates. 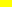, non‐significant pairwise comparisons are highlighted in yellow. *n.b*. Natural populations have had admixed individuals removed. GRA1, 2 and 3, and FLE1 and 2 are the separate populations identified in these lochs with in the former only the GRA10‐12 sample being used in the computation.
