# Supplementary figures and images for: Impacts of acidification on brown trout Salmo trutta populations and the contribution of stocking to population recovery and genetic diversity
Source: J Fish Biol. 2019 Jun 24;95(3):719–42. doi: 10.1111/jfb.14054 (PMC6852074; doi:10.1111/jfb.14054)

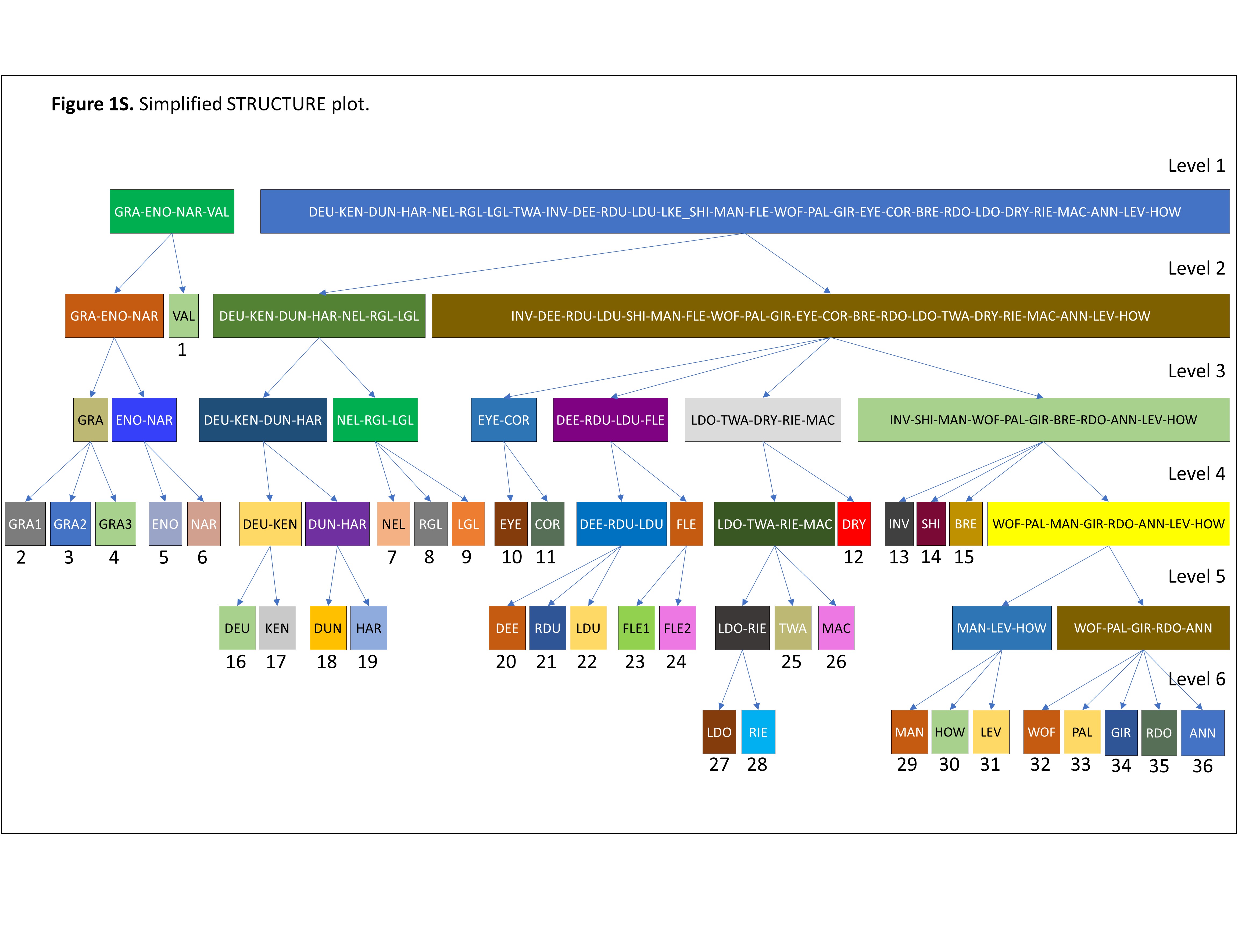

Supplement: Supplementary file 1 — FIGURE S1. Simplified STRUCTURE plot of Salmo trutta genetic clusters. Each colour represents a distinct genetic cluster but note that the colour scheme is random at each hierarchical level. Numbers represent final putative populations identified by the analysis. [file JFB-95-719-s001.jpg]

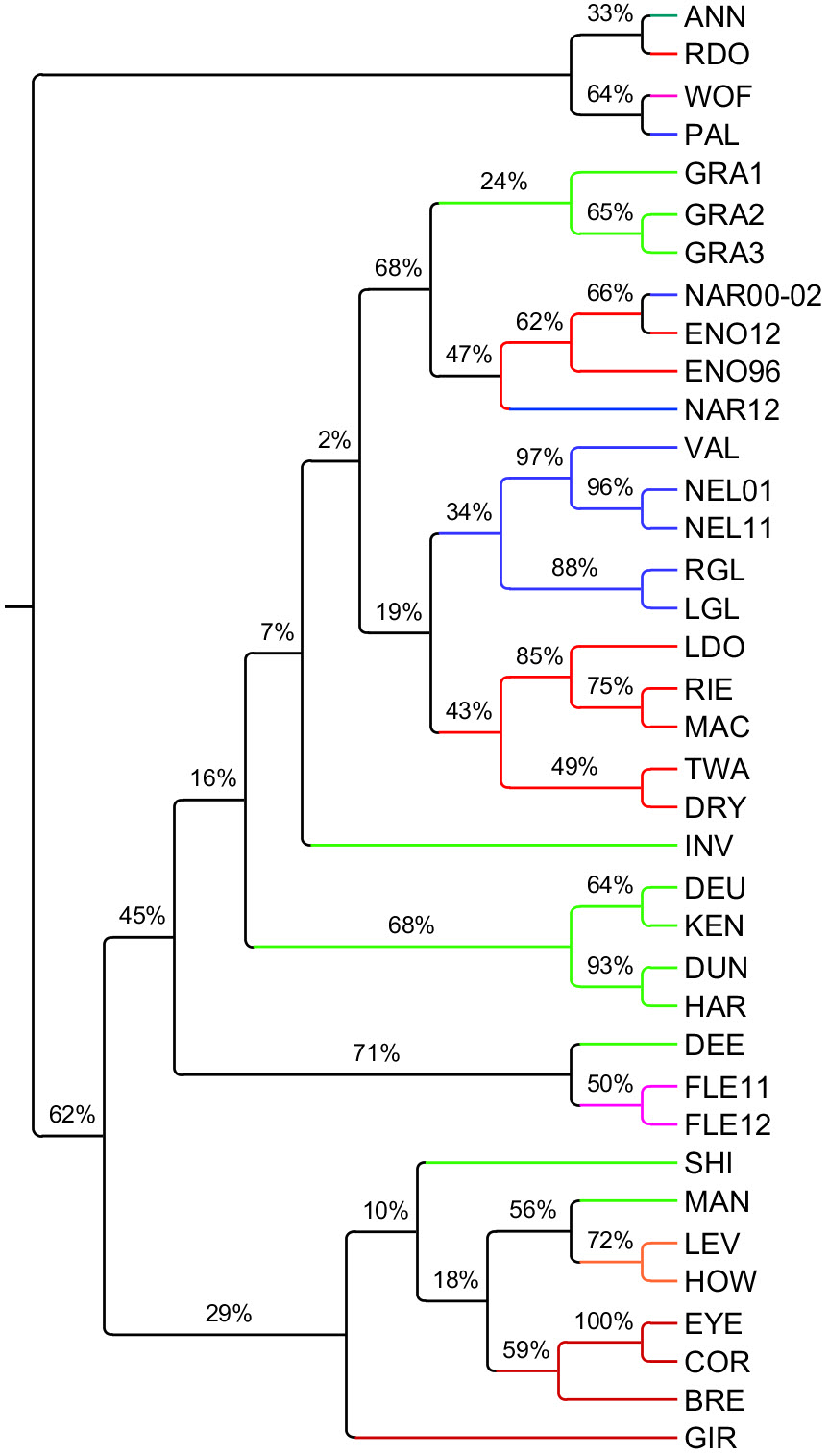

Supplement: Supplementary file 2 — FIGURE S2. Neighbour joining unrooted tree based on Nei's (1983) genetic distance (D A) for Salmo trutta population inferred samples. Percentage bootstrap support shown at nodes. Colours refer to catchment colours as per Figure 1 (except for Loch Leven (LEV) and Howietoun (HOW)). For other sampling site locations, see Table 1. [file JFB-95-719-s002.jpeg]

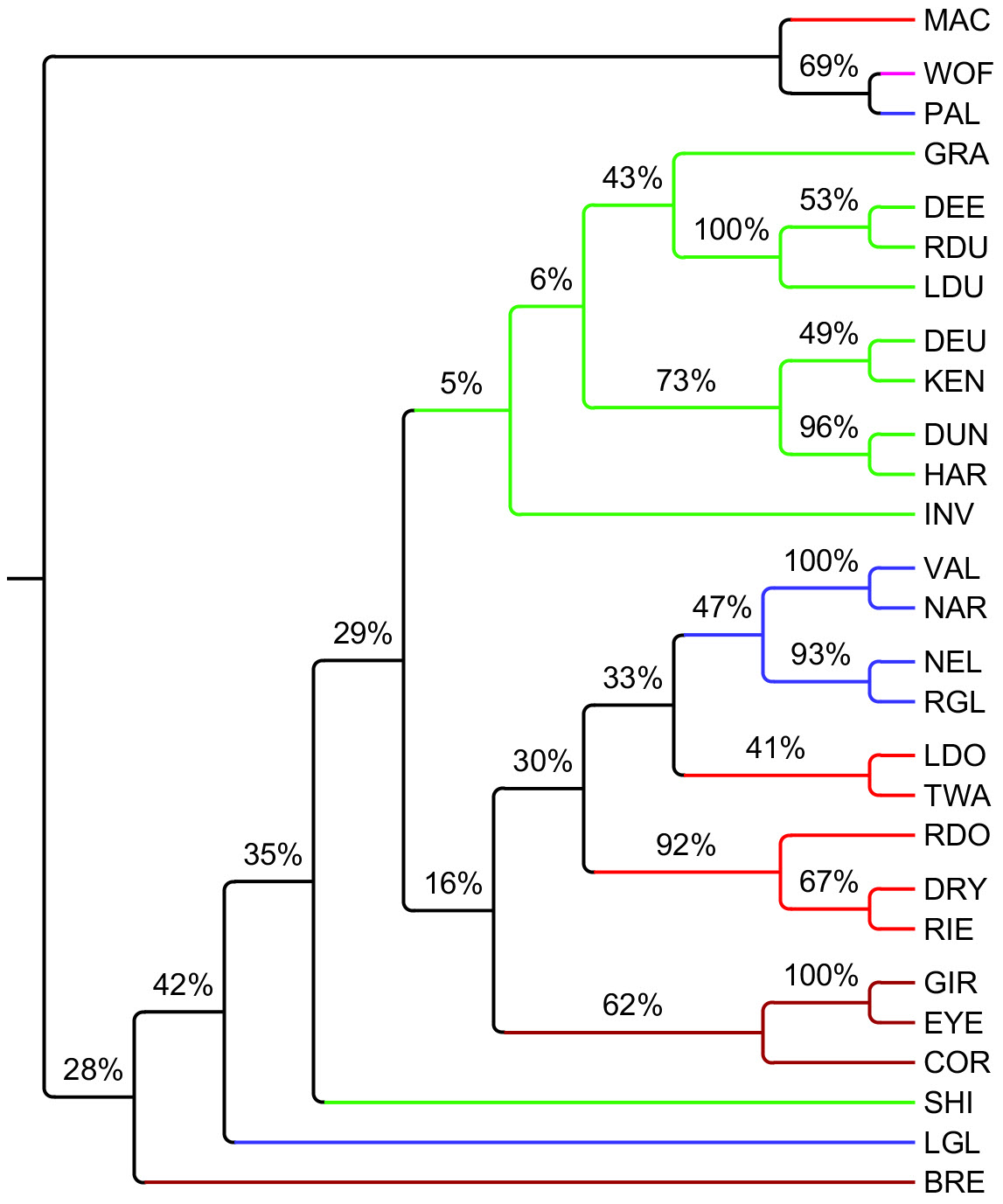

Supplement: Supplementary file 3 — FIGURE S3. Neighbour joining unrooted tree based on Nei's et al. (1983) genetic distance (D A) for Salmo trutta population inferred contemporary samples. Based on natural south‐west Scotland populations only with admixed individuals removed. Percentage bootstrap support shown at nodes. Colours refer to catchment colours as per Figure 1. For other sampling site locations, see Table 1. [file JFB-95-719-s003.jpeg]

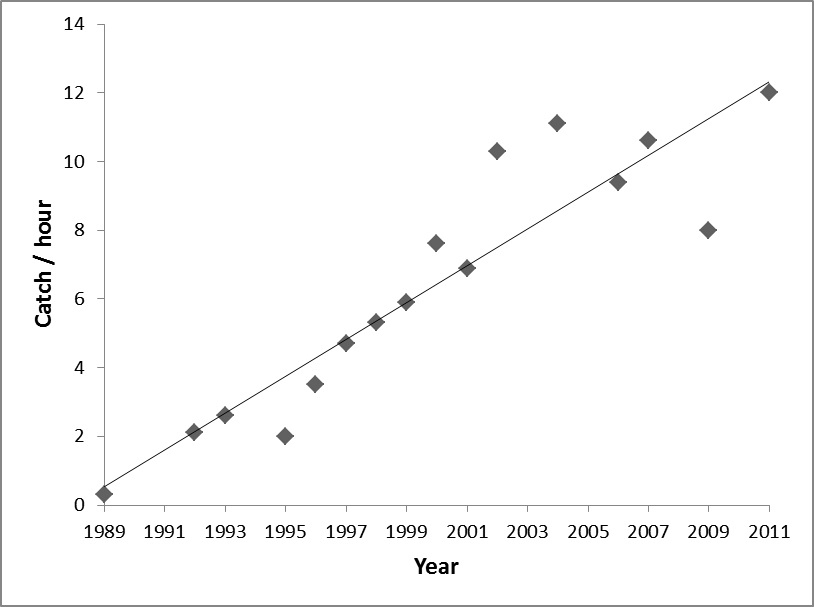

Supplement: Supplementary file 4 — FIGURE S4. Number (n) of Salmo trutta caught per hour by anglers fishing at Round Loch of Glenhead 1989–2011. [file JFB-95-719-s004.jpg]

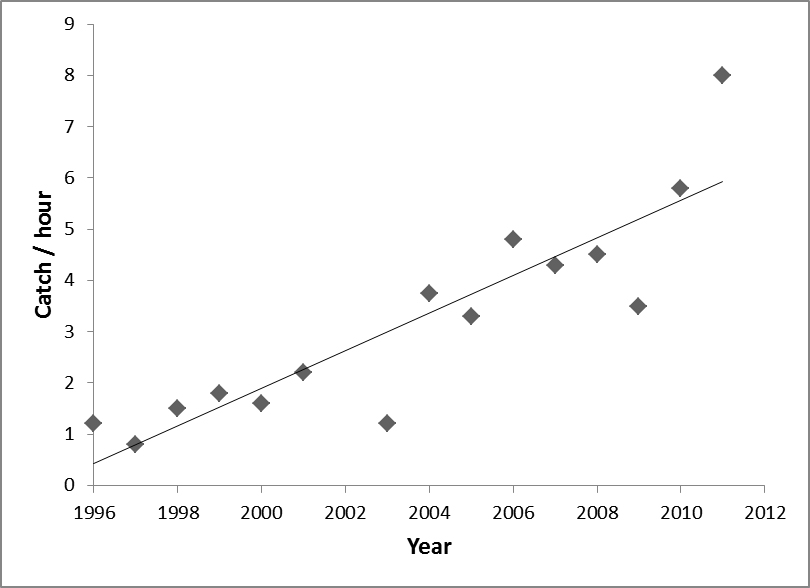

Supplement: Supplementary file 5 — FIGURE S5. Number (n) of Salmo trutta caught per hour by anglers fishing at Loch Valley 1996 to 2011. [file JFB-95-719-s005.jpg]
