# Supplementary material for: Impacts of acidification on brown trout Salmo trutta populations and the contribution of stocking to population recovery and genetic diversity
Source: J Fish Biol. 2019 Jun 24;95(3):719–42. doi: 10.1111/jfb.14054 (PMC6852074; doi:10.1111/jfb.14054)
Supplement: Supplementary file 6 — TABLE S1. Background information on lochs from which Salmo trutta were sampled. [file JFB-95-719-s006.docx]

| **Location** | **Code** | **Altitude**  **(m asl)** | **Area**  **(ha)** | **Catchment**  **(ha)** | **Maximum depth (m)** | **Mean depth (m)** | **Forestation**  **(%)^1^** | **Minimum pH^2^** | **Geology^3^** | **Catch 1978–79^4^** | **Catch 1984^4^** | **CPUE 2010–12^5^** |
| --- | --- | --- | --- | --- | --- | --- | --- | --- | --- | --- | --- | --- |
| Loch Grannoch | GRA | 214 | 115 | 1432 | 21 | 6.4 | 56 | 4.3 | 1 | 28 (2) | 1 (3) | 3.5 |
| Loch Dungeon | DUN | 305 | 40 | 642 | 29 | 6.9 | 5 | 4.6 | 2 | 81 (2) | 33 (3) | 6 |
| Loch Harrow | HAR | 249 | 15 | 385 | 9 | 3.5 | 35 | 4.9 | 2 | na | 4 (3) | 8 |
| Lochinvar | INV | 220 | 51 | 310 | 6 | 1.9 | 30 | na | 2 | na | na | 5 |
| Loch Dee & tributaries | DEE-DEE | 225 | 102 | 1571 | 14 | 4.3 | 16 | 4.7 | 1 | 5 (3) | na | 0.5 |
| Dee–Loch Round Dungeon | DEE-RDU | 270 | 5 | 84 | 6 | 4.9 | 19 | 4.7 | 1 | 6 (2) | 1 (3) | 12 |
| Dee–Loch Long Dungeon | DEE-LDU | 268 | 5 | 240 | 5 | 5.1 | 7 | 4.6 | 1 | 1 (2) | na | 12 |
| Loch Valley | VAL | 326 | 34 | 698 | 17 | 6.3 | 0 | 4.7 | 1 | 0 (2) | 0 (3) | 8 |
| Loch Neldricken | NEL | 348 | 32 | 458 | 15 | 6 | 0 | 4.7 | 1 | 0 (2) | 0 (3) | 5 |
| Loch Round Glenhead | RGL | 298 | 13 | 103 | 14 | 4.3 | 0 | 4.7 | 1 | 0 (2) | 14 (3) | 12 |
| Loch Long Glenhead | LGL | 290 | 10 | 95 | 12 | 4.6 | 0 | na | 1 | 1 (2) | 10 (3) | 7 |
| Loch Eye | EYE | 457 | 2 | 27 | 10 | 5.8 | 0 | 5.8 | 1 | na | na | 19 |
| Loch Cornish | COR | 400 | 5 | 345 | 2 | 1.2 | 0 | 5.8 | 2 | na | na | 40 |
| Loch Brecbowie | BRE | 358 | 8 | 88 | 12 | 5.4 | 7 | 5.6 | 2 | na | na | 9 |
| Loch Doon | LDO | 215 | 820 | 12982 | 31 | 8.1 | 26 | 5.5 | 2 | na | na | 4 |
| Loch Twachtan | TWA | 460 | 0.3 | na | 4 | na | 0 | na | 2 | na | na | 15 |
| Loch Twachtan | DRY | 370 | 3 | 21 | 9 | 5.1 | 15 | 4.6 | 1 | 16 (2) | 11 (3) | 5 |
| Loch Riecawr | RIE | 280 | 90 | 1509 | 9 | 3.7 | 44 | 5.2 | 1 | 21 (1) | na | na |
| Loch Macaterick | MAC | 280 | 80 | 962 | 13 | 4.9 | 13 | 4.6 | 1 | 99 (1) | na | na |
| Loch Fleet | FLE | 338 | 18 | 126 | 17 | 6.2 | 21 | 4.3 | 1 | 0 (2) | 0 (3) | 7 |
| Loch Narroch | NAR | 328 | 3 | 42 | 10 | 5.1 | 0 | 4.5 | 1 | 0 (2) | 0 (3) | 6 |
| Loch Enoch | ENO | 500 | 50 | 222 | 36 | 12.4 | 0 | 4.6 | 1 | 0 (2) | na | 1.5 |
| Loch Mannoch | MAN | 126 | 31 | 2088 | 7 | 3 | 7 | na | 3 | na | na | 6 |

**TABLE S1**Background information on lochs from which *Salmo trutta* were sampled

^1^ Area of coniferous forest in catchment (remaining area primarily acid grassland, bracken and heath; www.eip.ceh.ac.uk/apps/lakes/index.html).

**^2^** Minimum pH 1979–1984 (Harriman *et al*. (1987).

**^3^** 1, Granite and associated rocks; 2, sedimentary Ashgill & Caradoc rocks; 3, sedimentary Llandovery rocks ([www.gateway.snh.gov.uk/pls/apex_cagdb2/f?p=111:1000:::NO](http://www.gateway.snh.gov.uk/pls/apex_cagdb2/f?p=111:1000:::NO):::).

**^4^** Number of trout caught (number of gillnets 24 h^–1^) 1978/79 and 1984 (data from Harriman *et al*., 1987).

**^5^** Maximum angling catch h^–1^ 2010–2012 sampling.

na, data not available.
