# Supplementary material for: Impacts of acidification on brown trout Salmo trutta populations and the contribution of stocking to population recovery and genetic diversity
Source: J Fish Biol. 2019 Jun 24;95(3):719–42. doi: 10.1111/jfb.14054 (PMC6852074; doi:10.1111/jfb.14054)
Supplement: Supplementary file 7 — TABLE S2. Summary population sample statistics per locus and overall loci for Salmo trutta population samples. [file JFB-95-719-s007.docx]

**TABLE S2** Summary population sample statistics per locus and overall loci for *Salmo trutta* population samples

*n*, Number of individuals amplified per locus per population sample for each of the 16 microsatellite loci. *N*_A_, number of alleles per locus per population; *A*%, percentage of total alleles (over all samples) observed; *N*_AR_, allelic richness per locus; *H*_O_, observed heterozygosity per locus; *H*_E_, expected heterozygosity per locus; HWE, Hardy Weinberg Equilibrium; * *P* < 0.05, ** *P* < 0.01, *** *P* < 0.001 (significant deviation). HWE values per locus following Bonferroni correction. Where only one allele is present the relevant statistic is indicated by

Typesetter: highlighted text to appear as foornot below table

|  |  |  |  |  |  |  |  |  |  |  |  |  |  |  |  |  |  |
| --- | --- | --- | --- | --- | --- | --- | --- | --- | --- | --- | --- | --- | --- | --- | --- | --- | --- |
| **Location,code & parameters** | **Microsatellite markers** | | | | | | | | | | | | | | | | |
|  | **Ssa85** | **One102-a** | **One102-b** | **CA054565** | **Ssa416** | **One103** | **Cocl-Lav-4** | **One9uASC** | **CA048828** | **BG935488** | **SsaD71** | **SaSaTAP2A** | **Ssa410UOS** | **ppStr3** | **CA060177** | **Ssa197** | **Overall** |
| Loch Grannoch | | | | | | | | | | | | | | | | | |
| GRA_82_ |  |  |  |  |  |  |  |  |  |  |  |  |  |  |  |  |  |
| *n* | 23 | 23 | 22 | 23 | 23 | 23 | 23 | 23 | 23 | 22 | 23 | 22 | 22 | 23 | 23 | 22 | 22.69 |
| *N*_A_ | 4 | 2 | 13 | 1 | 2 | 4 | 4 | 4 | 11 | 4 | 6 | 5 | 12 | 3 | 5 | 5 | 85 |
| A% | 40 | 100 | 56.52 | 20 | 50 | 50 | 36.36 | 30.77 | 26.19 | 36.36 | 33.33 | 31.25 | 30.77 | 27.27 | 27.78 | 33.33 | 39.37 |
| *N*_AR_ | 4 | 2 | 13 | 1 | 2 | 4 | 4 | 4 | 11 | 4 | 6 | 5 | 12 | 3 | 5 | 5 | 5.31 |
| *H*_O_ | 0.83 | 0.43 | 1 | - | 0.04 | 0.61 | 0.83 | 0.48 | 1 | 0.77 | 0.65 | 0.77 | 0.91 | 0.83 | 0.74 | 0.68 | 0.66 |
| *H*_E_ | 0.71 | 0.49 | 0.9 | - | 0.04 | 0.63 | 0.61 | 0.69 | 0.88 | 0.74 | 0.77 | 0.62 | 0.81 | 0.59 | 0.66 | 0.65 | 0.61 |
| HWE | ns | ns | ns | - | ns | ns | ns | ns | ns | ns | ** | ns | ns | ns | *** | ns | * |
| GRA_94, 02_ |  |  |  |  |  |  |  |  |  |  |  |  |  |  |  |  |  |
| *n* | 67 | 67 | 67 | 67 | 67 | 67 | 66 | 64 | 65 | 67 | 66 | 61 | 66 | 66 | 64 | 67 | 65.88 |
| *N*_A_ | 4 | 2 | 15 | 1 | 2 | 5 | 4 | 4 | 12 | 4 | 7 | 4 | 15 | 4 | 6 | 5 | 94 |
| A% | 40 | 100 | 65.22 | 20 | 50 | 62.5 | 36.36 | 30.77 | 28.57 | 36.36 | 38.89 | 25 | 38.46 | 36.36 | 33.33 | 33.33 | 42.2 |
| *N*_AR_ | 4.00 | 2.00 | 13.17 | 1.00 | 1.99 | 4.61 | 4.00 | 3.99 | 10.54 | 3.95 | 6.80 | 3.46 | 12.56 | 4.00 | 5.81 | 4.82 | 5.43 |
| *H*_O_ | 0.72 | 0.58 | 0.91 | - | 0.12 | 0.69 | 0.56 | 0.64 | 0.86 | 0.67 | 0.79 | 0.43 | 0.89 | 0.59 | 0.72 | 0.73 | 0.62 |
| *H*_E_ | 0.71 | 0.5 | 0.89 | - | 0.11 | 0.69 | 0.64 | 0.67 | 0.84 | 0.63 | 0.78 | 0.45 | 0.86 | 0.56 | 0.71 | 0.75 | 0.61 |
| HWE | ns | ns | ns | - | ns | * | * | ns | ns | ns | ns | ns | ns | ns | ns | ns | ns |
| GRA_10-12_ |  |  |  |  |  |  |  |  |  |  |  |  |  |  |  |  |  |
| *n* | 266 | 266 | 263 | 266 | 266 | 263 | 251 | 264 | 263 | 255 | 264 | 262 | 264 | 264 | 263 | 265 | 262.81 |
| *N*_A_ | 5 | 2 | 16 | 1 | 2 | 6 | 6 | 4 | 15 | 5 | 8 | 6 | 17 | 5 | 7 | 6 | 111 |
| A% | 50 | 100 | 69.57 | 20 | 50 | 75 | 54.55 | 30.77 | 35.71 | 45.45 | 44.44 | 37.5 | 43.59 | 45.45 | 38.89 | 40 | 48.81 |
| *N*_AR_ | 4.11 | 2.00 | 13.49 | 1.00 | 1.99 | 5.51 | 5.69 | 3.98 | 12.83 | 4.12 | 6.78 | 4.01 | 12.63 | 4.30 | 6.65 | 5.37 | 5.9 |
| *H*_O_ | 0.7 | 0.52 | 0.88 | - | 0.14 | 0.74 | 0.56 | 0.64 | 0.87 | 0.47 | 0.78 | 0.55 | 0.88 | 0.49 | 0.75 | 0.79 | 0.61 |
| *H*_E_ | 0.72 | 0.5 | 0.89 | - | 0.15 | 0.74 | 0.72 | 0.63 | 0.86 | 0.71 | 0.78 | 0.52 | 0.84 | 0.48 | 0.76 | 0.76 | 0.63 |
| HWE | ns | ns | ns | - | ns | ns | *** | ns | ns | *** | ns | ns | ns | ns | ns | ns | *** |
| River Deuch | | | | | | | | | | | | | | | | | |
| DEU |  |  |  |  |  |  |  |  |  |  |  |  |  |  |  |  |  |
| *n* | 66 | 77 | 77 | 76 | 77 | 76 | 63 | 76 | 77 | 77 | 77 | 75 | 77 | 77 | 77 | 77 | 75.12 |
| *N*_A_ | 3 | 2 | 13 | 2 | 3 | 6 | 6 | 4 | 23 | 8 | 8 | 7 | 22 | 4 | 13 | 6 | 130 |
| A% | 30 | 100 | 56.52 | 40 | 75 | 75 | 54.55 | 30.77 | 54.76 | 72.73 | 44.44 | 43.75 | 56.41 | 36.36 | 72.22 | 40 | 55.16 |
| *N*_AR_ | 3.00 | 2.00 | 11.48 | 1.95 | 2.17 | 5.38 | 5.20 | 3.39 | 16.31 | 7.11 | 7.67 | 5.80 | 17.58 | 3.90 | 11.44 | 5.16 | 6.85 |
| *H*_O_ | 0.7 | 0.45 | 0.9 | 0.05 | 0.05 | 0.63 | 0.62 | 0.38 | 0.78 | 0.69 | 0.66 | 0.79 | 0.84 | 0.51 | 0.77 | 0.75 | 0.6 |
| *H*_E_ | 0.66 | 0.5 | 0.86 | 0.08 | 0.05 | 0.71 | 0.65 | 0.37 | 0.75 | 0.73 | 0.73 | 0.79 | 0.93 | 0.55 | 0.75 | 0.72 | 0.61 |
| HWE | ns | ns | ns | * | ns | ns | ns | * | ns | ** | ns | ns | ns | ns | ns | ns | ns |
| River Ken | | | | | | | | | | | | | | | | | |
| KEN |  |  |  |  |  |  |  |  |  |  |  |  |  |  |  |  |  |
| *n* | 61 | 61 | 61 | 60 | 60 | 61 | 59 | 61 | 61 | 61 | 60 | 57 | 61 | 61 | 60 | 61 | 60.38 |
| *N*_A_ | 4 | 2 | 11 | 2 | 2 | 5 | 6 | 5 | 16 | 8 | 10 | 8 | 20 | 4 | 8 | 6 | 117 |
| A% | 40 | 100 | 47.83 | 40 | 50 | 62.5 | 54.55 | 38.46 | 38.1 | 72.73 | 55.56 | 50 | 51.28 | 36.36 | 44.44 | 40 | 51.36 |
| *N*_AR_ | 3.93 | 2.00 | 10.44 | 1.50 | 1.94 | 4.43 | 5.46 | 4.81 | 11.78 | 7.10 | 8.26 | 7.49 | 17.95 | 3.98 | 6.70 | 5.87 | 6.48 |
| *H*_O_ | 0.46 | 0.31 | 0.75 | 0.02 | 0.03 | 0.43 | 0.61 | 0.43 | 0.67 | 0.85 | 0.63 | 0.67 | 0.9 | 0.44 | 0.55 | 0.62 | 0.52 |
| *H*_E_ | 0.57 | 0.39 | 0.87 | 0.02 | 0.06 | 0.5 | 0.68 | 0.45 | 0.7 | 0.78 | 0.73 | 0.77 | 0.93 | 0.44 | 0.65 | 0.74 | 0.58 |
| HWE | ns | ns | *** | ns | *** | * | ns | ns | *** | ns | ns | ns | ns | ns | ns | ns | *** |
| Loch Dungeon | | | | | | | | | | | | | | | | | |
| DUN |  |  |  |  |  |  |  |  |  |  |  |  |  |  |  |  |  |
| *n* | 34 | 79 | 79 | 77 | 79 | 79 | 34 | 78 | 79 | 79 | 79 | 79 | 79 | 79 | 79 | 79 | 73.19 |
| *N*_A_ | 1 | 2 | 9 | 1 | 2 | 4 | 1 | 6 | 15 | 4 | 6 | 5 | 13 | 3 | 6 | 5 | 83 |
| A% | 10 | 100 | 39.13 | 20 | 50 | 50 | 9.09 | 46.15 | 35.71 | 36.36 | 33.33 | 31.25 | 33.33 | 27.27 | 33.33 | 33.33 | 36.77 |
| *N*_AR_ | 1.00 | 1.95 | 8.21 | 1.00 | 1.86 | 3.95 | 1.00 | 4.87 | 13.00 | 3.85 | 5.88 | 4.62 | 11.75 | 2.62 | 5.73 | 4.86 | 4.76 |
| *H*_O_ | 0 | 0.08 | 0.77 | - | 0.05 | 0.63 | - | 0.65 | 0.85 | 0.48 | 0.48 | 0.71 | 0.87 | 0.25 | 0.67 | 0.71 | 0.45 |
| *H*_E_ | 0 | 0.07 | 0.78 | - | 0.05 | 0.62 | - | 0.65 | 0.85 | 0.47 | 0.49 | 0.7 | 0.88 | 0.22 | 0.59 | 0.73 | 0.44 |
| HWE | - | ns | ns | - | ns | ns | - | ns | ns | ns | ns | ns | ns | ns | ns | * | ns |
| Loch Harrow | | | | | | | | | | | | | | | | | |
| HAR |  |  |  |  |  |  |  |  |  |  |  |  |  |  |  |  |  |
| *n* | 91 | 91 | 90 | 91 | 90 | 90 | 90 | 89 | 90 | 90 | 87 | 85 | 84 | 91 | 89 | 91 | 89.31 |
| *N*_A_ | 4 | 2 | 14 | 2 | 2 | 6 | 5 | 6 | 13 | 4 | 10 | 6 | 21 | 5 | 9 | 4 | 113 |
| A% | 40 | 100 | 60.87 | 40 | 50 | 75 | 45.45 | 46.15 | 30.95 | 36.36 | 55.56 | 37.5 | 53.85 | 45.45 | 50 | 26.67 | 49.61 |
| *N*_AR_ | 3.70 | 2.00 | 11.19 | 1.55 | 1.33 | 5.91 | 4.61 | 4.65 | 10.20 | 4.00 | 7.63 | 5.97 | 16.25 | 4.24 | 5.47 | 4.00 | 5.79 |
| *H*_O_ | 0.32 | 0.34 | 0.83 | 0.02 | 0.01 | 0.73 | 0.4 | 0.6 | 0.91 | 0.71 | 0.61 | 0.66 | 0.98 | 0.37 | 0.66 | 0.76 | 0.56 |
| *H*_E_ | 0.37 | 0.47 | 0.84 | 0.02 | 0.01 | 0.75 | 0.41 | 0.61 | 0.84 | 0.72 | 0.71 | 0.73 | 0.92 | 0.37 | 0.66 | 0.69 | 0.57 |
| HWE | *** | * | *** | ns | ns | ns | ns | ns | ns | ns | ns | ns | ns | *** | *** | ns | *** |
| Lochinvar |  |  |  |  |  |  |  |  |  |  |  |  |  |  |  |  |  |
| INV |  |  |  |  |  |  |  |  |  |  |  |  |  |  |  |  |  |
| *n* | 38 | 38 | 38 | 38 | 38 | 38 | 38 | 38 | 38 | 38 | 37 | 36 | 37 | 38 | 36 | 38 | 37.62 |
| *N*_A_ | 4 | 2 | 8 | 1 | 1 | 4 | 4 | 4 | 7 | 4 | 5 | 4 | 8 | 3 | 6 | 4 | 69 |
| A% | 40 | 100 | 34.78 | 20 | 25 | 50 | 36.36 | 30.77 | 16.67 | 36.36 | 27.78 | 25 | 20.51 | 27.27 | 33.33 | 26.67 | 34.41 |
| *N*_AR_ | 4.00 | 2.00 | 7.78 | 1.00 | 1.00 | 4.00 | 3.99 | 4.00 | 6.99 | 3.99 | 4.97 | 4.00 | 7.77 | 2.99 | 6.00 | 4.00 | 4.28 |
| *H*_O_ | 0.63 | 0.45 | 0.84 | - | - | 0.71 | 0.66 | 0.71 | 0.74 | 0.45 | 0.76 | 0.64 | 0.92 | 0.45 | 0.81 | 0.63 | 0.59 |
| *H*_E_ | 0.65 | 0.49 | 0.78 | - | - | 0.66 | 0.69 | 0.59 | 0.74 | 0.47 | 0.71 | 0.59 | 0.82 | 0.48 | 0.81 | 0.69 | 0.57 |
| HWE | ns | ns | ns | - | - | ns | ns | ns | ns | ns | ns | ns | ns | ns | ns | ns | ns |
| Loch Dee & tributaries | | | | | | | | | | | | | | | | | |
| DEE |  |  |  |  |  |  |  |  |  |  |  |  |  |  |  |  |  |
| *n* | 202 | 202 | 201 | 202 | 202 | 202 | 198 | 202 | 201 | 202 | 201 | 201 | 201 | 202 | 195 | 202 | 201 |
| *N*_A_ | 4 | 2 | 16 | 2 | 4 | 7 | 7 | 8 | 20 | 10 | 12 | 7 | 23 | 6 | 12 | 7 | 147 |
| A% | 40 | 100 | 69.57 | 40 | 100 | 87.5 | 63.64 | 61.54 | 47.62 | 90.91 | 66.67 | 43.75 | 58.97 | 54.55 | 66.67 | 46.67 | 64.88 |
| *N*_AR_ | 3.67 | 2.00 | 13.34 | 1.83 | 3.14 | 6.35 | 5.34 | 6.11 | 13.81 | 6.86 | 7.93 | 6.42 | 16.64 | 4.94 | 9.29 | 6.47 | 7.13 |
| *H*_O_ | 0.48 | 0.44 | 0.88 | 0.05 | 0.41 | 0.53 | 0.42 | 0.74 | 0.81 | 0.66 | 0.82 | 0.71 | 0.92 | 0.53 | 0.86 | 0.68 | 0.62 |
| *H*_E_ | 0.46 | 0.43 | 0.91 | 0.05 | 0.41 | 0.61 | 0.61 | 0.75 | 0.85 | 0.76 | 0.8 | 0.77 | 0.92 | 0.59 | 0.86 | 0.72 | 0.66 |
| HWE | ns | ns | ns | ns | ns | ns | *** | ns | ns | *** | ns | * | ns | ns | ns | ns | * |
| River Shirmers | | | | | | | | | | | | | | | | | |
| SHI |  |  |  |  |  |  |  |  |  |  |  |  |  |  |  |  |  |
| *n* | 68 | 68 | 68 | 68 | 68 | 68 | 68 | 68 | 68 | 67 | 68 | 68 | 68 | 68 | 68 | 68 | 67.94 |
| *N*_A_ | 6 | 2 | 13 | 3 | 3 | 7 | 7 | 8 | 21 | 7 | 12 | 6 | 17 | 6 | 10 | 7 | 135 |
| A% | 60 | 100 | 56.52 | 60 | 75 | 87.5 | 63.64 | 61.54 | 50 | 63.64 | 66.67 | 37.5 | 43.59 | 54.55 | 55.56 | 46.67 | 61.4 |
| *N*_AR_ | 5.44 | 2.00 | 11.17 | 1.88 | 2.95 | 5.32 | 6.43 | 6.85 | 18.01 | 6.81 | 11.49 | 5.52 | 13.73 | 5.41 | 9.25 | 6.31 | 7.41 |
| *H*_O_ | 0.71 | 0.41 | 0.84 | 0.03 | 0.24 | 0.65 | 0.78 | 0.76 | 0.93 | 0.67 | 0.91 | 0.53 | 0.84 | 0.5 | 0.84 | 0.62 | 0.64 |
| *H*_E_ | 0.72 | 0.48 | 0.84 | 0.03 | 0.25 | 0.61 | 0.77 | 0.76 | 0.92 | 0.74 | 0.87 | 0.62 | 0.89 | 0.63 | 0.85 | 0.66 | 0.67 |
| HWE | ns | ns | ns | ns | ns | ns | ns | ns | ns | * | ns | ns | ns | ns | ns | ns | ns |
| Loch Mannoch | | | | | | | | | | | | | | | | | |
| MAN |  |  |  |  |  |  |  |  |  |  |  |  |  |  |  |  |  |
| *n* | 31 | 31 | 31 | 30 | 31 | 31 | 26 | 31 | 29 | 31 | 29 | 30 | 31 | 31 | 31 | 31 | 30.31 |
| *N*_A_ | 4 | 2 | 14 | 2 | 3 | 6 | 5 | 7 | 17 | 8 | 12 | 7 | 20 | 3 | 9 | 7 | 126 |
| A% | 40 | 100 | 60.87 | 40 | 75 | 75 | 45.45 | 53.85 | 40.48 | 72.73 | 66.67 | 43.75 | 51.28 | 27.27 | 50 | 46.67 | 55.56 |
| *N*_AR_ | 4.00 | 2.00 | 13.97 | 2.00 | 3.00 | 6.00 | 5.00 | 6.97 | 17.00 | 7.90 | 12.00 | 7.00 | 19.80 | 3.00 | 8.97 | 6.94 | 7.85 |
| *H*_O_ | 0.71 | 0.39 | 0.84 | 0.03 | 0.13 | 0.68 | 0.65 | 0.68 | 0.76 | 0.61 | 0.83 | 0.73 | 1 | 0.35 | 0.77 | 0.74 | 0.62 |
| *H*_E_ | 0.69 | 0.35 | 0.88 | 0.03 | 0.23 | 0.73 | 0.69 | 0.69 | 0.87 | 0.53 | 0.82 | 0.8 | 0.93 | 0.43 | 0.8 | 0.74 | 0.64 |
| HWE | ns | ns | * | ns | *** | ns | ns | ns | ns | ns | ** | ns | ns | ns | ns | ns | ** |
| Loch Fleet |  |  |  |  |  |  |  |  |  |  |  |  |  |  |  |  |  |
| FLE_11_ |  |  |  |  |  |  |  |  |  |  |  |  |  |  |  |  |  |
| *n* | 166 | 167 | 167 | 167 | 166 | 166 | 165 | 167 | 167 | 164 | 165 | 166 | 167 | 167 | 166 | 166 | 166.19 |
| *N*_A_ | 4 | 2 | 17 | 2 | 4 | 7 | 7 | 7 | 19 | 4 | 9 | 7 | 23 | 5 | 10 | 10 | 137 |
| A% | 40 | 100 | 73.91 | 40 | 100 | 87.5 | 63.64 | 53.85 | 45.24 | 36.36 | 50 | 43.75 | 58.97 | 45.45 | 55.56 | 66.67 | 60.06 |
| *N*_AR_ | 4.00 | 2.00 | 12.38 | 1.70 | 2.73 | 5.50 | 6.17 | 5.88 | 14.99 | 4.00 | 7.10 | 6.26 | 19.02 | 4.87 | 8.39 | 8.00 | 7.06 |
| *H*_O_ | 0.64 | 0.45 | 0.88 | 0.04 | 0.19 | 0.7 | 0.61 | 0.7 | 0.86 | 0.64 | 0.72 | 0.77 | 0.93 | 0.57 | 0.84 | 0.73 | 0.64 |
| *H*_E_ | 0.66 | 0.47 | 0.87 | 0.04 | 0.21 | 0.66 | 0.67 | 0.74 | 0.88 | 0.74 | 0.74 | 0.76 | 0.94 | 0.58 | 0.85 | 0.78 | 0.66 |
| HWE | ns | ns | ns | ns | ns | ns | ** | ns | *** | ** | ns | ns | *** | ns | ns | ns | *** |
| FLE_12_ |  |  |  |  |  |  |  |  |  |  |  |  |  |  |  |  |  |
| *n* | 72 | 72 | 71 | 72 | 72 | 72 | 71 | 72 | 68 | 71 | 72 | 72 | 72 | 72 | 71 | 72 | 71.5 |
| *N*_A_ | 4 | 2 | 17 | 2 | 4 | 7 | 8 | 5 | 18 | 5 | 7 | 7 | 20 | 5 | 8 | 9 | 128 |
| A% | 40 | 100 | 73.91 | 40 | 100 | 87.5 | 72.73 | 38.46 | 42.86 | 45.45 | 38.89 | 43.75 | 51.28 | 45.45 | 44.44 | 60 | 57.8 |
| *N*_AR_ | 4.00 | 2.00 | 13.59 | 1.98 | 3.32 | 5.25 | 7.43 | 4.94 | 15.90 | 4.42 | 6.04 | 6.61 | 17.20 | 4.60 | 7.82 | 8.00 | 7.07 |
| *H*_O_ | 0.6 | 0.58 | 0.87 | 0.1 | 0.31 | 0.62 | 0.62 | 0.62 | 0.88 | 0.66 | 0.88 | 0.75 | 0.89 | 0.5 | 0.7 | 0.78 | 0.65 |
| *H*_E_ | 0.61 | 0.49 | 0.87 | 0.09 | 0.29 | 0.67 | 0.72 | 0.76 | 0.9 | 0.72 | 0.74 | 0.72 | 0.92 | 0.51 | 0.78 | 0.8 | 0.66 |
| HWE | ns | ns | ns | ns | ns | ns | ns | * | ns | ns | ns | ns | * | ns | ns | *** | * |
| River Water of Fleet | | | | | | | | | | | | | | | | | |
| WOF |  |  |  |  |  |  |  |  |  |  |  |  |  |  |  |  |  |
| *n* | 105 | 105 | 104 | 105 | 105 | 105 | 102 | 104 | 103 | 105 | 105 | 104 | 104 | 105 | 105 | 105 | 104.44 |
| *N*_A_ | 5 | 2 | 17 | 4 | 3 | 6 | 7 | 7 | 27 | 9 | 12 | 9 | 30 | 6 | 12 | 8 | 164 |
| A% | 50 | 100 | 73.91 | 80 | 75 | 75 | 63.64 | 53.85 | 64.29 | 81.82 | 66.67 | 56.25 | 76.92 | 54.55 | 66.67 | 53.33 | 68.24 |
| *N*_AR_ | 4.80 | 2.00 | 14.16 | 3.36 | 3.00 | 5.45 | 5.94 | 5.76 | 20.49 | 8.05 | 10.60 | 7.31 | 23.35 | 5.47 | 11.48 | 6.21 | 8.59 |
| *H*_O_ | 0.68 | 0.36 | 0.86 | 0.29 | 0.46 | 0.67 | 0.66 | 0.75 | 0.9 | 0.71 | 0.8 | 0.78 | 0.92 | 0.67 | 0.8 | 0.72 | 0.69 |
| *H*_E_ | 0.67 | 0.41 | 0.9 | 0.28 | 0.51 | 0.69 | 0.7 | 0.68 | 0.93 | 0.81 | 0.85 | 0.82 | 0.95 | 0.67 | 0.86 | 0.77 | 0.72 |
| HWE | ns | ns | *** | ns | ns | ns | ns | ns | * | ns | ns | * | ** | ns | ns | ns | *** |
| River Palnure | | | | | | | | | | | | | | | | | |
| PAL |  |  |  |  |  |  |  |  |  |  |  |  |  |  |  |  |  |
| *n* | 51 | 51 | 51 | 51 | 51 | 51 | 44 | 50 | 51 | 51 | 49 | 51 | 50 | 51 | 50 | 51 | 50.25 |
| *N*_A_ | 7 | 2 | 17 | 4 | 2 | 7 | 6 | 7 | 23 | 10 | 11 | 7 | 22 | 4 | 12 | 9 | 150 |
| A% | 70 | 100 | 73.91 | 80 | 50 | 87.5 | 54.55 | 53.85 | 54.76 | 90.91 | 61.11 | 43.75 | 56.41 | 36.36 | 66.67 | 60 | 64.99 |
| *N*_AR_ | 6.15 | 2.00 | 15.90 | 3.42 | 2.00 | 6.67 | 5.87 | 6.04 | 21.29 | 9.04 | 10.10 | 6.56 | 19.54 | 4.00 | 11.66 | 8.32 | 8.66 |
| *H*_O_ | 0.57 | 0.39 | 1 | 0.2 | 0.16 | 0.8 | 0.64 | 0.68 | 0.92 | 0.78 | 0.76 | 0.8 | 0.96 | 0.67 | 0.78 | 0.75 | 0.68 |
| *H*_E_ | 0.67 | 0.43 | 0.91 | 0.2 | 0.18 | 0.76 | 0.61 | 0.73 | 0.95 | 0.81 | 0.81 | 0.8 | 0.93 | 0.62 | 0.88 | 0.79 | 0.69 |
| HWE | ns | ns | ns | ns | ns | ns | ns | ns | ns | ns | ns | ns | * | ns | ns | ns | * |
| Loch Valley | | | | | | | | | | | | | | | | | |
| VAL |  |  |  |  |  |  |  |  |  |  |  |  |  |  |  |  |  |
| *n* | 50 | 51 | 51 | 51 | 50 | 51 | 51 | 51 | 51 | 49 | 50 | 51 | 51 | 51 | 50 | 51 | 50.62 |
| *N*_A_ | 4 | 2 | 8 | 1 | 2 | 5 | 5 | 4 | 8 | 4 | 4 | 3 | 9 | 4 | 3 | 4 | 70 |
| A% | 40 | 100 | 34.78 | 20 | 50 | 62.5 | 45.45 | 30.77 | 19.05 | 36.36 | 22.22 | 18.75 | 23.08 | 36.36 | 16.67 | 26.67 | 36.42 |
| *N*_AR_ | 3.98 | 2.00 | 7.00 | 1.00 | 2.00 | 4.93 | 4.59 | 3.83 | 7.19 | 4.00 | 3.94 | 3.00 | 8.11 | 3.95 | 3.00 | 3.83 | 4.15 |
| *H*_O_ | 0.4 | 0.53 | 0.73 | - | 0.12 | 0.63 | 0.51 | 0.49 | 0.71 | 0.24 | 0.5 | 0.57 | 0.88 | 0.24 | 0.36 | 0.49 | 0.46 |
| *H*_E_ | 0.44 | 0.46 | 0.7 | - | 0.11 | 0.54 | 0.62 | 0.48 | 0.65 | 0.66 | 0.47 | 0.57 | 0.81 | 0.25 | 0.31 | 0.44 | 0.47 |
| HWE | ns | ns | ns | - | ns | ns | ns | ns | ns | *** | ns | ns | ns | ns | ns | ns | ns |
| Loch Narroch | | | | | | | | | | | | | | | | | |
| NAR_00-02_ |  |  |  |  |  |  |  |  |  |  |  |  |  |  |  |  |  |
| *n* | 52 | 52 | 52 | 52 | 52 | 52 | 52 | 52 | 52 | 50 | 52 | 52 | 51 | 52 | 51 | 52 | 51.75 |
| *N*_A_ | 4 | 2 | 8 | 1 | 2 | 4 | 4 | 4 | 6 | 4 | 6 | 2 | 7 | 4 | 3 | 5 | 66 |
| A% | 40 | 100 | 34.78 | 20 | 50 | 50 | 36.36 | 30.77 | 14.29 | 36.36 | 33.33 | 12.5 | 17.95 | 36.36 | 16.67 | 33.33 | 35.17 |
| *N*_AR_ | 4.00 | 2.00 | 7.56 | 1.00 | 2.00 | 3.97 | 4.00 | 4.00 | 5.97 | 4.00 | 5.99 | 2.00 | 6.41 | 3.58 | 3.00 | 4.58 | 4 |
| *H*_O_ | 0.71 | 0.4 | 0.92 | - | 0.35 | 0.77 | 0.71 | 0.71 | 0.92 | 0.62 | 0.87 | 0.42 | 0.8 | 0.5 | 0.82 | 0.92 | 0.65 |
| *H*_E_ | 0.72 | 0.32 | 0.79 | - | 0.29 | 0.67 | 0.71 | 0.7 | 0.73 | 0.59 | 0.76 | 0.44 | 0.74 | 0.4 | 0.66 | 0.72 | 0.58 |
| HWE | ns | ns | ns | - | ns | ns | ns | ns | ns | ns | ns | ns | ns | ns | * | * | ns |
| NAR_12_ |  |  |  |  |  |  |  |  |  |  |  |  |  |  |  |  |  |
| *n* | 13 | 13 | 13 | 13 | 13 | 13 | 13 | 11 | 11 | 12 | 13 | 13 | 13 | 13 | 13 | 13 | 12.69 |
| *N*_A_ | 4 | 2 | 7 | 1 | 2 | 5 | 5 | 4 | 6 | 4 | 6 | 3 | 5 | 2 | 3 | 4 | 63 |
| A% | 40 | 100 | 30.43 | 20 | 50 | 62.5 | 45.45 | 30.77 | 14.29 | 36.36 | 33.33 | 18.75 | 12.82 | 18.18 | 16.67 | 26.67 | 34.76 |
| *N*_AR_ | 4 | 2 | 7 | 1 | 2 | 5 | 5 | 4 | 6 | 4 | 6 | 3 | 5 | 2 | 3 | 4 | 3.94 |
| *H*_O_ | 0.62 | 0.46 | 0.62 | - | 0.15 | 0.85 | 0.92 | 0.55 | 0.73 | 0.33 | 0.46 | 0.62 | 0.77 | 0.23 | 0.62 | 0.62 | 0.53 |
| *H*_E_ | 0.7 | 0.5 | 0.81 | - | 0.14 | 0.75 | 0.74 | 0.71 | 0.75 | 0.61 | 0.54 | 0.57 | 0.72 | 0.2 | 0.55 | 0.68 | 0.56 |
| HWE | ns | ns | ns | - | ns | ns | ns | ns | ns | ns | ns | ns | ns | ns | ns | ns | ns |
| Loch Neldricken | | | | | | | | | | | | | | | | | |
| NEL_01_ |  |  |  |  |  |  |  |  |  |  |  |  |  |  |  |  |  |
| *n* | 23 | 23 | 23 | 23 | 23 | 23 | 23 | 23 | 23 | 23 | 23 | 23 | 23 | 23 | 23 | 23 | 23 |
| *N*_A_ | 1 | 1 | 3 | 1 | 1 | 1 | 2 | 1 | 2 | 2 | 1 | 2 | 4 | 2 | 1 | 1 | 26 |
| A% | 10 | 50 | 13.04 | 20 | 25 | 12.5 | 18.18 | 7.69 | 4.76 | 18.18 | 5.56 | 12.5 | 10.26 | 18.18 | 5.56 | 6.67 | 14.88 |
| *N*_AR_ | 1 | 1 | 3 | 1 | 1 | 1 | 2 | 1 | 2 | 2 | 1 | 2 | 4 | 2 | 1 | 1 | 1.63 |
| *H*_O_ | 0 | 0 | 0.65 | - | - | - | 0.04 | - | 0.04 | 0.09 | - | 0.13 | 0.74 | 0.26 | - | - | 0.12 |
| *H*_E_ | 0 | 0 | 0.52 | - | - | - | 0.04 | - | 0.04 | 0.23 | - | 0.12 | 0.7 | 0.29 | - | - | 0.12 |
| HWE | - | - | ns | - | - | - | ns | - | ns | ** | - | ns | ns | ns | - | - | ns |
| NEL_11_ |  |  |  |  |  |  |  |  |  |  |  |  |  |  |  |  |  |
| *n* | 53 | 53 | 53 | 53 | 52 | 53 | 53 | 53 | 53 | 50 | 51 | 52 | 50 | 53 | 49 | 53 | 52.12 |
| *N*_A_ | 4 | 2 | 8 | 1 | 2 | 4 | 5 | 4 | 8 | 6 | 4 | 4 | 12 | 4 | 4 | 5 | 77 |
| A% | 40 | 100 | 34.78 | 20 | 50 | 50 | 45.45 | 30.77 | 19.05 | 54.55 | 22.22 | 25 | 30.77 | 36.36 | 22.22 | 33.33 | 38.41 |
| *N*_AR_ | 3.57 | 2.00 | 6.70 | 1.00 | 2.00 | 3.99 | 4.92 | 3.56 | 6.57 | 5.59 | 3.92 | 3.58 | 9.83 | 3.53 | 3.94 | 4.13 | 4.3 |
| *H*_O_ | 0.53 | 0.3 | 0.66 | - | 0.17 | 0.49 | 0.47 | 0.23 | 0.45 | 0.44 | 0.31 | 0.5 | 0.74 | 0.25 | 0.22 | 0.42 | 0.39 |
| *H*_E_ | 0.47 | 0.35 | 0.7 | - | 0.16 | 0.55 | 0.64 | 0.28 | 0.45 | 0.68 | 0.34 | 0.53 | 0.79 | 0.22 | 0.3 | 0.48 | 0.43 |
| HWE | ns | ns | *** | - | ns | ns | *** | * | *** | ** | *** | ns | *** | ns | *** | ns | *** |
| Loch Round Glenhead | | | | | | | | | | | | | | | | | |
| RGL |  |  |  |  |  |  |  |  |  |  |  |  |  |  |  |  |  |
| *n* | 73 | 74 | 73 | 74 | 72 | 71 | 71 | 72 | 72 | 74 | 72 | 70 | 70 | 74 | 69 | 72 | 72.06 |
| *N*_A_ | 3 | 2 | 6 | 1 | 2 | 5 | 7 | 5 | 11 | 4 | 5 | 7 | 14 | 5 | 7 | 3 | 87 |
| A% | 30 | 100 | 26.09 | 20 | 50 | 62.5 | 63.64 | 38.46 | 26.19 | 36.36 | 27.78 | 43.75 | 35.9 | 45.45 | 38.89 | 20 | 41.56 |
| *N*_AR_ | 2.80 | 2.00 | 4.72 | 1.00 | 2.00 | 4.56 | 5.81 | 4.66 | 9.62 | 4.00 | 4.75 | 5.51 | 12.08 | 4.64 | 6.41 | 3.00 | 4.85 |
| *H*_O_ | 0.59 | 0.19 | 0.48 | - | 0.14 | 0.52 | 0.58 | 0.54 | 0.68 | 0.5 | 0.31 | 0.64 | 0.71 | 0.53 | 0.67 | 0.54 | 0.48 |
| *H*_E_ | 0.52 | 0.17 | 0.55 | - | 0.13 | 0.56 | 0.54 | 0.5 | 0.66 | 0.51 | 0.34 | 0.68 | 0.83 | 0.5 | 0.67 | 0.47 | 0.48 |
| HWE | ns | ns | ns | - | ns | ns | ns | ns | ns | ns | ns | *** | * | ns | ns | ns | *** |
| Loch Long Glenhead | | | | | | | | | | | | | | | | | |
| LGL |  |  |  |  |  |  |  |  |  |  |  |  |  |  |  |  |  |
| *n* | 53 | 53 | 53 | 53 | 52 | 52 | 53 | 53 | 51 | 53 | 51 | 53 | 52 | 53 | 53 | 53 | 52.56 |
| *N*_A_ | 2 | 2 | 6 | 1 | 2 | 3 | 5 | 3 | 4 | 2 | 3 | 3 | 6 | 2 | 5 | 3 | 52 |
| A% | 20 | 100 | 26.09 | 20 | 50 | 37.5 | 45.45 | 23.08 | 9.52 | 18.18 | 16.67 | 18.75 | 15.38 | 18.18 | 27.78 | 20 | 29.16 |
| *N*_AR_ | 1.92 | 1.81 | 5.13 | 1.00 | 2.00 | 2.97 | 4.37 | 2.92 | 3.59 | 2.00 | 2.83 | 2.57 | 5.84 | 1.57 | 4.12 | 3.00 | 2.98 |
| *H*_O_ | 0.06 | 0.04 | 0.68 | - | 0.33 | 0.52 | 0.47 | 0.21 | 0.45 | 0.25 | 0.55 | 0.45 | 0.63 | 0.02 | 0.47 | 0.34 | 0.34 |
| *H*_E_ | 0.06 | 0.04 | 0.62 | - | 0.46 | 0.52 | 0.39 | 0.22 | 0.45 | 0.27 | 0.48 | 0.49 | 0.67 | 0.02 | 0.5 | 0.32 | 0.34 |
| HWE | ns | ns | *** | - | ns | ns | ns | ns | ns | ns | ns | ns | *** | ns | *** | ns | *** |
| River Girvan | | | | | | | | | | | | | | | | | |
| GIR |  |  |  |  |  |  |  |  |  |  |  |  |  |  |  |  |  |
| *n* | 42 | 44 | 42 | 39 | 44 | 41 | 22 | 32 | 33 | 38 | 43 | 38 | 40 | 44 | 44 | 43 | 39.31 |
| *N*_A_ | 6 | 2 | 14 | 3 | 2 | 7 | 7 | 9 | 22 | 8 | 12 | 9 | 25 | 6 | 11 | 8 | 151 |
| A% | 60 | 100 | 60.87 | 60 | 50 | 87.5 | 63.64 | 69.23 | 52.38 | 72.73 | 66.67 | 56.25 | 64.1 | 54.55 | 61.11 | 53.33 | 64.52 |
| *N*_AR_ | 5.63 | 2.00 | 13.17 | 3.00 | 2.00 | 6.86 | 7.00 | 8.93 | 21.42 | 7.91 | 11.37 | 8.58 | 22.46 | 5.64 | 10.25 | 7.38 | 8.98 |
| *H*_O_ | 0.64 | 0.5 | 0.93 | 0.18 | 0.18 | 0.68 | 0.59 | 0.72 | 0.85 | 0.79 | 0.81 | 0.71 | 0.9 | 0.59 | 0.84 | 0.72 | 0.67 |
| *H*_E_ | 0.71 | 0.45 | 0.88 | 0.27 | 0.3 | 0.76 | 0.82 | 0.77 | 0.94 | 0.74 | 0.89 | 0.82 | 0.93 | 0.58 | 0.85 | 0.67 | 0.71 |
| HWE | ns | ns | ns | ns | * | ns | ns | ** | * | ns | ns | ns | ** | ns | ns | ns | ** |
| Loch Eye |  |  |  |  |  |  |  |  |  |  |  |  |  |  |  |  |  |
| EYE |  |  |  |  |  |  |  |  |  |  |  |  |  |  |  |  |  |
| *n* | 48 | 48 | 48 | 48 | 48 | 48 | 48 | 48 | 48 | 48 | 48 | 48 | 48 | 48 | 48 | 48 | 48 |
| *N*_A_ | 2 | 2 | 8 | 1 | 2 | 3 | 3 | 3 | 3 | 2 | 6 | 5 | 12 | 3 | 6 | 3 | 64 |
| A% | 20 | 100 | 34.78 | 20 | 50 | 37.5 | 27.27 | 23.08 | 7.14 | 18.18 | 33.33 | 31.25 | 30.77 | 27.27 | 33.33 | 20 | 32.12 |
| *N*_AR_ | 2.00 | 2.00 | 7.10 | 1.00 | 2.00 | 3.00 | 2.95 | 3.00 | 3.00 | 2.00 | 5.25 | 4.25 | 10.71 | 2.99 | 5.62 | 3.00 | 3.74 |
| *H*_O_ | 0.44 | 0.08 | 0.75 | - | 0.12 | 0.62 | 0.27 | 0.5 | 0.35 | 0.31 | 0.6 | 0.35 | 0.81 | 0.23 | 0.6 | 0.48 | 0.41 |
| *H*_E_ | 0.42 | 0.15 | 0.65 | - | 0.12 | 0.64 | 0.24 | 0.49 | 0.52 | 0.26 | 0.53 | 0.42 | 0.77 | 0.24 | 0.67 | 0.47 | 0.41 |
| HWE | ns | ** | ns | - | ns | ns | ns | ns | ** | ns | ns | ns | ns | ns | ns | ns | ns |
| Loch Cornish | | | | | | | | | | | | | | | | | |
| COR |  |  |  |  |  |  |  |  |  |  |  |  |  |  |  |  |  |
| *n* | 51 | 51 | 51 | 51 | 51 | 51 | 51 | 51 | 51 | 51 | 51 | 51 | 51 | 51 | 51 | 51 | 51 |
| *N*_A_ | 4 | 2 | 8 | 1 | 2 | 3 | 2 | 5 | 6 | 3 | 7 | 6 | 13 | 3 | 6 | 5 | 76 |
| A% | 40 | 100 | 34.78 | 20 | 50 | 37.5 | 18.18 | 38.46 | 14.29 | 27.27 | 38.89 | 37.5 | 33.33 | 27.27 | 33.33 | 33.33 | 36.51 |
| *N*_AR_ | 3.52 | 2.00 | 7.66 | 1.00 | 2.00 | 3.00 | 2.00 | 4.93 | 5.93 | 2.59 | 6.97 | 5.42 | 11.54 | 3.00 | 5.42 | 4.35 | 4.46 |
| *H*_O_ | 0.59 | 0.41 | 0.76 | - | 0.24 | 0.45 | 0.37 | 0.69 | 0.67 | 0.35 | 0.78 | 0.41 | 0.82 | 0.33 | 0.55 | 0.39 | 0.49 |
| *H*_E_ | 0.51 | 0.44 | 0.77 | - | 0.24 | 0.44 | 0.35 | 0.69 | 0.64 | 0.49 | 0.8 | 0.51 | 0.83 | 0.32 | 0.56 | 0.41 | 0.5 |
| HWE | ns | ns | ns | - | ns | ns | ns | ns | ns | ns | ns | ns | ns | ns | ns | * | ns |
| Loch Brecbowie | | | | | | | | | | | | | | | | | |
| BRE |  |  |  |  |  |  |  |  |  |  |  |  |  |  |  |  |  |
| *n* | 58 | 58 | 58 | 58 | 58 | 56 | 58 | 58 | 58 | 57 | 58 | 58 | 58 | 58 | 58 | 58 | 57.81 |
| *N*_A_ | 4 | 2 | 7 | 1 | 3 | 4 | 7 | 4 | 10 | 4 | 9 | 6 | 13 | 3 | 8 | 3 | 88 |
| A% | 40 | 100 | 30.43 | 20 | 75 | 50 | 63.64 | 30.77 | 23.81 | 36.36 | 50 | 37.5 | 33.33 | 27.27 | 44.44 | 20 | 42.66 |
| *N*_AR_ | 3.98 | 2.00 | 6.29 | 1.00 | 2.95 | 4.00 | 5.96 | 4.00 | 8.84 | 3.48 | 7.96 | 5.69 | 10.67 | 3.00 | 7.33 | 3.00 | 5.01 |
| *H*_O_ | 0.67 | 0.29 | 0.74 | - | 0.17 | 0.73 | 0.47 | 0.69 | 0.53 | 0.09 | 0.72 | 0.69 | 0.81 | 0.38 | 0.76 | 0.5 | 0.52 |
| *H*_E_ | 0.69 | 0.32 | 0.75 | - | 0.16 | 0.73 | 0.55 | 0.69 | 0.67 | 0.24 | 0.75 | 0.66 | 0.81 | 0.34 | 0.74 | 0.52 | 0.54 |
| HWE | ns | ns | ns | - | ns | ns | ns | ns | ns | *** | ns | ns | ns | ns | ns | ns | ** |
| River Doon | | | | | | | | | | | | | | | | | |
| RDO |  |  |  |  |  |  |  |  |  |  |  |  |  |  |  |  |  |
| *n* | 37 | 38 | 37 | 38 | 38 | 35 | 21 | 31 | 35 | 37 | 38 | 37 | 37 | 38 | 35 | 38 | 35.62 |
| *N*_A_ | 5 | 2 | 15 | 1 | 3 | 5 | 6 | 5 | 22 | 8 | 12 | 8 | 23 | 6 | 11 | 8 | 140 |
| A% | 50 | 100 | 65.22 | 20 | 75 | 62.5 | 54.55 | 38.46 | 52.38 | 72.73 | 66.67 | 50 | 58.97 | 54.55 | 61.11 | 53.33 | 58.47 |
| *N*_AR_ | 4.81 | 2.00 | 13.98 | 1.00 | 3.00 | 5.00 | 6.00 | 5.00 | 20.99 | 7.81 | 11.91 | 7.93 | 20.99 | 5.58 | 10.82 | 7.49 | 8.39 |
| *H*_O_ | 0.51 | 0.37 | 0.86 | - | 0.24 | 0.57 | 0.48 | 0.65 | 0.74 | 0.54 | 0.87 | 0.78 | 0.92 | 0.63 | 0.83 | 0.68 | 0.6 |
| *H*_E_ | 0.67 | 0.43 | 0.88 | - | 0.33 | 0.75 | 0.63 | 0.71 | 0.92 | 0.77 | 0.89 | 0.83 | 0.92 | 0.7 | 0.86 | 0.72 | 0.69 |
| HWE | ns | ns | ns | - | ns | * | ns | ns | *** | *** | ns | ns | ns | ns | ns | ns | *** |
| Loch Doon | | | | | | | | | | | | | | | | | |
| LDO |  |  |  |  |  |  |  |  |  |  |  |  |  |  |  |  |  |
| *n* | 59 | 78 | 77 | 77 | 78 | 78 | 64 | 77 | 77 | 78 | 78 | 77 | 78 | 78 | 77 | 78 | 75.56 |
| *N*_A_ | 4 | 2 | 11 | 1 | 3 | 6 | 8 | 5 | 18 | 6 | 9 | 8 | 25 | 4 | 14 | 9 | 133 |
| A% | 40 | 100 | 47.83 | 20 | 75 | 75 | 72.73 | 38.46 | 42.86 | 54.55 | 50 | 50 | 64.1 | 36.36 | 77.78 | 60 | 56.54 |
| *N*_AR_ | 3.88 | 2.00 | 10.58 | 1.00 | 2.38 | 5.09 | 6.65 | 4.62 | 11.10 | 4.99 | 7.52 | 7.26 | 20.41 | 4.00 | 11.14 | 7.00 | 6.85 |
| *H*_O_ | 0.44 | 0.35 | 0.86 | - | 0.19 | 0.69 | 0.73 | 0.7 | 0.69 | 0.33 | 0.59 | 0.86 | 0.97 | 0.65 | 0.68 | 0.59 | 0.58 |
| *H*_E_ | 0.6 | 0.38 | 0.87 | - | 0.17 | 0.6 | 0.73 | 0.64 | 0.63 | 0.4 | 0.68 | 0.83 | 0.94 | 0.65 | 0.77 | 0.71 | 0.6 |
| HWE | ** | ns | ns | - | ns | ns | ns | ns | ns | *** | ns | ns | ns | ns | ns | ns | ns |
| Loch Enoch | | | | | | | | | | | | | | | | | |
| ENO_96_ |  |  |  |  |  |  |  |  |  |  |  |  |  |  |  |  |  |
| *n* | 55 | 56 | 56 | 56 | 55 | 56 | 56 | 56 | 56 | 56 | 56 | 56 | 56 | 56 | 56 | 56 | 55.88 |
| *N*_A_ | 4 | 2 | 10 | 1 | 2 | 5 | 5 | 4 | 9 | 4 | 8 | 3 | 11 | 4 | 7 | 5 | 84 |
| A% | 40 | 100 | 43.48 | 20 | 50 | 62.5 | 45.45 | 30.77 | 21.43 | 36.36 | 44.44 | 18.75 | 28.21 | 36.36 | 38.89 | 33.33 | 40.62 |
| *N*_AR_ | 4.00 | 2.00 | 9.27 | 1.00 | 2.00 | 5.00 | 5.00 | 3.90 | 8.31 | 3.98 | 7.53 | 2.96 | 9.58 | 3.86 | 6.52 | 4.99 | 4.99 |
| *H*_O_ | 0.76 | 0.48 | 0.86 | - | 0.15 | 0.73 | 0.71 | 0.66 | 0.93 | 0.54 | 0.77 | 0.48 | 0.91 | 0.3 | 0.86 | 0.75 | 0.62 |
| *H*_E_ | 0.69 | 0.43 | 0.84 | - | 0.13 | 0.76 | 0.75 | 0.68 | 0.82 | 0.66 | 0.83 | 0.51 | 0.82 | 0.37 | 0.72 | 0.72 | 0.61 |
| HWE | ns | ns | ns | - | ns | ** | ** | ns | ns | ns | ** | ns | ns | ns | ns | ns | ** |
| ENO_11-12_ |  |  |  |  |  |  |  |  |  |  |  |  |  |  |  |  |  |
| *n* | 16 | 16 | 16 | 16 | 16 | 16 | 15 | 16 | 16 | 16 | 16 | 16 | 16 | 16 | 16 | 16 | 15.94 |
| *N*_A_ | 4 | 2 | 8 | 1 | 2 | 4 | 3 | 4 | 8 | 4 | 6 | 3 | 8 | 3 | 6 | 5 | 71 |
| A% | 40 | 100 | 34.78 | 20 | 50 | 50 | 27.27 | 30.77 | 19.05 | 36.36 | 33.33 | 18.75 | 20.51 | 27.27 | 33.33 | 33.33 | 35.92 |
| *N*_AR_ | 4 | 2 | 8 | 1 | 2 | 4 | 3 | 4 | 8 | 4 | 6 | 3 | 8 | 3 | 6 | 5 | 4.44 |
| *H*_O_ | 0.75 | 0.19 | 0.88 | - | 0.06 | 0.69 | 0.47 | 0.69 | 0.69 | 0.62 | 0.81 | 0.5 | 0.75 | 0.25 | 0.75 | 0.75 | 0.55 |
| *H*_E_ | 0.65 | 0.34 | 0.82 | - | 0.06 | 0.68 | 0.62 | 0.69 | 0.7 | 0.71 | 0.79 | 0.42 | 0.82 | 0.22 | 0.68 | 0.7 | 0.56 |
| HWE | ns | ns | ns | - | ns | ns | ns | ns | ns | ns | ns | ns | ns | ns | ns | ns | ns |
| Loch Twachtan | | | | | | | | | | | | | | | | | |
| TWA |  |  |  |  |  |  |  |  |  |  |  |  |  |  |  |  |  |
| *n* | 20 | 20 | 20 | 20 | 20 | 20 | 19 | 20 | 20 | 19 | 20 | 20 | 19 | 20 | 20 | 20 | 19.81 |
| *N*_A_ | 3 | 2 | 6 | 1 | 2 | 5 | 5 | 3 | 3 | 3 | 6 | 5 | 11 | 4 | 5 | 4 | 68 |
| A% | 30 | 100 | 26.09 | 20 | 50 | 62.5 | 45.45 | 23.08 | 7.14 | 27.27 | 33.33 | 31.25 | 28.21 | 36.36 | 27.78 | 26.67 | 35.95 |
| *N*_AR_ | 3 | 2 | 6 | 1 | 2 | 5 | 5 | 3 | 3 | 3 | 6 | 5 | 11 | 4 | 5 | 4 | 4.25 |
| *H*_O_ | 0.4 | 0.6 | 0.6 | - | 0.55 | 0.8 | 0.74 | 0.5 | 0.35 | 0.58 | 0.6 | 0.55 | 1 | 0.55 | 0.45 | 0.65 | 0.56 |
| *H*_E_ | 0.5 | 0.5 | 0.64 | - | 0.5 | 0.73 | 0.7 | 0.52 | 0.36 | 0.5 | 0.57 | 0.43 | 0.87 | 0.61 | 0.4 | 0.54 | 0.52 |
| HWE | ns | ns | ns | - | ns | ns | ns | ns | ns | ns | ns | ns | ns | ns | ns | ns | ns |
| Loch Dry |  |  |  |  |  |  |  |  |  |  |  |  |  |  |  |  |  |
| DRY |  |  |  |  |  |  |  |  |  |  |  |  |  |  |  |  |  |
| *n* | 47 | 47 | 47 | 47 | 47 | 47 | 47 | 47 | 47 | 47 | 47 | 47 | 47 | 47 | 47 | 47 | 47 |
| *N*_A_ | 3 | 2 | 8 | 1 | 2 | 3 | 6 | 4 | 7 | 4 | 4 | 5 | 8 | 3 | 5 | 7 | 72 |
| A% | 30 | 100 | 34.78 | 20 | 50 | 37.5 | 54.55 | 30.77 | 16.67 | 36.36 | 22.22 | 31.25 | 20.51 | 27.27 | 27.78 | 46.67 | 36.65 |
| *N*_AR_ | 3 | 2 | 6 | 1 | 2 | 5 | 5 | 3 | 3 | 3 | 6 | 5 | 11 | 4 | 5 | 4 | 4.16 |
| *H*_O_ | 0.3 | 0.47 | 0.68 | - | 0.19 | 0.49 | 0.68 | 0.7 | 0.83 | 0.53 | 0.55 | 0.72 | 0.47 | 0.49 | 0.51 | 0.68 | 0.52 |
| *H*_E_ | 0.35 | 0.45 | 0.77 | - | 0.21 | 0.59 | 0.7 | 0.55 | 0.73 | 0.66 | 0.57 | 0.72 | 0.53 | 0.46 | 0.58 | 0.7 | 0.53 |
| HWE | ns | ns | *** | - | ns | ns | * | *** | ns | ns | * | ** | *** | ns | * | ns | *** |
| Loch Riecawr | | | | | | | | | | | | | | | | | |
| RIE |  |  |  |  |  |  |  |  |  |  |  |  |  |  |  |  |  |
| *n* | 20 | 20 | 20 | 20 | 20 | 20 | 20 | 20 | 20 | 19 | 20 | 19 | 20 | 20 | 19 | 20 | 19.81 |
| *N*_A_ | 4 | 2 | 10 | 1 | 2 | 4 | 4 | 4 | 10 | 4 | 6 | 6 | 16 | 4 | 8 | 6 | 91 |
| A% | 40 | 100 | 43.48 | 20 | 50 | 50 | 36.36 | 30.77 | 23.81 | 36.36 | 33.33 | 37.5 | 41.03 | 36.36 | 44.44 | 40 | 41.46 |
| *N*_AR_ | 4 | 2 | 10 | 1 | 2 | 4 | 4 | 4 | 10 | 4 | 6 | 6 | 16 | 4 | 8 | 6 | 5.69 |
| *H*_O_ | 0.65 | 0.4 | 0.95 | - | 0.1 | 0.75 | 0.65 | 0.5 | 0.5 | 0.32 | 0.75 | 0.84 | 0.75 | 0.7 | 0.74 | 0.7 | 0.58 |
| *H*_E_ | 0.69 | 0.38 | 0.88 | - | 0.1 | 0.62 | 0.63 | 0.53 | 0.5 | 0.41 | 0.65 | 0.73 | 0.9 | 0.59 | 0.7 | 0.73 | 0.56 |
| HWE | ns | ns | ns | - | ns | ns | ns | ns | ns | ns | ns | ns | ** | ns | ns | ns | ns |
| Loch Macaterick | | | | | | | | | | | | | | | | | |
| MAC |  |  |  |  |  |  |  |  |  |  |  |  |  |  |  |  |  |
| *n* | 51 | 51 | 51 | 51 | 51 | 51 | 50 | 50 | 51 | 51 | 51 | 49 | 49 | 51 | 50 | 51 | 50.56 |
| *N*_A_ | 5 | 2 | 12 | 1 | 2 | 5 | 7 | 4 | 10 | 3 | 6 | 6 | 23 | 5 | 9 | 6 | 106 |
| A% | 50 | 100 | 52.17 | 20 | 50 | 62.5 | 63.64 | 30.77 | 23.81 | 27.27 | 33.33 | 37.5 | 58.97 | 45.45 | 50 | 40 | 46.59 |
| *N*_AR_ | 4.18 | 2.00 | 11.33 | 1.00 | 1.97 | 4.93 | 6.38 | 3.60 | 8.27 | 2.59 | 5.35 | 5.94 | 20.67 | 4.59 | 8.53 | 5.93 | 6.08 |
| *H*_O_ | 0.63 | 0.22 | 0.94 | - | 0.08 | 0.61 | 0.58 | 0.64 | 0.53 | 0.53 | 0.47 | 0.82 | 0.96 | 0.65 | 0.74 | 0.75 | 0.57 |
| *H*_E_ | 0.59 | 0.25 | 0.89 | - | 0.08 | 0.64 | 0.68 | 0.56 | 0.5 | 0.48 | 0.54 | 0.74 | 0.93 | 0.64 | 0.73 | 0.69 | 0.56 |
| HWE | ns | ns | *** | - | ns | * | ns | ns | ns | ns | ns | ns | ns | ns | ns | ns | ns |
| River Annan | | | | | | | | | | | | | | | | | |
| ANN |  |  |  |  |  |  |  |  |  |  |  |  |  |  |  |  |  |
| *n* | 39 | 39 | 39 | 39 | 39 | 39 | 39 | 39 | 39 | 39 | 39 | 39 | 39 | 39 | 39 | 39 | 39 |
| *N*_A_ | 6 | 2 | 17 | 3 | 2 | 5 | 7 | 6 | 17 | 7 | 12 | 7 | 25 | 6 | 9 | 7 | 138 |
| A% | 60 | 100 | 73.91 | 60 | 50 | 62.5 | 63.64 | 46.15 | 40.48 | 63.64 | 66.67 | 43.75 | 64.1 | 54.55 | 50 | 46.67 | 59.13 |
| *N*_AR_ | 5.72 | 2.00 | 15.73 | 2.77 | 2.00 | 5.00 | 6.90 | 5.77 | 15.81 | 6.94 | 11.41 | 6.95 | 23.09 | 5.76 | 8.93 | 6.53 | 8.21 |
| *H*_O_ | 0.59 | 0.33 | 0.85 | 0.15 | 0.13 | 0.64 | 0.54 | 0.46 | 0.82 | 0.74 | 0.72 | 0.87 | 0.95 | 0.54 | 0.72 | 0.69 | 0.61 |
| *H*_E_ | 0.63 | 0.49 | 0.9 | 0.14 | 0.16 | 0.75 | 0.66 | 0.72 | 0.88 | 0.77 | 0.84 | 0.83 | 0.93 | 0.69 | 0.83 | 0.72 | 0.68 |
| HWE | ns | ns | ns | ns | ns | ns | ns | * | ns | ns | ns | ** | ns | ns | * | ns | ns |
| Loch Leven | | | | | | | | | | | | | | | | | |
| LEV |  |  |  |  |  |  |  |  |  |  |  |  |  |  |  |  |  |
| *n* | 62 | 62 | 61 | 62 | 62 | 62 | 61 | 60 | 62 | 61 | 62 | 62 | 62 | 62 | 62 | 62 | 61.69 |
| *N*_A_ | 5 | 2 | 16 | 2 | 3 | 6 | 6 | 7 | 19 | 7 | 15 | 6 | 28 | 6 | 8 | 6 | 142 |
| A% | 50 | 100 | 69.57 | 40 | 75 | 75 | 54.55 | 53.85 | 45.24 | 63.64 | 83.33 | 37.5 | 71.79 | 54.55 | 44.44 | 40 | 59.9 |
| *N*_AR_ | 4.73 | 2.00 | 14.16 | 1.93 | 2.93 | 5.97 | 5.87 | 5.98 | 16.39 | 6.13 | 12.48 | 6.00 | 23.33 | 4.83 | 7.99 | 5.47 | 7.89 |
| *H*_O_ | 0.68 | 0.35 | 0.95 | 0.06 | 0.26 | 0.77 | 0.59 | 0.67 | 0.94 | 0.39 | 0.9 | 0.68 | 0.94 | 0.56 | 0.79 | 0.66 | 0.64 |
| *H*_E_ | 0.69 | 0.4 | 0.89 | 0.06 | 0.26 | 0.73 | 0.77 | 0.68 | 0.92 | 0.39 | 0.86 | 0.68 | 0.95 | 0.58 | 0.79 | 0.71 | 0.65 |
| HWE | ns | ns | ns | ns | ns | ns | * | ns | ns | ns | ns | ns | ns | ns | ns | ns | ns |
| Howietoun fish farm | | | | | | | | | | | | | | | | | |
| HOW |  |  |  |  |  |  |  |  |  |  |  |  |  |  |  |  |  |
| *n* | 48 | 48 | 48 | 48 | 48 | 48 | 48 | 48 | 48 | 48 | 48 | 46 | 46 | 48 | 46 | 48 | 47.62 |
| *N*_A_ | 4 | 2 | 14 | 3 | 3 | 5 | 5 | 4 | 16 | 6 | 10 | 7 | 20 | 4 | 9 | 8 | 120 |
| A% | 40 | 100 | 60.87 | 60 | 75 | 62.5 | 45.45 | 30.77 | 38.1 | 54.55 | 55.56 | 43.75 | 51.28 | 36.36 | 50 | 53.33 | 53.59 |
| *N*_AR_ | 3.86 | 2.00 | 13.00 | 2.62 | 2.98 | 5.00 | 4.98 | 4.00 | 14.78 | 5.91 | 9.44 | 6.98 | 18.48 | 3.86 | 8.17 | 6.97 | 7.07 |
| *H*_O_ | 0.58 | 0.58 | 0.9 | 0.19 | 0.19 | 0.67 | 0.67 | 0.65 | 0.92 | 0.48 | 0.83 | 0.57 | 0.96 | 0.71 | 0.76 | 0.81 | 0.65 |
| *H*_E_ | 0.59 | 0.43 | 0.88 | 0.19 | 0.17 | 0.62 | 0.74 | 0.63 | 0.9 | 0.56 | 0.82 | 0.67 | 0.93 | 0.65 | 0.73 | 0.72 | 0.64 |
| HWE | ns | * | ns | * | ns | ns | ** | ns | ns | * | ns | ns | ns | ns | ns | ns | ns |
