# Supplementary material for: Impacts of acidification on brown trout Salmo trutta populations and the contribution of stocking to population recovery and genetic diversity
Source: J Fish Biol. 2019 Jun 24;95(3):719–42. doi: 10.1111/jfb.14054 (PMC6852074; doi:10.1111/jfb.14054)
Supplement: Supplementary file 8 — TABLE S3. D EST and F ST pairwise values between STRUCTURE or Bayesian analysis of population structure program (BAPS; GRA and FLE only) inferred populations. In each case, estimates are shown using a colour heat map (–, low–high level of between population genetics divergence) below diagonal (). Values in parentheses above diagonal indicate 95% CI for estimates. , non‐significant pairwise comparisons are highlighted in yellow. n.b. Natural populations have had admixed individuals removed. GRA1, 2 and 3, and FLE1 and 2 are the separate populations identified in these lochs with in the former only the GRA10‐12 sample being used in the computation. [file JFB-95-719-s008.docx]

**TABLE S3** *D*_EST_ and *F*_ST_ pairwise values between STRUCTURE or Bayesian analysis of population structure program (BAPS; GRA and FLE only) inferred populations. In each case, estimates are shown using a colour heat map (█–█, low–high level of between population genetics divergence) below diagonal (█). Values in parentheses above diagonal indicate 95% CI for estimates. █, non-significant pairwise comparisons are highlighted in yellow. *n.b*. Natural populations have had admixed individuals removed. GRA1, 2 and 3, and FLE1 and 2 are the separate populations identified in these lochs with in the former only the GRA10-12 sample being used in the computation

|  | **Loch Grannoch** | | | **River Deuch** | **River Ken** | **Loch Dungeon** | **Loch Harrow** | **Lochinvar** | **Loch Dee & tributaries** | **River Shirmers** | **Loch Mannoch** | **Loch Fleet** | | **River Water of Fleet** | **River Palnure** | **Loch Valley** | **Loch Narroch** | | **Loch Neldricken** | | **Loch Round Glenhead** | **Loch Long Glenhead** | **River Girvan** | **Loch Eye** | **Loch Cornish** | **Loch Brecbowie** | **River Doon** | **Loch Doon** | **Loch Enoch** | | **Loch Twachtan** | **Loch Dry** | **Loch Riecawr** | **Loch Macaterick** | **River Annan** | **Loch Leven** | **Howietoun fish farm** |
| --- | --- | --- | --- | --- | --- | --- | --- | --- | --- | --- | --- | --- | --- | --- | --- | --- | --- | --- | --- | --- | --- | --- | --- | --- | --- | --- | --- | --- | --- | --- | --- | --- | --- | --- | --- | --- | --- |
|  | **GRA1** | **GRA2** | **GRA3** | **DEU** | **KEN** | **DUN** | **HAR** | **INV** | **DEE** | **SHI** | **MAN** | **FLE1** | **FLE2** | **WOF** | **PAL** | **VAL** | **NAR_00-02_** | **NAR_12_** | **NEL_01_** | **NEL_11_** | **RGL** | **LGL** | **GIR** | **EYE** | **COR** | **BRE** | **RDO** | **LDO** | **ENO_96_** | **ENO_11-12_** | **TWA** | **DRY** | **RIE** | **MAC** | **ANN** | **LEV** | **HOW** |
| *D*_ST_ |  |  |  |  |  |  |  |  |  |  |  |  |  |  |  |  |  |  |  |  |  |  |  |  |  |  |  |  |  |  |  |  |  |  |  |  |  |
| GRA1 |  | (0.01-0.056) | (0.019-0.053) | (0.17-0.236) | (0.152-0.222) | (0.295-0.381) | (0.195-0.258) | (0.162-0.236) | (0.103-0.168) | (0.206-0.272) | (0.178-0.27) | (0.097-0.156) | (0.127-0.191) | (0.141-0.203) | (0.118-0.191) | (0.236-0.322) | (0.078-0.151) | (0.049-0.141) | (0.436-0.498) | (0.281-0.364) | (0.34-0.417) | (0.455-0.532) | (0.166-0.247) | (0.375-0.451) | (0.305-0.385) | (0.235-0.318) | (0.129-0.214) | (0.178-0.278) | (0.04-0.104) | (0.008-0.078) | (0.199-0.316) | (0.176-0.24) | (0.214-0.304) | (0.229-0.309) | (0.148-0.238) | (0.193-0.264) | (0.223-0.302) |
| GRA2 | 0.033 |  | (0.014-0.033) | (0.157-0.206) | (0.164-0.219) | (0.266-0.327) | (0.21-0.261) | (0.138-0.203) | (0.116-0.161) | (0.147-0.199) | (0.143-0.218) | (0.109-0.153) | (0.14-0.184) | (0.17-0.22) | (0.143-0.205) | (0.205-0.281) | (0.022-0.052) | (0.021-0.118) | (0.422-0.477) | (0.272-0.339) | (0.374-0.437) | (0.506-0.568) | (0.17-0.238) | (0.353-0.413) | (0.307-0.364) | (0.256-0.319) | (0.153-0.214) | (0.196-0.253) | (0.009-0.049) | (-0.003-0.042) | (0.283-0.365) | (0.204-0.259) | (0.192-0.275) | (0.238-0.293) | (0.176-0.248) | (0.153-0.213) | (0.172-0.235) |
| GRA3 | 0.047 | 0.023 |  | (0.166-0.205) | (0.165-0.218) | (0.271-0.307) | (0.205-0.242) | (0.139-0.19) | (0.125-0.156) | (0.147-0.186) | (0.138-0.193) | (0.108-0.145) | (0.137-0.164) | (0.154-0.193) | (0.139-0.193) | (0.211-0.288) | (0.041-0.065) | (0.017-0.1) | (0.45-0.485) | (0.277-0.342) | (0.338-0.385) | (0.474-0.519) | (0.145-0.197) | (0.348-0.383) | (0.298-0.337) | (0.224-0.27) | (0.146-0.2) | (0.2-0.242) | (0.015-0.039) | (0.005-0.048) | (0.256-0.336) | (0.199-0.25) | (0.185-0.264) | (0.239-0.281) | (0.152-0.216) | (0.13-0.176) | (0.145-0.198) |
| DEU | 0.201 | 0.181 | 0.185 |  | (0.039-0.076) | (0.135-0.184) | (0.118-0.166) | (0.143-0.202) | (0.128-0.17) | (0.171-0.225) | (0.105-0.175) | (0.108-0.149) | (0.088-0.127) | (0.133-0.181) | (0.088-0.154) | (0.204-0.254) | (0.164-0.22) | (0.133-0.238) | (0.339-0.388) | (0.212-0.275) | (0.221-0.276) | (0.244-0.306) | (0.113-0.178) | (0.339-0.4) | (0.289-0.353) | (0.171-0.227) | (0.071-0.143) | (0.094-0.139) | (0.163-0.218) | (0.188-0.258) | (0.151-0.225) | (0.195-0.267) | (0.096-0.155) | (0.097-0.147) | (0.08-0.13) | (0.139-0.203) | (0.128-0.203) |
| KEN | 0.187 | 0.192 | 0.192 | 0.057 |  | (0.133-0.182) | (0.118-0.161) | (0.163-0.228) | (0.122-0.17) | (0.225-0.297) | (0.135-0.217) | (0.1-0.139) | (0.071-0.115) | (0.163-0.23) | (0.118-0.183) | (0.195-0.245) | (0.178-0.236) | (0.133-0.225) | (0.294-0.348) | (0.186-0.245) | (0.161-0.206) | (0.185-0.242) | (0.171-0.259) | (0.346-0.416) | (0.296-0.365) | (0.252-0.315) | (0.15-0.231) | (0.131-0.186) | (0.162-0.226) | (0.157-0.247) | (0.205-0.287) | (0.177-0.232) | (0.096-0.159) | (0.111-0.162) | (0.109-0.179) | (0.175-0.242) | (0.169-0.169) |
| DUN | 0.337 | 0.296 | 0.290 | 0.159 | 0.158 |  | (0.11-0.145) | (0.215-0.276) | (0.196-0.235) | (0.266-0.316) | (0.237-0.299) | (0.184-0.23) | (0.194-0.233) | (0.274-0.322) | (0.212-0.278) | (0.281-0.323) | (0.341-0.396) | (0.237-0.351) | (0.315-0.351) | (0.266-0.311) | (0.272-0.317) | (0.244-0.288) | (0.264-0.338) | (0.415-0.47) | (0.342-0.394) | (0.31-0.359) | (0.223-0.314) | (0.227-0.274) | (0.306-0.368) | (0.288-0.387) | (0.297-0.393) | (0.331-0.387) | (0.188-0.283) | (0.226-0.273) | (0.227-0.3) | (0.211-0.266) | (0.238-0.305) |
| HAR | 0.225 | 0.235 | 0.224 | 0.142 | 0.139 | 0.127 |  | (0.17-0.226) | (0.211-0.249) | (0.264-0.317) | (0.167-0.228) | (0.203-0.242) | (0.162-0.202) | (0.229-0.284) | (0.16-0.216) | (0.282-0.326) | (0.273-0.338) | (0.226-0.321) | (0.372-0.413) | (0.284-0.337) | (0.32-0.372) | (0.308-0.359) | (0.213-0.276) | (0.391-0.441) | (0.333-0.383) | (0.251-0.309) | (0.172-0.242) | (0.208-0.255) | (0.219-0.28) | (0.217-0.299) | (0.24-0.326) | (0.233-0.299) | (0.199-0.283) | (0.217-0.272) | (0.147-0.21) | (0.202-0.259) | (0.195-0.254) |
| INV | 0.198 | 0.170 | 0.163 | 0.170 | 0.194 | 0.247 | 0.198 |  | (0.153-0.213) | (0.084-0.135) | (0.109-0.185) | (0.134-0.195) | (0.118-0.168) | (0.151-0.207) | (0.145-0.213) | (0.188-0.249) | (0.151-0.214) | (0.135-0.24) | (0.271-0.327) | (0.188-0.257) | (0.235-0.303) | (0.342-0.42) | (0.094-0.158) | (0.231-0.294) | (0.192-0.258) | (0.189-0.253) | (0.09-0.165) | (0.118-0.177) | (0.116-0.181) | (0.146-0.241) | (0.138-0.231) | (0.255-0.331) | (0.104-0.175) | (0.126-0.187) | (0.096-0.159) | (0.098-0.152) | (0.073-0.123) |
| DEE | 0.133 | 0.138 | 0.140 | 0.149 | 0.144 | 0.215 | 0.230 | 0.181 |  | (0.111-0.154) | (0.105-0.173) | (0.042-0.066) | (0.044-0.069) | (0.078-0.117) | (0.063-0.112) | (0.254-0.304) | (0.158-0.218) | (0.12-0.218) | (0.405-0.437) | (0.269-0.327) | (0.253-0.303) | (0.347-0.385) | (0.113-0.172) | (0.25-0.288) | (0.193-0.235) | (0.214-0.254) | (0.081-0.135) | (0.116-0.153) | (0.15-0.208) | (0.129-0.213) | (0.212-0.275) | (0.225-0.29) | (0.117-0.187) | (0.157-0.207) | (0.093-0.156) | (0.135-0.18) | (0.135-0.174) |
| SHI | 0.237 | 0.172 | 0.166 | 0.198 | 0.262 | 0.290 | 0.290 | 0.107 | 0.132 |  | (0.073-0.14) | (0.135-0.188) | (0.11-0.154) | (0.089-0.134) | (0.079-0.128) | (0.269-0.333) | (0.174-0.231) | (0.144-0.238) | (0.422-0.476) | (0.285-0.359) | (0.282-0.342) | (0.456-0.522) | (0.025-0.072) | (0.224-0.285) | (0.187-0.241) | (0.152-0.208) | (0.061-0.124) | (0.157-0.216) | (0.133-0.206) | (0.151-0.25) | (0.247-0.322) | (0.28-0.348) | (0.149-0.234) | (0.205-0.268) | (0.081-0.139) | (0.051-0.092) | (0.057-0.097) |
| MAN | 0.223 | 0.178 | 0.164 | 0.138 | 0.176 | 0.267 | 0.196 | 0.146 | 0.136 | 0.105 |  | (0.096-0.159) | (0.077-0.131) | (0.05-0.107) | (0.067-0.13) | (0.273-0.364) | (0.134-0.206) | (0.135-0.236) | (0.444-0.505) | (0.288-0.376) | (0.325-0.394) | (0.393-0.478) | (0.029-0.097) | (0.194-0.242) | (0.177-0.232) | (0.124-0.183) | (0.037-0.101) | (0.089-0.149) | (0.118-0.196) | (0.13-0.242) | (0.238-0.336) | (0.256-0.36) | (0.088-0.169) | (0.101-0.167) | (0.06-0.129) | (0.025-0.071) | (0.028-0.074) |
| FLE1 | 0.125 | 0.130 | 0.125 | 0.128 | 0.119 | 0.206 | 0.222 | 0.163 | 0.054 | 0.160 | 0.126 |  | (0.033-0.06) | (0.096-0.137) | (0.081-0.142) | (0.152-0.204) | (0.117-0.168) | (0.06-0.154) | (0.31-0.361) | (0.172-0.233) | (0.226-0.287) | (0.318-0.38) | (0.119-0.182) | (0.264-0.318) | (0.204-0.257) | (0.178-0.228) | (0.08-0.145) | (0.112-0.156) | (0.105-0.155) | (0.112-0.186) | (0.163-0.226) | (0.191-0.255) | (0.103-0.175) | (0.119-0.166) | (0.1-0.166) | (0.113-0.172) | (0.11-0.159) |
| FLE2 | 0.158 | 0.162 | 0.150 | 0.108 | 0.091 | 0.213 | 0.182 | 0.142 | 0.056 | 0.133 | 0.101 | 0.046 |  | (0.071-0.108) | (0.047-0.091) | (0.185-0.228) | (0.161-0.215) | (0.104-0.221) | (0.324-0.361) | (0.185-0.239) | (0.187-0.239) | (0.268-0.316) | (0.082-0.142) | (0.229-0.271) | (0.183-0.231) | (0.17-0.218) | (0.048-0.11) | (0.05-0.076) | (0.125-0.179) | (0.151-0.218) | (0.14-0.192) | (0.142-0.209) | (0.035-0.084) | (0.074-0.109) | (0.06-0.105) | (0.113-0.154) | (0.088-0.131) |
| WOF | 0.172 | 0.195 | 0.174 | 0.156 | 0.196 | 0.299 | 0.257 | 0.177 | 0.098 | 0.111 | 0.076 | 0.114 | 0.088 |  | (0.01-0.043) | (0.298-0.369) | (0.159-0.221) | (0.149-0.248) | (0.488-0.535) | (0.318-0.396) | (0.274-0.332) | (0.409-0.47) | (0.03-0.083) | (0.213-0.262) | (0.191-0.24) | (0.231-0.295) | (0.012-0.058) | (0.12-0.177) | (0.145-0.203) | (0.131-0.219) | (0.191-0.259) | (0.183-0.257) | (0.141-0.234) | (0.151-0.21) | (0.024-0.073) | (0.08-0.119) | (0.084-0.131) |
| PAL | 0.152 | 0.172 | 0.165 | 0.119 | 0.149 | 0.245 | 0.187 | 0.177 | 0.086 | 0.103 | 0.097 | 0.110 | 0.068 | 0.024 |  | (0.244-0.333) | (0.139-0.212) | (0.118-0.223) | (0.413-0.484) | (0.26-0.35) | (0.229-0.291) | (0.377-0.461) | (0.031-0.091) | (0.212-0.281) | (0.194-0.253) | (0.186-0.252) | (0.005-0.059) | (0.104-0.169) | (0.119-0.185) | (0.13-0.205) | (0.181-0.261) | (0.173-0.242) | (0.087-0.193) | (0.143-0.205) | (0.001-0.054) | (0.074-0.127) | (0.078-0.143) |
| VAL | 0.277 | 0.241 | 0.249 | 0.229 | 0.221 | 0.301 | 0.305 | 0.217 | 0.279 | 0.300 | 0.319 | 0.177 | 0.207 | 0.334 | 0.287 |  | (0.177-0.256) | (0.028-0.191) | (0.044-0.088) | (-0.003-0.012) | (0.184-0.227) | (0.261-0.316) | (0.236-0.315) | (0.306-0.376) | (0.297-0.355) | (0.266-0.327) | (0.245-0.355) | (0.196-0.247) | (0.161-0.239) | (0.233-0.32) | (0.163-0.248) | (0.22-0.275) | (0.163-0.222) | (0.176-0.229) | (0.207-0.283) | (0.249-0.322) | (0.27-0.344) |
| NAR_00-02_ | 0.112 | 0.036 | 0.052 | 0.191 | 0.205 | 0.367 | 0.306 | 0.180 | 0.187 | 0.201 | 0.167 | 0.140 | 0.186 | 0.188 | 0.175 | 0.216 |  | (-0.012-0.075) | (0.383-0.442) | (0.226-0.308) | (0.315-0.391) | (0.502-0.576) | (0.172-0.252) | (0.275-0.328) | (0.26-0.318) | (0.252-0.305) | (0.162-0.241) | (0.257-0.314) | (0.006-0.036) | (-0.003-0.044) | (0.253-0.346) | (0.241-0.316) | (0.235-0.335) | (0.238-0.298) | (0.16-0.246) | (0.156-0.215) | (0.167-0.231) |
| NAR_12_ | 0.088 | 0.058 | 0.050 | 0.180 | 0.173 | 0.290 | 0.270 | 0.183 | 0.164 | 0.190 | 0.183 | 0.099 | 0.153 | 0.195 | 0.168 | 0.106 | 0.019 |  | (0.185-0.397) | (0.061-0.256) | (0.159-0.25) | (0.35-0.452) | (0.132-0.241) | (0.245-0.329) | (0.239-0.318) | (0.178-0.272) | (0.141-0.26) | (0.185-0.325) | (-0.009-0.067) | (0.005-0.122) | (0.112-0.237) | (0.202-0.304) | (0.177-0.345) | (0.165-0.305) | (0.121-0.247) | (0.116-0.193) | (0.153-0.228) |
| NEL_01_ | 0.466 | 0.450 | 0.468 | 0.363 | 0.320 | 0.332 | 0.392 | 0.299 | 0.421 | 0.449 | 0.474 | 0.335 | 0.341 | 0.511 | 0.450 | 0.065 | 0.414 | 0.298 |  | (0.021-0.061) | (0.233-0.268) | (0.245-0.287) | (0.405-0.47) | (0.425-0.484) | (0.423-0.465) | (0.365-0.417) | (0.425-0.522) | (0.289-0.339) | (0.378-0.441) | (0.452-0.527) | (0.268-0.364) | (0.324-0.381) | (0.233-0.284) | (0.248-0.294) | (0.391-0.456) | (0.403-0.463) | (0.415-0.474) |
| NEL_11_ | 0.321 | 0.305 | 0.309 | 0.242 | 0.215 | 0.288 | 0.310 | 0.223 | 0.297 | 0.323 | 0.334 | 0.201 | 0.212 | 0.357 | 0.307 | 0.003 | 0.268 | 0.158 | 0.038 |  | (0.172-0.207) | (0.244-0.292) | (0.251-0.34) | (0.328-0.401) | (0.319-0.377) | (0.272-0.332) | (0.27-0.38) | (0.191-0.247) | (0.214-0.293) | (0.298-0.378) | (0.164-0.242) | (0.211-0.272) | (0.145-0.196) | (0.158-0.209) | (0.228-0.311) | (0.258-0.34) | (0.281-0.357) |
| RGL | 0.380 | 0.408 | 0.361 | 0.246 | 0.182 | 0.294 | 0.344 | 0.267 | 0.279 | 0.313 | 0.358 | 0.255 | 0.211 | 0.302 | 0.260 | 0.205 | 0.353 | 0.201 | 0.250 | 0.189 |  | (0.103-0.134) | (0.269-0.341) | (0.393-0.447) | (0.372-0.422) | (0.281-0.337) | (0.274-0.38) | (0.253-0.307) | (0.304-0.37) | (0.362-0.453) | (0.189-0.249) | (0.248-0.309) | (0.236-0.287) | (0.217-0.26) | (0.197-0.258) | (0.246-0.305) | (0.23-0.291) |
| LGL | 0.495 | 0.536 | 0.497 | 0.275 | 0.210 | 0.266 | 0.334 | 0.378 | 0.365 | 0.488 | 0.435 | 0.348 | 0.291 | 0.441 | 0.419 | 0.288 | 0.539 | 0.399 | 0.265 | 0.266 | 0.117 |  | (0.419-0.499) | (0.578-0.637) | (0.543-0.606) | (0.402-0.468) | (0.406-0.493) | (0.268-0.323) | (0.472-0.537) | (0.513-0.612) | (0.205-0.281) | (0.309-0.36) | (0.218-0.272) | (0.251-0.305) | (0.314-0.397) | (0.402-0.471) | (0.338-0.41) |
| GIR | 0.205 | 0.204 | 0.169 | 0.146 | 0.215 | 0.299 | 0.244 | 0.124 | 0.141 | 0.047 | 0.060 | 0.149 | 0.110 | 0.055 | 0.058 | 0.274 | 0.207 | 0.184 | 0.439 | 0.296 | 0.304 | 0.458 |  | (0.164-0.232) | (0.138-0.194) | (0.142-0.203) | (-0.003-0.052) | (0.102-0.161) | (0.117-0.192) | (0.158-0.241) | (0.202-0.299) | (0.231-0.327) | (0.086-0.17) | (0.128-0.193) | (0.028-0.104) | (0.034-0.087) | (0.042-0.105) |
| EYE | 0.413 | 0.384 | 0.366 | 0.369 | 0.381 | 0.443 | 0.417 | 0.262 | 0.268 | 0.253 | 0.217 | 0.290 | 0.249 | 0.238 | 0.244 | 0.341 | 0.302 | 0.286 | 0.456 | 0.366 | 0.419 | 0.608 | 0.195 |  | (0.016-0.041) | (0.226-0.279) | (0.176-0.244) | (0.21-0.262) | (0.252-0.319) | (0.294-0.386) | (0.306-0.385) | (0.367-0.452) | (0.206-0.273) | (0.184-0.25) | (0.239-0.307) | (0.189-0.231) | (0.197-0.249) |
| COR | 0.346 | 0.335 | 0.317 | 0.320 | 0.331 | 0.368 | 0.357 | 0.225 | 0.214 | 0.213 | 0.203 | 0.231 | 0.206 | 0.215 | 0.223 | 0.326 | 0.287 | 0.277 | 0.444 | 0.348 | 0.397 | 0.575 | 0.165 | 0.028 |  | (0.204-0.265) | (0.143-0.209) | (0.205-0.262) | (0.246-0.31) | (0.281-0.369) | (0.276-0.35) | (0.341-0.429) | (0.187-0.258) | (0.163-0.224) | (0.215-0.284) | (0.164-0.209) | (0.152-0.202) |
| BRE | 0.276 | 0.286 | 0.247 | 0.198 | 0.282 | 0.334 | 0.279 | 0.220 | 0.233 | 0.179 | 0.151 | 0.203 | 0.193 | 0.263 | 0.218 | 0.297 | 0.279 | 0.224 | 0.391 | 0.301 | 0.309 | 0.436 | 0.171 | 0.252 | 0.234 |  | (0.138-0.215) | (0.152-0.207) | (0.206-0.278) | (0.264-0.378) | (0.2-0.274) | (0.236-0.325) | (0.162-0.215) | (0.168-0.225) | (0.155-0.235) | (0.137-0.189) | (0.149-0.203) |
| RDO | 0.170 | 0.183 | 0.173 | 0.105 | 0.189 | 0.268 | 0.208 | 0.124 | 0.106 | 0.088 | 0.066 | 0.110 | 0.076 | 0.031 | 0.026 | 0.299 | 0.199 | 0.199 | 0.474 | 0.325 | 0.324 | 0.449 | 0.020 | 0.209 | 0.175 | 0.172 |  | (0.067-0.154) | (0.126-0.212) | (0.147-0.245) | (0.138-0.243) | (0.197-0.291) | (0.074-0.194) | (0.076-0.157) | (-0.005-0.055) | (0.06-0.116) | (0.053-0.12) |
| LDO | 0.227 | 0.225 | 0.221 | 0.114 | 0.158 | 0.251 | 0.231 | 0.146 | 0.134 | 0.187 | 0.116 | 0.134 | 0.063 | 0.147 | 0.136 | 0.222 | 0.285 | 0.248 | 0.312 | 0.218 | 0.280 | 0.295 | 0.131 | 0.236 | 0.232 | 0.180 | 0.108 |  | (0.219-0.29) | (0.251-0.34) | (0.111-0.18) | (0.151-0.248) | (-0.004-0.026) | (0.003-0.027) | (0.103-0.173) | (0.126-0.179) | (0.111-0.166) |
| ENO_96_ | 0.068 | 0.026 | 0.025 | 0.189 | 0.194 | 0.336 | 0.248 | 0.147 | 0.178 | 0.167 | 0.157 | 0.129 | 0.150 | 0.172 | 0.151 | 0.198 | 0.018 | 0.017 | 0.410 | 0.253 | 0.335 | 0.504 | 0.150 | 0.286 | 0.277 | 0.239 | 0.168 | 0.254 |  | (-0.011-0.029) | (0.208-0.312) | (0.187-0.251) | (0.22-0.311) | (0.222-0.295) | (0.145-0.222) | (0.102-0.168) | (0.123-0.201) |
| ENO_11-12_ | 0.037 | 0.017 | 0.023 | 0.220 | 0.199 | 0.340 | 0.255 | 0.193 | 0.168 | 0.199 | 0.183 | 0.145 | 0.181 | 0.171 | 0.163 | 0.276 | 0.016 | 0.053 | 0.489 | 0.338 | 0.407 | 0.562 | 0.196 | 0.340 | 0.324 | 0.324 | 0.194 | 0.297 | 0.005 |  | (0.242-0.369) | (0.197-0.298) | (0.263-0.366) | (0.266-0.343) | (0.165-0.27) | (0.13-0.229) | (0.144-0.258) |
| TWA | 0.253 | 0.320 | 0.295 | 0.186 | 0.244 | 0.342 | 0.279 | 0.180 | 0.241 | 0.286 | 0.284 | 0.193 | 0.165 | 0.223 | 0.221 | 0.202 | 0.297 | 0.172 | 0.312 | 0.200 | 0.217 | 0.241 | 0.248 | 0.345 | 0.313 | 0.235 | 0.187 | 0.143 | 0.254 | 0.303 |  | (0.158-0.257) | (0.098-0.182) | (0.075-0.129) | (0.144-0.222) | (0.221-0.308) | (0.18-0.26) |
| DRY | 0.206 | 0.231 | 0.224 | 0.229 | 0.203 | 0.359 | 0.267 | 0.292 | 0.257 | 0.313 | 0.308 | 0.223 | 0.175 | 0.220 | 0.207 | 0.247 | 0.277 | 0.252 | 0.352 | 0.241 | 0.278 | 0.335 | 0.275 | 0.411 | 0.387 | 0.281 | 0.243 | 0.199 | 0.219 | 0.246 | 0.204 |  | (0.124-0.226) | (0.137-0.22) | (0.16-0.241) | (0.287-0.369) | (0.257-0.344) |
| RIE | 0.257 | 0.232 | 0.223 | 0.123 | 0.123 | 0.232 | 0.239 | 0.137 | 0.150 | 0.191 | 0.124 | 0.136 | 0.057 | 0.185 | 0.137 | 0.190 | 0.285 | 0.254 | 0.258 | 0.170 | 0.260 | 0.243 | 0.126 | 0.239 | 0.219 | 0.189 | 0.132 | 0.008 | 0.265 | 0.314 | 0.137 | 0.175 |  | (0-0.035) | (0-0.035) | (0-0.035) | (0-0.035) |
| MAC | 0.267 | 0.265 | 0.260 | 0.120 | 0.134 | 0.249 | 0.243 | 0.155 | 0.181 | 0.236 | 0.131 | 0.141 | 0.090 | 0.179 | 0.173 | 0.201 | 0.268 | 0.233 | 0.271 | 0.183 | 0.239 | 0.278 | 0.159 | 0.215 | 0.191 | 0.195 | 0.117 | 0.013 | 0.257 | 0.304 | 0.100 | 0.177 | 0.015 |  | (0.11-0.183) | (0.154-0.211) | (0.115-0.19) |
| ANN | 0.193 | 0.211 | 0.183 | 0.104 | 0.143 | 0.260 | 0.177 | 0.126 | 0.122 | 0.109 | 0.091 | 0.133 | 0.080 | 0.046 | 0.023 | 0.244 | 0.202 | 0.181 | 0.424 | 0.270 | 0.227 | 0.355 | 0.063 | 0.273 | 0.250 | 0.195 | 0.021 | 0.137 | 0.181 | 0.213 | 0.180 | 0.199 | 0.127 | 0.144 |  | (0.075-0.156) | (0.064-0.132) |
| LEV | 0.228 | 0.182 | 0.153 | 0.173 | 0.207 | 0.238 | 0.230 | 0.123 | 0.157 | 0.071 | 0.047 | 0.140 | 0.133 | 0.098 | 0.099 | 0.284 | 0.183 | 0.152 | 0.432 | 0.299 | 0.275 | 0.435 | 0.059 | 0.210 | 0.186 | 0.162 | 0.086 | 0.152 | 0.133 | 0.175 | 0.264 | 0.328 | 0.158 | 0.183 | 0.109 |  | (0.223-0.302) |
| HOW | 0.260 | 0.203 | 0.170 | 0.163 | 0.212 | 0.271 | 0.224 | 0.096 | 0.154 | 0.076 | 0.050 | 0.133 | 0.109 | 0.106 | 0.108 | 0.306 | 0.198 | 0.190 | 0.444 | 0.318 | 0.262 | 0.375 | 0.071 | 0.222 | 0.177 | 0.175 | 0.085 | 0.138 | 0.159 | 0.198 | 0.219 | 0.299 | 0.123 | 0.152 | 0.094 | 0.033 |  |
| *F*_ST_ |  |  |  |  |  |  |  |  |  |  |  |  |  |  |  |  |  |  |  |  |  |  |  |  |  |  |  |  |  |  |  |  |  |  |  |  |  |
| GRA1 |  | (0.013-0.045) | (0.02-0.045) | (0.107-0.145) | (0.106-0.151) | (0.222-0.27) | (0.128-0.166) | (0.107-0.151) | (0.072-0.113) | (0.098-0.135) | (0.09-0.138) | (0.072-0.111) | (0.07-0.105) | (0.067-0.097) | (0.058-0.091) | (0.195-0.268) | (0.071-0.12) | (0.061-0.125) | (0.47-0.513) | (0.229-0.315) | (0.238-0.284) | (0.372-0.422) | (0.074-0.111) | (0.277-0.331) | (0.208-0.263) | (0.172-0.224) | (0.058-0.098) | (0.108-0.16) | (0.036-0.079) | (0.022-0.085) | (0.175-0.228) | (0.137-0.191) | (0.134-0.195) | (0.139-0.186) | (0.074-0.114) | (0.097-0.135) | (0.109-0.149) |
| GRA2 | 0.027 |  | (0.001-0.011) | (0.111-0.14) | (0.125-0.163) | (0.215-0.245) | (0.142-0.175) | (0.104-0.14) | (0.086-0.107) | (0.084-0.108) | (0.081-0.118) | (0.073-0.098) | (0.082-0.103) | (0.083-0.105) | (0.072-0.099) | (0.169-0.232) | (0.029-0.051) | (0.038-0.1) | (0.403-0.431) | (0.207-0.278) | (0.236-0.274) | (0.363-0.396) | (0.081-0.111) | (0.262-0.301) | (0.205-0.24) | (0.169-0.207) | (0.073-0.1) | (0.119-0.152) | (0.01-0.036) | (0.006-0.04) | (0.189-0.234) | (0.157-0.208) | (0.141-0.184) | (0.151-0.181) | (0.085-0.121) | (0.085-0.115) | (0.099-0.129) |
| GRA3 | 0.031 | 0.005 |  | (0.111-0.133) | (0.119-0.148) | (0.196-0.214) | (0.135-0.158) | (0.104-0.131) | (0.083-0.097) | (0.082-0.097) | (0.078-0.105) | (0.07-0.089) | (0.081-0.094) | (0.083-0.101) | (0.07-0.094) | (0.154-0.208) | (0.039-0.055) | (0.029-0.084) | (0.36-0.377) | (0.189-0.246) | (0.204-0.231) | (0.315-0.339) | (0.075-0.096) | (0.236-0.262) | (0.187-0.21) | (0.14-0.166) | (0.073-0.094) | (0.117-0.139) | (0.015-0.03) | (0.012-0.045) | (0.173-0.211) | (0.147-0.187) | (0.133-0.168) | (0.142-0.162) | (0.079-0.111) | (0.076-0.097) | (0.09-0.113) |
| DEU | 0.125 | 0.126 | 0.123 |  | (0.039-0.067) | (0.157-0.185) | (0.1-0.13) | (0.102-0.134) | (0.08-0.102) | (0.086-0.114) | (0.082-0.119) | (0.08-0.107) | (0.057-0.078) | (0.071-0.096) | (0.053-0.084) | (0.171-0.218) | (0.132-0.162) | (0.103-0.166) | (0.354-0.381) | (0.178-0.238) | (0.176-0.211) | (0.259-0.294) | (0.066-0.097) | (0.239-0.283) | (0.196-0.235) | (0.149-0.184) | (0.051-0.088) | (0.085-0.113) | (0.116-0.145) | (0.127-0.168) | (0.148-0.192) | (0.16-0.212) | (0.091-0.129) | (0.095-0.125) | (0.05-0.078) | (0.077-0.108) | (0.086-0.121) |
| KEN | 0.128 | 0.144 | 0.133 | 0.052 |  | (0.15-0.184) | (0.098-0.13) | (0.121-0.169) | (0.08-0.11) | (0.112-0.155) | (0.104-0.158) | (0.088-0.121) | (0.059-0.086) | (0.087-0.125) | (0.069-0.108) | (0.184-0.231) | (0.15-0.19) | (0.112-0.178) | (0.368-0.401) | (0.183-0.25) | (0.158-0.197) | (0.24-0.278) | (0.086-0.129) | (0.264-0.321) | (0.21-0.26) | (0.178-0.224) | (0.082-0.128) | (0.125-0.162) | (0.129-0.167) | (0.133-0.185) | (0.181-0.24) | (0.16-0.211) | (0.121-0.168) | (0.12-0.157) | (0.068-0.109) | (0.109-0.152) | (0.113-0.113) |
| DUN | 0.243 | 0.230 | 0.205 | 0.170 | 0.167 |  | (0.105-0.133) | (0.201-0.244) | (0.15-0.168) | (0.202-0.229) | (0.209-0.25) | (0.152-0.178) | (0.134-0.155) | (0.179-0.206) | (0.17-0.208) | (0.272-0.318) | (0.272-0.304) | (0.223-0.299) | (0.459-0.487) | (0.277-0.339) | (0.285-0.319) | (0.337-0.37) | (0.188-0.232) | (0.383-0.426) | (0.299-0.337) | (0.274-0.308) | (0.175-0.22) | (0.216-0.246) | (0.232-0.265) | (0.263-0.308) | (0.279-0.335) | (0.278-0.333) | (0.213-0.273) | (0.227-0.255) | (0.17-0.217) | (0.196-0.229) | (0.206-0.241) |
| HAR | 0.147 | 0.159 | 0.146 | 0.115 | 0.114 | 0.119 |  | (0.129-0.163) | (0.118-0.141) | (0.137-0.169) | (0.106-0.143) | (0.121-0.149) | (0.092-0.116) | (0.11-0.137) | (0.088-0.12) | (0.213-0.26) | (0.181-0.217) | (0.152-0.216) | (0.384-0.416) | (0.227-0.288) | (0.241-0.278) | (0.302-0.337) | (0.099-0.136) | (0.282-0.323) | (0.222-0.26) | (0.186-0.225) | (0.094-0.13) | (0.147-0.181) | (0.145-0.178) | (0.158-0.201) | (0.196-0.248) | (0.185-0.237) | (0.145-0.198) | (0.16-0.194) | (0.088-0.124) | (0.123-0.154) | (0.129-0.163) |
| INV | 0.128 | 0.121 | 0.117 | 0.117 | 0.144 | 0.221 | 0.146 |  | (0.098-0.125) | (0.062-0.088) | (0.079-0.122) | (0.087-0.119) | (0.076-0.102) | (0.083-0.107) | (0.08-0.111) | (0.177-0.229) | (0.131-0.162) | (0.108-0.18) | (0.386-0.418) | (0.184-0.255) | (0.207-0.25) | (0.339-0.383) | (0.06-0.089) | (0.246-0.289) | (0.189-0.231) | (0.16-0.204) | (0.063-0.098) | (0.108-0.148) | (0.102-0.134) | (0.121-0.173) | (0.159-0.207) | (0.182-0.238) | (0.113-0.165) | (0.126-0.168) | (0.064-0.095) | (0.079-0.11) | (0.066-0.096) |
| DEE | 0.090 | 0.096 | 0.090 | 0.091 | 0.095 | 0.159 | 0.129 | 0.110 |  | (0.065-0.084) | (0.065-0.097) | (0.031-0.046) | (0.031-0.042) | (0.044-0.059) | (0.04-0.063) | (0.169-0.212) | (0.111-0.136) | (0.079-0.131) | (0.335-0.351) | (0.179-0.23) | (0.164-0.192) | (0.265-0.289) | (0.052-0.079) | (0.193-0.224) | (0.137-0.166) | (0.14-0.164) | (0.041-0.068) | (0.09-0.112) | (0.094-0.12) | (0.099-0.136) | (0.142-0.176) | (0.135-0.184) | (0.094-0.13) | (0.101-0.123) | (0.048-0.075) | (0.08-0.102) | (0.079-0.104) |
| SHI | 0.115 | 0.095 | 0.089 | 0.099 | 0.133 | 0.215 | 0.154 | 0.074 | 0.074 |  | (0.036-0.067) | (0.067-0.09) | (0.059-0.077) | (0.039-0.055) | (0.038-0.059) | (0.174-0.223) | (0.106-0.129) | (0.081-0.133) | (0.36-0.389) | (0.188-0.25) | (0.185-0.223) | (0.315-0.354) | (0.019-0.037) | (0.183-0.221) | (0.13-0.163) | (0.095-0.13) | (0.031-0.056) | (0.094-0.12) | (0.079-0.108) | (0.088-0.141) | (0.154-0.19) | (0.158-0.209) | (0.097-0.137) | (0.12-0.15) | (0.038-0.065) | (0.032-0.05) | (0.036-0.057) |
| MAN | 0.113 | 0.099 | 0.090 | 0.099 | 0.130 | 0.228 | 0.123 | 0.100 | 0.080 | 0.050 |  | (0.056-0.093) | (0.05-0.077) | (0.039-0.063) | (0.031-0.059) | (0.179-0.245) | (0.094-0.13) | (0.084-0.15) | (0.419-0.468) | (0.204-0.28) | (0.229-0.277) | (0.336-0.389) | (0.015-0.045) | (0.184-0.229) | (0.134-0.174) | (0.094-0.134) | (0.019-0.05) | (0.087-0.125) | (0.07-0.106) | (0.082-0.137) | (0.154-0.207) | (0.161-0.223) | (0.085-0.137) | (0.115-0.154) | (0.03-0.066) | (0.016-0.04) | (0.016-0.043) |
| FLE1 | 0.090 | 0.085 | 0.079 | 0.094 | 0.104 | 0.165 | 0.135 | 0.102 | 0.038 | 0.078 | 0.073 |  | (0.021-0.035) | (0.052-0.071) | (0.044-0.071) | (0.138-0.185) | (0.095-0.125) | (0.06-0.11) | (0.323-0.35) | (0.15-0.208) | (0.171-0.21) | (0.283-0.317) | (0.051-0.08) | (0.204-0.245) | (0.145-0.18) | (0.135-0.167) | (0.043-0.073) | (0.076-0.104) | (0.07-0.097) | (0.087-0.128) | (0.125-0.163) | (0.13-0.184) | (0.072-0.11) | (0.084-0.11) | (0.053-0.089) | (0.068-0.095) | (0.065-0.092) |
| FLE2 | 0.086 | 0.092 | 0.088 | 0.067 | 0.072 | 0.144 | 0.104 | 0.089 | 0.036 | 0.068 | 0.062 | 0.028 |  | (0.038-0.054) | (0.027-0.047) | (0.128-0.167) | (0.109-0.131) | (0.064-0.122) | (0.289-0.308) | (0.132-0.181) | (0.138-0.168) | (0.222-0.248) | (0.037-0.063) | (0.173-0.205) | (0.123-0.152) | (0.111-0.138) | (0.025-0.053) | (0.043-0.061) | (0.08-0.104) | (0.098-0.132) | (0.101-0.133) | (0.094-0.143) | (0.039-0.067) | (0.054-0.071) | (0.03-0.053) | (0.058-0.077) | (0.055-0.078) |
| WOF | 0.081 | 0.094 | 0.092 | 0.083 | 0.105 | 0.192 | 0.123 | 0.095 | 0.051 | 0.047 | 0.051 | 0.061 | 0.046 |  | (0.008-0.02) | (0.157-0.204) | (0.094-0.117) | (0.078-0.122) | (0.332-0.355) | (0.175-0.231) | (0.171-0.201) | (0.265-0.295) | (0.015-0.037) | (0.16-0.191) | (0.119-0.146) | (0.129-0.162) | (0.007-0.023) | (0.086-0.111) | (0.077-0.098) | (0.08-0.117) | (0.112-0.141) | (0.109-0.152) | (0.086-0.128) | (0.103-0.13) | (0.014-0.034) | (0.041-0.057) | (0.04-0.063) |
| PAL | 0.073 | 0.084 | 0.081 | 0.068 | 0.087 | 0.189 | 0.104 | 0.095 | 0.051 | 0.048 | 0.043 | 0.057 | 0.037 | 0.013 |  | (0.151-0.203) | (0.085-0.114) | (0.064-0.117) | (0.353-0.387) | (0.17-0.235) | (0.168-0.207) | (0.283-0.326) | (0.016-0.041) | (0.173-0.215) | (0.128-0.166) | (0.123-0.162) | (0.003-0.024) | (0.076-0.108) | (0.065-0.093) | (0.072-0.108) | (0.121-0.159) | (0.11-0.16) | (0.074-0.123) | (0.094-0.13) | (0.003-0.027) | (0.033-0.057) | (0.039-0.068) |
| VAL | 0.229 | 0.199 | 0.179 | 0.193 | 0.207 | 0.294 | 0.236 | 0.201 | 0.190 | 0.198 | 0.211 | 0.161 | 0.147 | 0.180 | 0.177 |  | (0.159-0.231) | (0.051-0.196) | (0.115-0.177) | (-0.001-0.03) | (0.222-0.271) | (0.351-0.399) | (0.149-0.201) | (0.315-0.381) | (0.266-0.323) | (0.231-0.283) | (0.154-0.221) | (0.165-0.208) | (0.145-0.208) | (0.201-0.29) | (0.215-0.282) | (0.221-0.289) | (0.167-0.222) | (0.179-0.226) | (0.143-0.204) | (0.163-0.215) | (0.182-0.238) |
| NAR_00-02_ | 0.092 | 0.040 | 0.047 | 0.148 | 0.171 | 0.288 | 0.198 | 0.146 | 0.123 | 0.117 | 0.111 | 0.109 | 0.119 | 0.105 | 0.099 | 0.194 |  | (0.012-0.082) | (0.428-0.458) | (0.209-0.29) | (0.251-0.294) | (0.399-0.435) | (0.101-0.128) | (0.265-0.303) | (0.216-0.249) | (0.201-0.236) | (0.092-0.126) | (0.17-0.2) | (0.011-0.032) | (0.01-0.05) | (0.209-0.255) | (0.196-0.243) | (0.188-0.235) | (0.189-0.222) | (0.097-0.138) | (0.102-0.134) | (0.11-0.143) |
| NAR_12_ | 0.090 | 0.064 | 0.053 | 0.130 | 0.141 | 0.258 | 0.180 | 0.139 | 0.101 | 0.104 | 0.115 | 0.081 | 0.089 | 0.097 | 0.088 | 0.121 | 0.043 |  | (0.379-0.558) | (0.084-0.265) | (0.171-0.249) | (0.37-0.447) | (0.069-0.122) | (0.263-0.337) | (0.202-0.26) | (0.156-0.222) | (0.071-0.131) | (0.116-0.192) | (0.008-0.068) | (0.029-0.116) | (0.134-0.221) | (0.172-0.251) | (0.128-0.224) | (0.132-0.216) | (0.068-0.129) | (0.079-0.136) | (0.097-0.148) |
| NEL_01_ | 0.491 | 0.417 | 0.369 | 0.368 | 0.383 | 0.472 | 0.399 | 0.402 | 0.343 | 0.374 | 0.441 | 0.336 | 0.298 | 0.344 | 0.370 | 0.147 | 0.443 | 0.474 |  | (0.082-0.142) | (0.377-0.418) | (0.519-0.562) | (0.358-0.393) | (0.544-0.594) | (0.477-0.517) | (0.419-0.462) | (0.374-0.424) | (0.33-0.364) | (0.395-0.424) | (0.539-0.589) | (0.454-0.518) | (0.404-0.466) | (0.396-0.449) | (0.358-0.391) | (0.353-0.393) | (0.364-0.4) | (0.39-0.427) |
| NEL_11_ | 0.270 | 0.242 | 0.217 | 0.206 | 0.216 | 0.308 | 0.255 | 0.219 | 0.204 | 0.218 | 0.241 | 0.178 | 0.156 | 0.202 | 0.201 | 0.012 | 0.248 | 0.173 | 0.110 |  | (0.223-0.284) | (0.357-0.412) | (0.168-0.233) | (0.34-0.417) | (0.287-0.353) | (0.245-0.309) | (0.175-0.254) | (0.165-0.219) | (0.186-0.263) | (0.25-0.346) | (0.216-0.303) | (0.236-0.321) | (0.162-0.233) | (0.17-0.229) | (0.164-0.232) | (0.177-0.242) | (0.198-0.268) |
| RGL | 0.260 | 0.255 | 0.218 | 0.193 | 0.177 | 0.301 | 0.259 | 0.229 | 0.178 | 0.204 | 0.252 | 0.190 | 0.153 | 0.185 | 0.187 | 0.246 | 0.271 | 0.208 | 0.398 | 0.253 |  | (0.191-0.23) | (0.18-0.224) | (0.368-0.412) | (0.307-0.351) | (0.245-0.288) | (0.184-0.236) | (0.197-0.235) | (0.221-0.261) | (0.269-0.324) | (0.218-0.274) | (0.23-0.288) | (0.212-0.26) | (0.201-0.24) | (0.152-0.193) | (0.196-0.236) | (0.185-0.229) |
| LGL | 0.396 | 0.380 | 0.327 | 0.276 | 0.259 | 0.352 | 0.319 | 0.361 | 0.277 | 0.334 | 0.361 | 0.299 | 0.235 | 0.280 | 0.305 | 0.376 | 0.417 | 0.405 | 0.540 | 0.384 | 0.210 |  | (0.299-0.346) | (0.504-0.549) | (0.44-0.485) | (0.373-0.413) | (0.295-0.347) | (0.285-0.321) | (0.364-0.399) | (0.438-0.485) | (0.325-0.387) | (0.344-0.397) | (0.318-0.371) | (0.296-0.337) | (0.261-0.308) | (0.304-0.346) | (0.299-0.344) |
| GIR | 0.091 | 0.095 | 0.084 | 0.081 | 0.107 | 0.209 | 0.116 | 0.073 | 0.065 | 0.027 | 0.029 | 0.064 | 0.049 | 0.025 | 0.027 | 0.173 | 0.114 | 0.092 | 0.374 | 0.199 | 0.201 | 0.321 |  | (0.14-0.183) | (0.095-0.131) | (0.084-0.123) | (0.004-0.031) | (0.074-0.103) | (0.067-0.095) | (0.08-0.122) | (0.12-0.166) | (0.129-0.185) | (0.07-0.112) | (0.095-0.128) | (0.014-0.044) | (0.019-0.042) | (0.021-0.048) |
| EYE | 0.303 | 0.281 | 0.249 | 0.261 | 0.291 | 0.405 | 0.302 | 0.267 | 0.208 | 0.201 | 0.207 | 0.224 | 0.189 | 0.176 | 0.194 | 0.347 | 0.283 | 0.298 | 0.569 | 0.378 | 0.391 | 0.526 | 0.161 |  | (0.032-0.065) | (0.239-0.284) | (0.158-0.206) | (0.202-0.244) | (0.236-0.278) | (0.288-0.346) | (0.316-0.371) | (0.303-0.372) | (0.223-0.282) | (0.23-0.28) | (0.191-0.233) | (0.173-0.208) | (0.189-0.229) |
| COR | 0.234 | 0.222 | 0.199 | 0.216 | 0.235 | 0.318 | 0.240 | 0.209 | 0.151 | 0.146 | 0.153 | 0.163 | 0.138 | 0.133 | 0.146 | 0.293 | 0.232 | 0.229 | 0.497 | 0.319 | 0.329 | 0.462 | 0.112 | 0.048 |  | (0.184-0.228) | (0.108-0.151) | (0.163-0.202) | (0.191-0.226) | (0.223-0.282) | (0.27-0.319) | (0.255-0.321) | (0.174-0.224) | (0.184-0.228) | (0.141-0.187) | (0.124-0.156) | (0.131-0.165) |
| BRE | 0.197 | 0.187 | 0.153 | 0.166 | 0.199 | 0.291 | 0.205 | 0.182 | 0.153 | 0.112 | 0.113 | 0.151 | 0.124 | 0.145 | 0.141 | 0.256 | 0.218 | 0.186 | 0.441 | 0.276 | 0.267 | 0.393 | 0.102 | 0.261 | 0.204 |  | (0.103-0.148) | (0.124-0.157) | (0.151-0.195) | (0.19-0.257) | (0.209-0.262) | (0.198-0.264) | (0.144-0.193) | (0.153-0.186) | (0.108-0.15) | (0.099-0.134) | (0.103-0.138) |
| RDO | 0.076 | 0.085 | 0.083 | 0.067 | 0.104 | 0.196 | 0.112 | 0.079 | 0.053 | 0.042 | 0.033 | 0.057 | 0.037 | 0.014 | 0.012 | 0.185 | 0.106 | 0.097 | 0.398 | 0.214 | 0.209 | 0.319 | 0.016 | 0.181 | 0.128 | 0.124 |  | (0.052-0.102) | (0.066-0.098) | (0.074-0.117) | (0.102-0.152) | (0.111-0.173) | (0.056-0.117) | (0.072-0.124) | (-0.002-0.026) | (0.025-0.052) | (0.027-0.057) |
| LDO | 0.133 | 0.135 | 0.128 | 0.099 | 0.142 | 0.231 | 0.163 | 0.127 | 0.101 | 0.106 | 0.104 | 0.090 | 0.052 | 0.098 | 0.092 | 0.186 | 0.185 | 0.149 | 0.347 | 0.191 | 0.216 | 0.303 | 0.087 | 0.223 | 0.183 | 0.141 | 0.074 |  | (0.132-0.167) | (0.161-0.205) | (0.111-0.156) | (0.125-0.198) | (0.001-0.028) | (0.022-0.046) | (0.067-0.104) | (0.084-0.11) | (0.092-0.123) |
| ENO_96_ | 0.054 | 0.021 | 0.021 | 0.130 | 0.148 | 0.248 | 0.161 | 0.117 | 0.105 | 0.092 | 0.087 | 0.083 | 0.091 | 0.087 | 0.078 | 0.174 | 0.020 | 0.034 | 0.409 | 0.224 | 0.240 | 0.381 | 0.080 | 0.256 | 0.208 | 0.171 | 0.081 | 0.149 |  | (-0.005-0.033) | (0.166-0.213) | (0.156-0.204) | (0.149-0.194) | (0.155-0.191) | (0.074-0.112) | (0.069-0.101) | (0.08-0.113) |
| ENO_11-12_ | 0.049 | 0.022 | 0.026 | 0.146 | 0.157 | 0.284 | 0.179 | 0.144 | 0.117 | 0.113 | 0.107 | 0.106 | 0.115 | 0.097 | 0.088 | 0.241 | 0.028 | 0.067 | 0.562 | 0.296 | 0.297 | 0.461 | 0.099 | 0.315 | 0.251 | 0.224 | 0.094 | 0.183 | 0.011 |  | (0.2-0.265) | (0.19-0.256) | (0.179-0.244) | (0.189-0.233) | (0.09-0.136) | (0.085-0.138) | (0.101-0.15) |
| TWA | 0.198 | 0.210 | 0.191 | 0.168 | 0.208 | 0.306 | 0.221 | 0.182 | 0.157 | 0.171 | 0.179 | 0.144 | 0.115 | 0.126 | 0.139 | 0.246 | 0.231 | 0.175 | 0.485 | 0.257 | 0.244 | 0.354 | 0.140 | 0.343 | 0.294 | 0.234 | 0.125 | 0.133 | 0.188 | 0.231 |  | (0.167-0.243) | (0.113-0.173) | (0.1-0.148) | (0.105-0.147) | (0.141-0.187) | (0.133-0.188) |
| DRY | 0.163 | 0.181 | 0.167 | 0.186 | 0.184 | 0.306 | 0.211 | 0.209 | 0.159 | 0.183 | 0.191 | 0.157 | 0.118 | 0.130 | 0.133 | 0.255 | 0.219 | 0.207 | 0.433 | 0.274 | 0.258 | 0.371 | 0.156 | 0.339 | 0.288 | 0.231 | 0.142 | 0.161 | 0.179 | 0.220 | 0.203 |  | (0.128-0.213) | (0.126-0.196) | (0.109-0.165) | (0.16-0.213) | (0.158-0.218) |
| RIE | 0.162 | 0.161 | 0.149 | 0.109 | 0.142 | 0.242 | 0.169 | 0.138 | 0.110 | 0.116 | 0.109 | 0.090 | 0.051 | 0.105 | 0.096 | 0.194 | 0.209 | 0.171 | 0.422 | 0.194 | 0.235 | 0.343 | 0.090 | 0.251 | 0.198 | 0.167 | 0.086 | 0.013 | 0.171 | 0.209 | 0.141 | 0.169 |  | (0.003-0.038) | (0.067-0.117) | (0.083-0.125) | (0.087-0.135) |
| MAC | 0.162 | 0.165 | 0.153 | 0.110 | 0.136 | 0.241 | 0.176 | 0.146 | 0.112 | 0.136 | 0.134 | 0.097 | 0.062 | 0.117 | 0.111 | 0.202 | 0.206 | 0.169 | 0.375 | 0.199 | 0.221 | 0.315 | 0.111 | 0.254 | 0.205 | 0.170 | 0.097 | 0.033 | 0.172 | 0.210 | 0.123 | 0.161 | 0.018 |  | (0.082-0.121) | (0.111-0.142) | (0.104-0.144) |
| ANN | 0.092 | 0.101 | 0.094 | 0.063 | 0.087 | 0.191 | 0.105 | 0.079 | 0.061 | 0.050 | 0.046 | 0.069 | 0.039 | 0.023 | 0.013 | 0.173 | 0.117 | 0.095 | 0.371 | 0.197 | 0.171 | 0.284 | 0.028 | 0.211 | 0.163 | 0.128 | 0.010 | 0.085 | 0.092 | 0.111 | 0.125 | 0.137 | 0.088 | 0.099 |  | (0.035-0.069) | (0.034-0.066) |
| LEV | 0.114 | 0.099 | 0.086 | 0.092 | 0.130 | 0.212 | 0.139 | 0.093 | 0.090 | 0.041 | 0.027 | 0.081 | 0.067 | 0.049 | 0.044 | 0.187 | 0.117 | 0.104 | 0.382 | 0.209 | 0.215 | 0.324 | 0.029 | 0.190 | 0.140 | 0.116 | 0.037 | 0.097 | 0.084 | 0.108 | 0.163 | 0.186 | 0.103 | 0.127 | 0.049 |  | (0.109-0.149) |
| HOW | 0.127 | 0.113 | 0.100 | 0.103 | 0.138 | 0.223 | 0.146 | 0.081 | 0.091 | 0.046 | 0.028 | 0.078 | 0.065 | 0.051 | 0.052 | 0.210 | 0.126 | 0.121 | 0.408 | 0.232 | 0.206 | 0.321 | 0.033 | 0.209 | 0.148 | 0.120 | 0.041 | 0.107 | 0.095 | 0.123 | 0.159 | 0.186 | 0.108 | 0.123 | 0.048 | 0.021 |  |
|  |  |  |  |  |  |  |  |  |  |  |  |  |  |  |  |  |  |  |  |  |  |  |  |  |  |  |  |  |  |  |  |  |  |  |  |  |  |
